# Aminofullerenes as targeted inhibitors of EGFR: from pancreatic cancer inhibitors to *Drosophila m*. Toxicology

**DOI:** 10.1080/17435889.2025.2461985

**Published:** 2025-02-07

**Authors:** Katarzyna Malarz, Julia Korzuch, Anna Mrozek-Wilczkiewicz, Magdalena Szubka, Patryk Rurka, Karol Małota, Aitor Herraiz, Dominik Dreszer, Karina Kocot, Fernando Herranz, Magdalena Rost-Roszkowska, Tao Sun, Robert Musioł, Maciej Serda

**Affiliations:** a Department of Systems Biology and Engineering, Silesian University of Technology, Gliwice, Poland; b Institute of Physics, Faculty of Science and Technology, University of Silesia in Katowice, Chorzów, Poland; c Institute of Chemistry, Faculty of Science and Technology, University of Silesia in Katowice, Katowice, Poland; d Institute of Biology, Biotechnology and Environmental Protection, Faculty of Natural Sciences, University of Silesia in Katowice, Katowice, Poland; e Instituto de Química Médica, Consejo Superior de Investigaciones Científicas (IQM-CSIC), Madrid, Spain; f Key Laboratory of Smart Drug Delivery Ministry of Education, Department of Pharmaceutics, School of Pharmacy, Fudan University, Shanghai, China

**Keywords:** Fullerene, aminofullerene, pancreatic cancer, EGFR inhibitors, drosophila, cancer nanotechnology

## Abstract

**Aim:**

Pancreatic ductal adenocarcinoma (PDAC) is recognized as one of the most formidable cancers, largely due to its distinct microenvironment characterized predominantly by extensive desmoplastic stroma. In this study, we synthesized three novel water-soluble fullerene-based nanomaterials targeting EGFR protein.

**Methods:**

The direct amination of fullerene carbon atoms, was followed by conjugation with a modified derivative of the EGFR inhibitor-erlotinib, resulting in the formation of novel water-soluble fullerene derivatives.

**Results:**

Further investigation into PAN02 and AsPC-1 cell lines revealed that these fullerene nanomaterials could induce cell cycle arrest in the G0/G1 phase, corroborated by alterations in the expression levels of the p27 and cyclin E1 proteins. Additionally, mechanisms of cell death were identified as autophagy for C_60_BUT and C_70_BUT-ERL, and apoptosis for Gd@C_82_EDA-ERL nanomaterials.

**Conclusions:**

Crucially, the study uncovered the efficacy of synthesized aminofullerenes in inhibiting the EGFR signaling pathway. The further toxicological studies of Gd@C_82_EDA-ERL fullerene on *Drosophila melanogaster*, underscored its potential for theranostic applications.

## GRAPHICAL ABSTRACT

**Figure:**
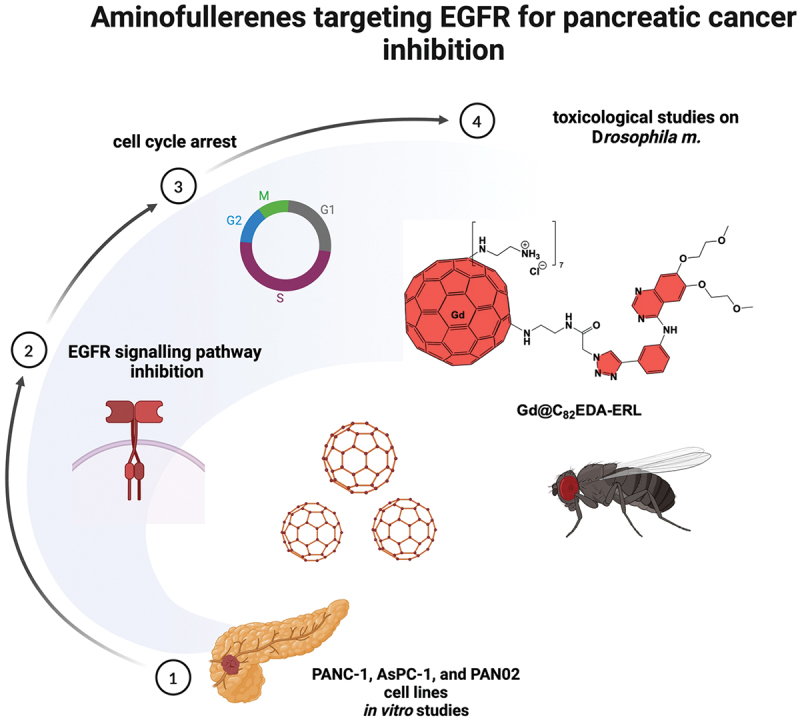

## 1. Background

Pancreatic ductal adenocarcinoma (PDA) is a major contributor to cancer-related deaths worldwide, with a significant number of cases being locally advanced and thus not amenable to surgical excision [1,2]. This malignancy is notoriously resistant to a plethora of therapeutic modalities, including chemotherapy, radiotherapy, and targeted pharmaceuticals [3]. Its resistance can be attributed to factors such as an intricate stromal environment, diminished immunogenicity, and epigenetic modifications of the parenchymal cells [4]. Conventionally, the clinical management of pancreatic cancer involves the surgical excision of the tumor, supplemented with chemoradiotherapy [5]. Chemotherapy frequently involves the usage of multiple small molecules and natural products, such as gemcitabine, paclitaxel, cisplatin, and the epidermal growth factor receptor (EGFR) inhibitor erlotinib [6].

The EGFR belongs to the tyrosine receptor kinase family and plays an instrumental role in orchestrating a myriad of cellular activities [7]. The overexpression of EGFR is a recurrent phenomenon in pancreatic cancer, correlating with the malignancy’s aggressive disposition and its resistance to treatment. Erlotinib is a first-generation EGFR kinase inhibitor that was approved by the Food and Drug Administration (FDA) in 2016 for pancreatic cancer treatment. It works by binding to the ATP-binding site of the EGFR kinase domain, thus preventing ATP association [8]. By inhibiting EGFR kinase activity, erlotinib disrupts the signaling networks that promote the growth and survival of cancer cells, thereby potentially slowing down the progression of pancreatic cancer [9].

Interestingly, a range of nanotherapeutic formulations have been specifically engineered for the management of PDA. In recent years, the FDA has approved two notable formulations. In 2013, Abraxane, an albumin-bound nanoformulation of paclitaxel, was approved for use alongside gemcitabine. In 2015, a liposomal version of irinotecan co-administered with 5-fluorouracil was approved for patients with metastatic PDA who exhibit limited responsiveness to gemcitabine [10,11]. Carbon nanomaterials (CNs) have garnered significant interest within the scientific community because of their exceptional optical, photothermal, and mechanical properties, alongside a versatile chemistry for covalent functionalization. The inherent hydrophobic nature of these carbonaceous nanomaterials enables them to effectively load drugs through hydrophobic interactions or π–π stacking, establishing them as efficient platforms for drug delivery [12,13]. The biocompatibility of these CNs has been enhanced through synthetic derivatization, either *via* covalent or non-covalent modifications [14]. Covalent modifications of fullerenes typically involve the addition of hydroxyl, carboxyl, or amino groups to their surface, such as in Bingel-Hirsch/Prato reactions for controlled synthesis of biologically active fullerenes [15–17].

Gadolinium-containing endohedral fullerenes have shown promising applications in various cancer therapies. They act as specific inhibitors of breast cancer stem cells and interact with several matrix metalloproteinases in human pancreatic cancer xenografts [18,19]. Additionally, the immune-related pathways through which Gd@C_82_(OH)_22_ nanoparticles impede tumor growth not only facilitate the production of *Th1* cytokine but also reduce *Th2* cytokine levels while preserving the integrity of B cells and T cells [20]. The MRI-contrast agent properties of water-soluble gadofullerenes have been extensively studied by the groups of Wilson and Wang [21,22]. The published longitudinal proton relativities for these gadofullerenes were remarkably high and varied based on their chemical modification and fullerene cage structure [23]. Several investigations consistently demonstrated that polyhydroxylated Gd@C_82_ (Gd@C_82_(OH)_x_) exhibits significantly elevated r_1_ relaxivity levels. Specifically, at magnetic field strengths of 0.5 T, the observed enhancement is in the range of 12–14 times, while at 7.0 T, it reaches six to eight times greater compared to the commercially available Magnevist, as documented in the relevant literature [24,25]. Aminated fullerene derivatives exhibit substantial potential in cancer nanotechnology, largely attributed to their facile and robust synthetic pathways mainly *via* direct (photo)amination, as well as their convenient purification by membrane dialysis to yield water-soluble cationic buckyballs. Their ability to undergo further bioconjugation with carboxylated small-molecular ligands, antibodies, or negatively charged proteins underscores their promise for translational biomedical applications [26,27]. For example, in a recent study, Chunru Wang and colleagues identified myosin heavy chain 9 (MYH9) as a critical molecular target of the aminated fullerene derivative C_70_EDA and investigated its inhibitory mechanism [28]. The C_70_EDA fullerene binds to the C-terminal region of MYH9, inducing MYH9 translocation from the cytoplasm to the cellular periphery.

Our previous studies have shown that *D*-glucosamine derivatives of [60]fullerenes had phototoxic effects on pancreatic cancer cells and accumulated mostly in the nucleus of pancreatic stellate cells [29]. Furthermore, our preceding research showed that the studied glycofullerenes were effective inhibitors of non-receptor tyrosine kinases (Fyn A and BTK) with IC_50_ values in the lower micromolar range. Additionally, we have established that the formation of a protein corona on the surface of [60]fullerene derivatives significantly alter their functional profile, thereby refining the specificity of these CNs toward Fyn A and BTK kinases. Utilizing our knowledge of fullerene and gadofullerene scaffolds in relation to their interactions with pancreatic cancer, we developed a series of water-soluble aminofullerenes based on C_60_, C_70_, and Gd@C_82_ substrates. These aminofullerenes incorporate ethylenediamine (EDA) and 1,4-diaminobutane (BUT) fragments by direct amination of fullerene core (formation of Csp^2^-N bonds). However, some of the obtained derivatives were not stable in water, or their further conjugation with erlotinib derivatives was unsuccessful. We believed that the synthesized aminofullerenes were ideal candidates for further functionalization with small molecules that target pancreatic cancer. Therefore, we modified the erlotinib molecule by performing copper-catalyzed 1,3-dipolar cycloaddition with corresponding azidoacetic acid to form a triazole derivative of erlotinib with a carboxyl function (called here ERL-COOH).

**Figure 1.**
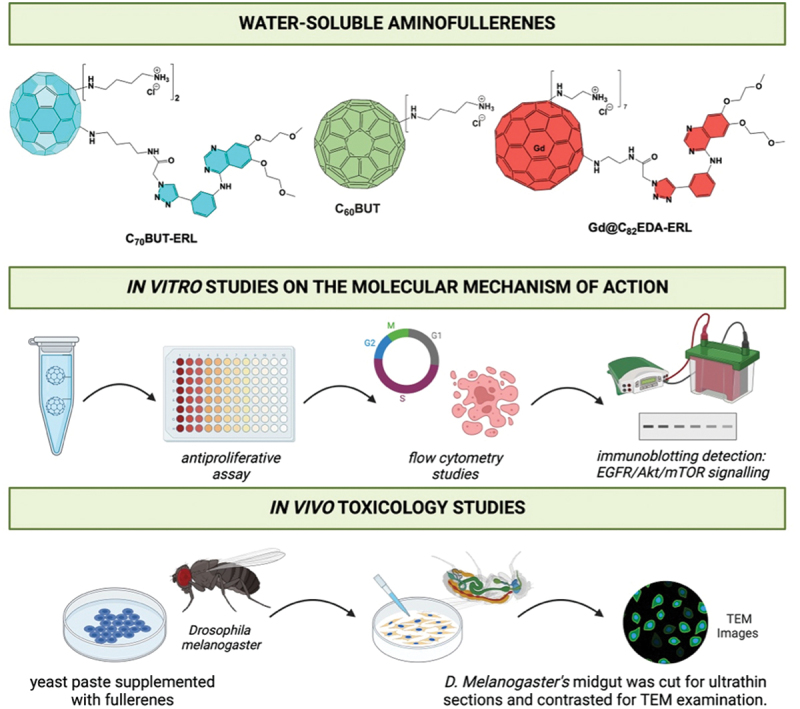
Roadmap of the current study: (A) synthesis of fullerene nanomaterials and their *in vitro* activity with mechanism of action (EGFR signaling inhibition) and *in vivo* toxicology studies. Created with Biorender^R^.

In this report, we examined the effectiveness of two nanoconjugates of fullerenes with erlotinib derivative (referred to as C_70_BUT-ERL and Gd@C_82_EDA-ERL) and one aminofullerene (referred to as C_60_BUT) in combating cancer. We focused on the high anticancer activity of C_60_BUT on pancreatic cancer cell lines, including PANC-1, AsPC-1, and PAN02. Moreover, we tested the toxicological properties of the gadofullerene nanoconjugate Gd@C_82_EDA-ERL using the *Drosophila melanogaster* model and found it to be nontoxic while also revealing information about its tissue localization. The roadmap for this study is given in Figure 1.

## 2. Experimental

### 2.1. Materials

All chemicals used were of reagent-grade quality or better. Solvents were dried following standard literature procedures. The following reagents were used as received: C_60_, C_70_ (both 99.5+%, SES Research, USA), Gd@C_82_ (Funano, P.R. China), erlotinib hydrochloride, ethylenediamine and 1,4-diaminobutane (Sigma Aldrich, Germany), toluene (Chempur, Poland), methanol (Chempur, Poland), and concentrated hydrogen chloride (Avantor, Poland). All solvents were prepared according to the corresponding literature procedures, which involved treating them with a dehydrating agent, distilling them, and then using them immediately. Triton X-100 was from Avantor Performance Materials Poland S.A. (Gliwice, Poland). The stock solution of yttrium (1000 µg/mL) was purchased from Merck Millipore (Darmstadt, Germany). The solution of silicon in isopropanol was purchased from SERVA (Heidelberg, Germany). High-purity water from the Milli-Q system (Millipore, Molsheim, France) was used for sample preparation and reagent dilutions. The final dialysis purification of the water-soluble fullerene nanomaterials was performed on MicrosepTM (Pall Corporation, USA) centrifugal membranes with molecular cutoffs of 1 and 3 kDa.

### 2.2. Characterizations

Nuclear magnetic resonance (NMR) spectra were obtained using a Bruker Avance III 500 MHz NMR spectrometer with tetramethylsilane as the internal standard. The *CHNS* elemental analysis was performed with a FlashSmart thermal analyzer (Thermo Fisher Scientific). Attenuated total reflectance Fourier transform infrared (ATR-FT-IR) measurements were collected using a JASCO FT/IR-4600 spectrophotometer equipped with a JASCO ATR PRO ONE kit. The fullerene powders were measured using an ATR ZnSe accessory in the range 700–4000 cm^−1^. The spectra were recorded using 64 accumulations at a spectral resolution of 1 cm^−1^. The dynamic light scattering of the fullerene nanomaterials was measured using a Zetasizer Nano Instrument (Malvern Panalytical Ltd., UK). The high-resolution mass spectrometry was carried out on the ESI-Q-TOF maXis impact (Bruker Daltonics Inc, USA). The X-ray photoelectron spectroscopy (XPS) spectra were collected on a PHI5700/660 Physical Electronics Photoelectron Spectrometer (Physical Electronics PHI 5700, Chanhassen, MN, USA) equipped with monochromatic Al Kα X-ray radiation (1486.6 eV). The energy of the electrons was measured with a hemispherical analyzer and a resolution of approximately 0.3 eV. The measurements of photoelectron emission were taken from a surface area with a diameter of 800 μm and at a take-off angle of 45°. The spectra were calibrated to adventitious carbon located at 284.9 eV because the sample was exposed to air. Quantification of XPS spectra utilizing peak area and peak height sensitivity factor used the Multipak Physical Electronics application (v.9.6.0.1, ULVAC PHI, Chigasaki, Japan). The XPS core level spectra were fitted with the Doniach-Sunjic method. The microstructural observations and the micro-compositional analyses were conducted using the JEOL-7600F scanning electron microscope (SEM) equipped with the Oxford X-ray energy dispersive spectroscopy (EDS) microprobe. The microprobe operated at 15 kV accelerating voltage and 1 nA probe current. High-resolution SEM images were obtained using an extra-lens Everhart-Thornley secondary electron detector. The pictures were acquired at an accelerating voltage of 5–10 kV and probe current of 200 pA. Total reflection X-ray fluorescence (TXRF) was applied to determine Gd concentration in Gd@C_82_EDA and Gd@C_82_EDA-ERL solutions. TXRF measurements were conducted utilizing the S4 T-STAR spectrometer, manufactured by Bruker AXS Microanalysis (Berlin, Germany). The instrument is equipped with a 50 W Mo target X-ray tube, a multilayer monochromator, and an SSD detector. The X-ray tube operated at 50 kV and 1000 µA. The measurements were carried out in an ambient air atmosphere, with a counting time of 1000 s, with Y utilized as an internal standard. The relaxometric properties of Gd@C_82_EDA and Gd@C_82_EDA-ERL were assessed by measuring longitudinal and transverse relaxation times. This evaluation was conducted using a Magritek Spisolve 60 MHz benchtop spectrometer device with a static magnetic field of 1 T. Each sample was diluted in Milli-Q water and measured at four different concentrations (between 0.1 and 1 mm). The r_1_ and r_2_ values (factors used to evaluate the efficiency of a sample as a contrast agent) were obtained as the slope resulting from the linear fit of the 1/T1/2 (s-1) relaxation time versus the metal concentration (mM). Cryo-EM was conducted on copper grids (Lacey carbon, EMS LC200-Cu) which were plasma-cleaned (Gatan Solarus, USA) for 30 s. Then, 3.5 μL of fullerene solution in DI water (c = 0.5 mg/mL) was applied to the grid, blotted for 2 s, and plunge-frozen using the Thermo Scientific Vitrobot (Mark IV). The grids were stored in liquid nitrogen until imaging, then clipped in autogrids (Thermo Scientific, USA) and placed into the cassette for loading onto the Titan Krios 3Gi (Thermo Scientific, USA). The grids were imaged using EPU (Thermo Scientific, USA).

### 2.3. Synthesis

The extended spectral data of synthesized small molecules and fullerene nanomaterials can be found in Supporting Information.

#### 2.3.1. Synthesis of 2-azidoacetic acid

We modified the procedure of Dyke for an experimental process [30]. First, we dissolved 1.76 g (12.7 mmol) of 2-bromoacetic acid in 20 mL of distilled water and cooled it to 0 °C. Then, we dissolved 1.46 g (22.5 mmol) of sodium azide (NaN₃) in 5 mL of water and added it dropwise over 10 min. Following a 15-min interval, the ice bath was removed, and the resulting reaction mixture was continuously stirred at ambient temperature for 12 hours. The reaction mixture was acidified gradually using a 2 M HCl solution until the pH level reached 1. Then, the resulting aqueous solution underwent extraction with Et_2_O which was performed thrice with 40 mL each time. The combined organic layers were subsequently treated with magnesium sulfate (MgSO₄) and filtrated. The final product was obtained as a colorless oil, with a yield of 45% (0.65 g).

#### 2.3.2. Synthesis of (2-(4-(3-((6,7-bis(2-methoxyethoxy)quinazolin-4-yl)amino)phenyl)-1 h–1,2,3-triazol-1-yl)acetic acid, ERL-COOH)

To prepare the desired product, 400 mg (1.02 mol) of erlotinib hydrochloride and 205 mg (1.78 mol) of 2-azidoacetic acid were dissolved in 20 mL of water with the addition of 5 mL of *tert*-butanol. After that, 25.4 mg (101.67 mmol) of copper sulfate and 20.14 mg (101.67 mmol) of sodium ascorbate were added to the solution. The resulting mixture was heated to 60 °C and stirred for 48 h. The reaction was considered complete when the erlotinib signal was no longer observed on the TLC plate. The solvent was then lyophilized, and the resulting yellow powder was purified on the column chromatography (final product R_f_ = 0.16, DCM : MeOH, 50:1, *v/v*). The white solid of the final product was collected with a 91% yield (462 mg, with a melting point of 218 °C).

#### 2.3.3. Synthesis of water-soluble aminofullerenes Gd@C_82_(EDA)_8_

30 mg (26.29 μmol) of Gd@C_82_ was dissolved in 20 mL of EDA (0.3 mol). The solution was then suspended in an ultrasonic bath for 15 min and then left to stir for 48 h at room temperature. The amine was evaporated, and the remaining brown solid was collected. To improve the solubility of aminofullerene, the transformation to hydrochloride was performed as follows: Gd@C_82_(EDA)_8_ was dissolved in 10 mL of water and 3 mL of 1 M HCl. After 1 h of stirring at room temperature, the solvent was evaporated. The product was dissolved in deionized water, purified using centrifugal membranes with 1k cutoffs, and lyophilized. The chemical composition was confirmed through elemental analysis and spectroscopic characterization (FT-IR, XPS, TXRF).

#### 2.3.4. C_60_(BUT)_3_ and C_70_(BUT)_3_

30 mg of pristine C_60_ (41.76 μmol) or C_70_ (35.71 μmol) was dissolved in 10 mL of BUT (99.5 mmol) and suspended using an ultrasonic bath for 15 min. The solution was then left to stir at room temperature for 48 h. The amine was evaporated, and the remaining brown solid was collected. To increase the solubility, the aminofullerene was transformed into hydrochloride. This was done by dissolving the obtained aminofullerene in 10 mL of water and 3 mL of 1 M HCl. After stirring at room temperature for 1 h, the solvent was evaporated. The product was then dissolved in deionized water and purified using centrifugal membranes with 1K cutoffs.

#### 2.3.5. General procedure for conjugation of aminofullerenes with ERL-COOH

Total of 30 mg of the desired aminofullerene (-NH_2_ form) Gd@C_82_EDA (24.78 μmol) or C_70_BUT (27.25 μmol) were dissolved in 20 mL of water and 5 mL of DMSO in the presence of NHS (one equivalent) and EDCI (one equivalent). Then, one equivalent of ERL-COOH was added, and the mixture was stirred at room temperature for 24 h. After 24 h, the solution was purified using centrifugal membranes with 1K cutoffs to remove any unreacted small molecular impurities. The aminofullerene synthesis was followed by hydrochloride formation to enhance the solubility in water and medium.

### 2.4. Determination of Gd concentration using the TXRF technique

To conduct TXRF analysis, we took 50 µL of previously sonicated suspensions of Gd@C_82_EDA or Gd@C_82_EDA-ERL and placed them inside a 2-mL Eppendorf tube. We then added 50 µL of 10 mg L^−1^ Y solution (internal standard), and 400 µL of 1% Triton-X-100 (surface active agent). The sample was then vortexed for 5 min and sonicated for 15 min. This ensured that the sample was evenly dispersed and homogeneous. Finally, 10 μL of suspension was carefully pipetted onto a siliconized quartz reflector and dried at 80 °C using a heating plate. The obtained results are summarized in Table S2 and Figure S14 shows the TXRF spectrum of the measured gadofullerenes.

### 2.5. Cell culture conditions

The PAN02 murine pancreatic carcinoma cell line was obtained from NCI-Frederick Cancer Research Facility, while the AsPC-1 human pancreas adenocarcinoma cell line and PANC-1 human pancreas ductal adenocarcinoma cell line were obtained from Sigma Aldrich. The NHDF normal human dermal fibroblast cell line was acquired from PromoCell. The PANC-1 cells were cultured in Dulbecco’s modified Eagle’s medium (DMEM) which was supplemented with 10% heat-inactivated fetal bovine serum (FBS) from Merck. The DMEM for the NHDF was supplemented with 15% non-inactivated FBS. PAN02 and AsPC-1 cells were cultured in Roswell Park Memorial Institute (RPMI) 1640 which was supplemented with 10% heat-inactivated FBS from Merck. Each complete medium included a combination of two antibiotics, penicillin, and streptomycin (1% *v/v*; Gibco). All the cell lines were maintained at 37 °C in a 5% CO_2_ humidified atmosphere.

### 2.6. Cytotoxicity measurements

The cells were seeded in 96-well Nunc plates with a density of 5,000 cells/well (cancer cells) or 4,000 cells/well (normal cells), and incubated under standard conditions at 37° C for 24 h. The cells were then incubated for 72 h with varying concentrations of the tested compounds. The next step was adding DMEM without phenol red, along with CellTiter 96®AQueous One Solution-MTS (Promega), to each well, and incubating for either 1 or 3 h at 37 °C. The samples’ optical densities were then measured at 490 nm using a multi-plate reader (Varioskan LUX, Thermo Scientific). The results obtained were compared to the control and calculated as inhibitory concentration (IC_50_) values using GraphPad Prism 9. Each specific compound underwent triplicate testing in a single experiment, and each experiment was replicated three or four times.

### 2.7. Cell cycle assay

The PAN02 and AsPC-1 cells were seeded in a 3 cm Petri dish at a density of 200,000 cells/dish and incubated at a temperature of 37 °C. After 24 h, we replaced the medium and exposed each cell line to C_60_BUT, C_70_BUT-ERL, and Gd@C_82_EDA-ERL nanomaterials. After another 24 h of incubation, we conducted assays employing the Muse™ Cell Cycle Kit (Millipore) following the supplier’s instructions. In brief, the cells were harvested, cold PBS-washed, and centrifuged. The cells were then fixed in ice-cold 70% ethanol and stored at −20 °C overnight and the subsequent day involved washing the cells with cold PBS, centrifuging, and resuspending them in Muse™ Cell Cycle Reagent. After a 30 min incubation at room temperature in the dark, the samples were analyzed for cellular subpopulation values across different cell cycle phases using a Muse Cell Analyzer (Millipore). This experimental protocol was repeated a minimum of three times for reliable results.

### 2.8. Apoptosis assay

The cells were treated the same as mentioned in the section above. After 48 h of treatment, assays were conducted using the FITC Annexin V Apoptosis Detection kit with 7-AAD (Bio-Legend), following the manufacturer’s instructions. The cells were harvested, PBS-washed, and centrifuged. Next, the cells were reconstituted in Annexin V Binding Buffer and incubated for 15 min at room temperature in the dark with FITC Annexin V and 7-AAD Viability Staining Solution. Following staining, the samples were assessed for live, early, and late apoptotic cells using the Muse Cell Analyzer. Each experiment was replicated a minimum of three times to obtain reliable outcomes.

### 2.9. Western blot measurements

The PAN02 and AsPC-1 cells were seeded in 3 cm Petri dishes at a density of 500,000 cells per well and incubated under standard conditions for 24 h. Next, the cells were incubated with freshly prepared solutions of tested C_60_BUT, C_70_BUT-ERL, and Gd@C_82_EDA-ERL nanomaterials for one day. After that, the pancreatic cells were detached by trypsinization, collected into Eppendorf tubes, and centrifuged. The cell pellets were resuspended in a RIPA buffer containing Halt Protease Inhibitor Cocktail, Halt Phosphatase Inhibitor Cocktail along with 0.5 M EDTA (all from Thermo Scientific) and lysed on ice for 20 min. The obtained lysates were sonicated and centrifuged for 10 min at 4 °C. The supernatants were transferred to new tubes and used in further studies. To determine the protein concentration, a Micro BCA™ Protein Assay Kit (Thermo Scientific) was used as per the manufacturer’s instructions. 20 μg of the proteins were electrophoresed on SDS-Page gels and transferred onto nitrocellulose membranes. The membranes were blocked in 5% nonfat milk prepared in TTBS (containing 0.1% Tween-20) for 1 h. After this time, the membranes were incubated with specific primary antibodies at 1:1000 dilution. The following primary antibodies were used: EGFR, phospho-EGFR (Tyr1068), mTOR, phospho-mTOR (Ser2448), Akt, phospho-Akt (Ser473), IDH1, PTEN, PI3K p85, Ras, Src, p27^Kip1^, cyclin E1, PARP, GAPDH, and vinculin. The incubation was carried out overnight at 4 °C. The following day, the membranes were washed in TTBS and incubated with horseradish peroxidase (HRP)-conjugated secondary antibodies for 1 h at room temperature. Then, the membranes were washed in TTBS and incubated with a SuperSignal West Pico Chemiluminescent Substrate (Thermo Scientific). The chemiluminescence signals were captured using a ChemiDoc® XRS+ System (BioRad). The experiments were repeated at least four to five times and the densitometric analysis was performed using ImageJ software (Wayne Rasband, National Institutes of Health, USA).

### 2.10. Transmission electron microscopy and *in vivo* toxicology

Ten adult specimens of *D. melanogaster* of variation y, w (I); 43.4, y +, (II) obtained from the Vienna Resource Center were cultured in the laboratories of the University of Silesia in Katowice using Nutri-Fly® Grape Agar. The animals were divided into two groups: FC, the control group, which were cultured under laboratory conditions and fed ad libitum with yeast paste devoid of gadofullerenes for one week, and F1W, which were cultured under laboratory conditions and fed ad libitum with yeast paste supplemented with gadofullerene Gd@C_82_EDA-ERL (c = 1 mg/mL) for one week. The precise laboratory culture of *D. melanogaster* was previously described in our paper [31]. The total number of adult specimens (both males and females) was 10. The insects were anesthetized with CO_2_. The midgut (middle region of the digestive system) after isolation, was fixed, washed, dehydrated, and embedded in epoxy resin (Epoxy Embedding Medium Kit; Sigma) according to standard protocols [32,33]. Ultrathin sections were cut using Leica EM UC7 RT (70 nm) and, after contrasting, were examined using a Hitachi H500 transmission electron microscope at 75 kV.

## 3. Results

### 3.1. Synthesis and characterization

**Figure 2.**
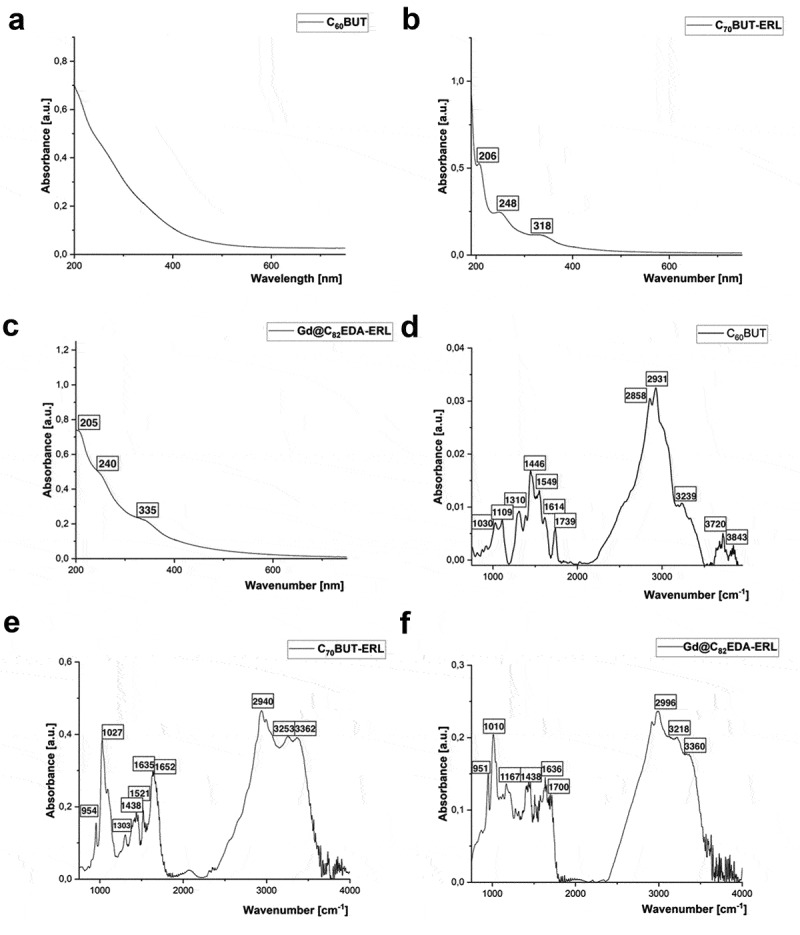
Spectroscopic characterization of synthesized aminofullerenes: UV-VIS spectra of obtained fullerene nanomaterials (a–c), and FT-IR spectra of synthesized compounds (d–f).

Three aminofullerenes, C_60_BUT, C_70_BUT, and Gd@C_82_EDA, were synthesized through a straightforward liquid-liquid reaction at room temperature. Their molecular structure was characterized using FT-IR, UV-VIS, ^13^C-NMR, and XPS. The number of amines attached to fullerene cores was estimated using elemental analysis. For 1,4-diaminebutane fullerene derivatives, the nitrogen-to-carbon ratio (*N/C*) was used to calculate the amount of added amine units as three. For Gd@C_82_, the calculation confirmed the formation of octakis adduct with EDA Gd@C_82_(EDA)_8_ (Table S1, Supporting Information). The ^13^C-NMR spectra of two BUT derivatives of fullerenes showed four characteristic signals of alkyl chains around 70 and 20 ppm as well as one weak signal of [60]fullerene core around 146 ppm and two signals of [70]fullerene core around 150 and 147 ppm, respectively (Figures S4-S5, Supporting Information). The FT-IR analysis of the synthesized aminofullerenes (C_60_BUT, C_70_BUT, and Gd@C_82_EDA) showed notable absorption bands between 1750 to 1400 cm^−1^ corresponding to N – H bending vibrations of primary amines (Figures 2(d–f)). Moreover, the vibrational bands of N-H (3500–3800 cm^−1^) and C-H (2800–3000 cm^−1^) of the carbon cage were observed for all water-soluble fullerene derivatives. If we consider the obtained aminofullerenes as primary amine hydrochlorides with terminal -NH_3_ units, they all presented broad intense -NH_3_^+^ stretching envelopes. In general, for primary amine salts, this envelope falls from in the spectrum region between 3300 to 2800 cm^−1^. Additionally, C – N stretch signals presented between 1250–1020 cm^−1^ were characteristic of aliphatic amines and were observed in all synthesized aminofullerenes.

The synthesized erlotinib carboxylic acid (ERL-COOH)) was examined further using ^13^C-NMR spectroscopy which revealed the absence of two characteristic triple bond signals (around 82 and 77 ppm) and the formation of a triazole ring (two signals around 146 and 132 ppm) with an additional strong signal of carboxylic acid around 170 ppm (Figure S2). The high-resolution mass spectrometry confirmed the presence of a molecular peak of ERL-COOH at 493.1829 Da in negative polarization [M-H]- (calculated mass: 493.1841 Da).

In the analysis of nanoconjugation products by FT-IR spectroscopy, we observed the characteristic signals of secondary amide functional groups, which were only present in the expected products. For the compound C_70_EDA-ERL, we observed the C=O stretch of our secondary amide (Figure 2(e)) at 1641 cm^−1^ close to the signals of N – H bending vibrations of primary amines (1635 cm^−1^). The companion peak shown in Figure 2(e) around 1521 cm^−1^ was associated with the in-plane N-H bend of the secondary amide group, which is normally found from 1570 to 1510 cm^−1^. Additionally, for compound Gd@C_82_EDA-ERL, we observed strong and intense bands at 1700 cm^−l^ as stretching vibrations of the carbonyl group present in the secondary amide, whereas a band near 1530 cm^−1^ is characteristic of the in-plane N−H bends of the secondary amide group (Figure 2(f)). Unfortunately, the presence of a triazole ring (weak signals around 3100 cm^−1^) could not be confirmed by an analysis of the infrared spectrum of synthesized nanoconjugates because of the dominating signals of N-H and C-H functional groups in that region. Furthermore, the unequivocal evidence for the attachment of the erlotinib derivative to selected aminofullerenes was the comparison of their electronic spectra. Unmodified fullerene nanomaterials possess a characteristic UV-VIS spectrum with exponential decay and without a distinct absorption maximum (Figures 2(a)). In the case of both nanoconjugates, signals at 245 and 320 nm corresponding to the erlotinib fragment can be observed (Figure 2(b,c)).

**Figure 3.**
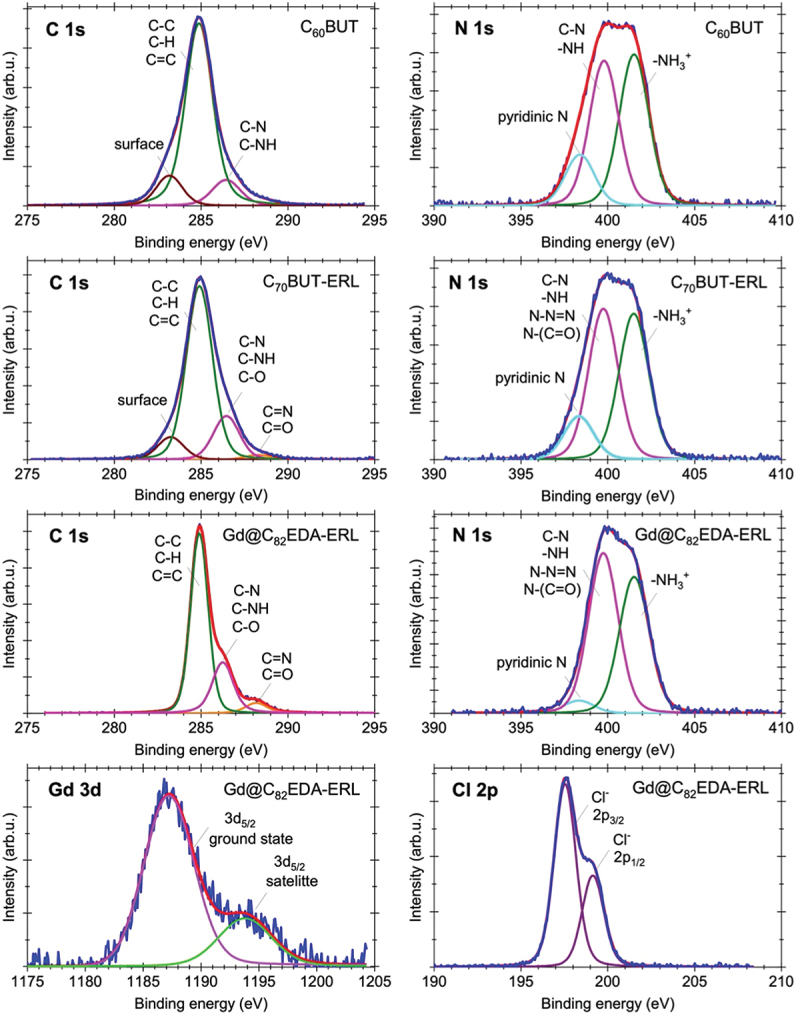
The XPS C 1s, N 1s, Gd 3d5/2, and Cl 2p lines of C_60_BUT, C_70_BUT-ERL, and Gd@C_82_EDA-ERL fullerene derivatives.

High-resolution XPS was used to further investigate the electronic structure of C_60_BUT, C_70_BUT-ERL, and Gd@C_82_EDA-ERL fullerenes. The analysis of the chemical composition included the identification of elements, chemical bonding, and calculations of atomic concentrations. Trace contamination of silicon was observed in addition to the lines corresponding to the elements of the compound. These traces can be considered as resulting from synthetic impurities or sample preparation. Figure 3 shows the XPS spectra of the C 1s, N 1s, Cl 2p, and Gd 3d5/2 regions together with the corresponding deconvolution. The C 1s peak was decomposed into three or four lines, representing carbon atoms in various functional groups. The line with a binding energy of 284.9 eV indicates the presence of unoxidized graphitic carbon in the C-C, C=C, or C-H bonds [34]. The chemical state with a binding energy of 286.4 eV is associated with groups containing C-N and C-NH bonds. This line exhibits higher intensity for C_70_BUT-ERL and Gd@C_82_EDA-ERL fullerenes compared to the C_60_BUT sample and may be additionally ascribed to the groups containing carbon-oxygen bonds (C-O). Furthermore, for these two samples, carbonyl C=O and C=N groups were identified by the third line at 288.4 eV [35]. The N 1s peak can be deconvoluted into three components. The first chemical state, occurring at the energy of 398.4 eV, is assigned to basic nitrogen of the pyridine type [36]. The second line observed at a binding energy of 399.8 eV is associated with the C-N and -NH bonds (free amino groups) in all fullerenes, as well as with the *N*-(C=O) bond and nitrogen atoms of the triazole ring in the C_70_BUT-ERL and Gd@C_82_EDA-ERL samples [37]. The third line localized at 401.5 eV is attributed to the partially protonated amino group -NH_3_^+^. This group is expected to be shifted to a higher binding energy compared to the original nitrogen due to the creation of a positive center [38]. The analysis of the N 1s spectrum depicted in Figure 3 revealed that the number of -NH_3_^+^ bonds calculated using the peak area ratio relative to the C-N, -NH bonds was comparable for the C_60_BUT fullerene and decreased slightly in relation to C-N, -NH, N-N=N, *N*-(C=O) bonds for the C_70_BUT-ERL and Gd@C_82_EDA-ERL fullerenes. The Cl 2p peak could be fitted to one chlorine atom environment with binding energy values of 197.5 eV and 199.1 eV for two lines splitting by spin-orbit interaction and corresponding to chloride anions presented in the fullerene nanomaterials [39]. Moreover, the XPS spectrum of the Gd 3d5/2 line for Gd@C_82_EDA-ERL, located at 1187.0 eV, proves that fullerene C_82_ contains Gd inside the carbon core [40].

Interestingly the EDS results aligned with the elemental composition, including C, N, Gd, and O, providing additional confirmation of successful conjugation with ERL-COOH. Subsequent investigations aimed to understand the dispersion behavior of the obtained CNs in aqueous solutions, recognizing their paramount importance for future biological evaluations. Our findings revealed that C_60_BUT nanomaterials demonstrated the propensity to form two distinct types of aggregates, with diameters of approximately 110 nm and significantly larger aggregates around 4400 nm (constituting about 1% of the aggregates, Figure S12). In contrast, C_70_BUT-ERL samples predominantly formed smaller aggregates approximately 6.5 nm in diameter (representing about 5% of the aggregates), with the main fraction being around 140 nm (Figure S13). Moreover, the gadolinium-incorporated fullerenes exhibited the capability to form singular aggregate types with an average diameter of 200 nm. Importantly, for all synthesized aminofullerenes, the polydispersity index remained below 0.3, indicating a narrow size distribution.

### 3.2. Cytotoxicity assay

The potential of the synthesized fullerene nanomaterials for inhibition of the proliferation of three pancreatic cancer cell lines (PANC-1, PAN02, and AsPC-1) and a normal fibroblast cell line (NHDF) is presented in Figure 6(a). IC_50_ value for C_60_BUT incubated with PANC-1 cell line equals 16.88 µM. This fullerene showed the highest effectiveness also against AsPC-1, with an IC_50_ value of 31.51 µM. For the mouse PAN02 cell line, the IC_50_ value was 45.56 µM. IC_50_ values for C_70_BUT-ERL fullerene for PAN02 and AsPC-1 cell lines amounted to 65–66 µM, while for PANC-1 was above 300 µM. Gadofullerene nanomaterial showed activity between 193.52 and above 425 µM. The ligand ERL-COOH exhibited the weakest activity with the IC_50_ values from 234.82 to 574.90 µM. Moreover, the nanomaterials tested showed much weaker activity against NHDF normal cells. The calculated IC_50_ values for C_60_BUT and C_70_BUT-ERL on fibroblast cells were 158.55 µM and 247.21 µM, respectively. While gadofullerene, ligand ERL-COOH, and erlotinib showed no activity.

### 3.3. Cell cycle inhibition

The impact of fullerene nanomaterials on the cell cycle progression in PAN02 and AsPC-1 cells was determined by flow cytometry. The results are presented in Figure 6(b), and representative histograms for each nanomaterial in Figure S16. For the PAN02 cell line incubated with C_60_BUT and Gd@C_82_EDA-ERL, a statistically significant increase (about 8–11% compared to the control) in the cell population in the G0/G1 phase was observed. For the AsPC-1 cell line an increase in the cell population in the G0/G1 phase was recorded in all cases.

### 3.4. Apoptosis induction

The potential of the tested materials to generate apoptosis in PAN02 and AsPC-1 was determined by Annexin V-FITC and 7-ADD staining using flow cytometry. The results are shown in Figure 6(c), with representative histograms depicted in Figure S17. In the PAN02 cells, the biggest increase (from 7.27% in control to 16.40%) in the population of the apoptotic cells was detected for Gd@C_82_EDA-ERL at 320 µM. Greater increases were observed for the AsPC-1 cell line, with the biggest for C_70_BUT-ERL at 67 µM, namely from 11% in control to 44.24%.

### 3.5. Western blot analysis

Basal levels of proteins associated with the EGFR signaling pathway were determinated by the Western Blot technique (Fig. S15). A high level of EGFR was detected in AsPC-1 and PANC-1 cell lines, with a high level of phospho-EGFR (Y1068) only for AsPC-1, in contrast to PAN02 cells with no expression of both proteins. An increased level of Akt and phospho-Akt (S473) was registered only for PANC-1 and PAN02 cell lines. In turn elevated level of IDH1 protein was observed for both PAN02 and AsPC-1 cell lines. Also, a high level of PI3K was detected in PAN02 cells.

Analysis of protein expression after incubation of pancreatic cells with the tested nanomaterials (Figure 7 and S18) indicates an increased level of p27^Kip1^ in PAN02 cells treated by the C_60_BUT and Gd@C_82_EDA-ERL. For AsPC-1 cells, enhancement of the p27^Kip1^ protein was also registered for the C_60_BUT at 35 µM (5-fold increase), C_70_BUT-ERL at 17 µM and Gd@C_82_EDA-ERL at 160 µM (3-fold increase). All of the tested nanomaterials reduced the cyclin E1 activity by at least 2-fold in the AsPC-1 cell line. PARP cleavage was registered on the PAN02 cell line after treatment with Gd@C_82_EDA-ERL, while it was not detected in the AsPC-1 cell line. A downregulation in mTOR protein levels after treatment with C_60_BUT and C_70_BUT-ERL was observed in PAN02 cells. Moreover, both nanomaterials reduced p-mTOR activation. In contrast incubation of both cell lines with gadofullerene caused an increase in mTOR and p-mTOR levels. The level of EGFR protein was markedly lower after incubation with C_60_BUT (45 µM) and C_70_BUT-ERL in mouse pancreatic cancer. P-EGFR expression was also reduced in PAN02 cells by all of the tested fullerenes, except C_70_BUT-ERL (13 µM). For the second AsPC-1 cell line tested, the decrease in the concentration of these proteins was much lower. A reduction in the protein levels of Akt and phosphorylated Akt on serine 473 was observed after incubation with all of the tested fullerenes for both cell lines. Namely, C_60_BUT resulted in more than 3-fold, while C_70_BUT-ERL and Gd@C_82_EDA-ERL induced a 6.5-fold decrease in p-Akt levels in the AsPC-1 cell line. In the case of PAN02 cell line, C_60_BUT and C_70_BUT-ERL induced over 1.5-fold decrease in p-Akt activation, while gadofullerene reduced expression by almost 4-fold. Reduced levels of PI3K p85 were detected in the PAN02 cells, while in the AsPC-1 it was not detected. Downregulation of PTEN was detected in PAN02 after incubation with C_60_BUT and C_70_BUT-ERL, and in AsPC-1 only for gadofullerene. IDH1 protein was decreased in PAN02 after treatment with C_60_BUT and gadofullerene. Ras protein level in AsPC-1 cells treated with all of the tested fullerenes was reduced, except for C_60_BUT (35 µM).

## 4. Discussion

The reaction of fullerene nanomaterials with amines can lead to the formation of structurally diverse compounds, depending on factors such as the reaction conditions, light illumination, type of amine, or catalyst [41–43]. For instance, primary amines have been shown to undergo reactions with the fullerene core, yielding straightforward multi-addition derivatives. Nonetheless, even minor alterations in the amine structure can facilitate the synthesis of various fulleropyrrolines. This phenomenon was observed in the reaction of [60]fullerene with β-substituted ethylamine derivatives in the presence of oxygen [44]. Moreover, it has been observed that a secondary amine can also undergo a single-step, multi-addition process with [60]fullerene under photochemical aerobic conditions, resulting in the formation of tetra(amino)fullerene epoxides [45]. However, numerous studies have shown that SET (single electron transfer)-facilitated excited-state addition reactions involving tertiary alkyl and aromatic amines with C_60_ are inefficient [46]. Aminofullerenes obtained from these reactions are extensively used in biomedical applications, such as drug delivery vehicles, photosensitizers for the inactivation of bacteria and viruses, and carriers of nucleic acids, including siRNA [47–49].

In the course of developing fullerene-derived inhibitors of EGFR protein, we decided to modify the structure of erlotinib by incorporating a carboxyl moiety within the triple bond fragment, taking into account the preservation of biological activity, in particular the interactions with the molecular target. Previously, Satpati and coworkers proposed modifications of the terminal alkyne function in the erlotinib structure to form ^177^Lu-labeled erlotinib conjugates, without decreasing its efficacy as a tyrosine kinase inhibitor [50]. Building on our previous research on fluorescent fullerene triazoles, we used a copper-catalyzed azide-alkyne cycloaddition between the terminal alkyne fragment in the phenyl ring of erlotinib and 2-azidoacetic acid, to form erlotinib disubstituted 1,2,3-triazole derivative with carboxyl functional group (shown in Figure S1, alongside spectral characterization depicted in Figures S1-S3 within the Supporting Information) [51].

The process of conjugating water-soluble aminofullerenes (C_60_BUT, C_70_BUT, and Gd@C_82_EDA) with erlotinib carboxylic acid was performed using one equivalent of water-soluble carbodiimide EDCI and *N*-hydroxysuccinimide. This ensured the nanoconjugation of one equivalent of the erlotinib derivative. This procedure was based on our previous work, where saturation of all available amines resulted in loss of solubility as well as stability of the obtained fullerene nanoconjugates. To purify the final aminofullerene nanoconjugates, we used membrane dialysis with dedicated membranes (molecular cutoffs 3k) to remove all small molecular impurities. The success of the ERL-COOH conjugation was confirmed using FT-IR and UV-VIS analysis. Unfortunately, we were not able to synthesize and further test the erlotinib conjugate with C_60_BUT due to its extremely low water-solubility and stability of formed nanoconjugate. However, we decided to assess and describe its anticancer activity and model of action due to its very promising biological activity.

**Figure 4.**
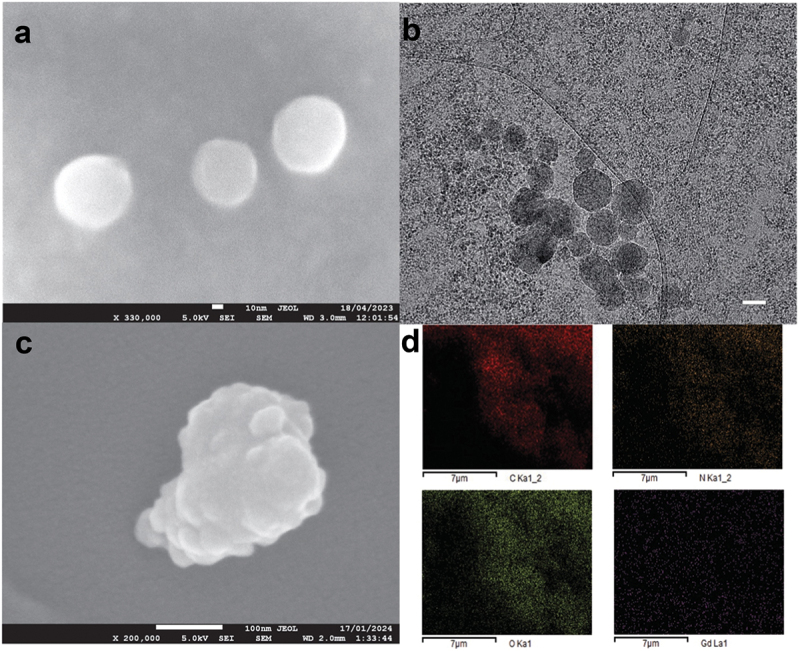
The SEM (a–c), cryo-EM (b, scale bar 20 nm), and EDS images (d) of synthesized aminofullerenes. The spherical aggregates of C_60_BUT (a) and C_70_BUT-ERL (b) as well as fluffy-type aggregates of Gd@C_82_EDA-ERL (c) were observed.

The morphological characteristics of chemically modified aminofullerenes and their nanoconjugates with ERL-COOH were systematically investigated using SEM and cryo-EM. Our observations revealed the formation of spherical aggregates in engineered CNs, particularly in C_60_BUT (as depicted in Figure 4(a)) and the C_70_EDA-ERL nanoconjugate (illustrated in Figure 4(b)). For larger fullerene cores such as gadolinium-containing fullerene Gd@C_82_EDA-ERL, we detected the formation of fluffy aggregates and confirmed their derivatization through nanoscale elemental compositional analysis conducted *via* EDS mapping in the SEM, as depicted in Figure 4(d).

**Figure 5.**
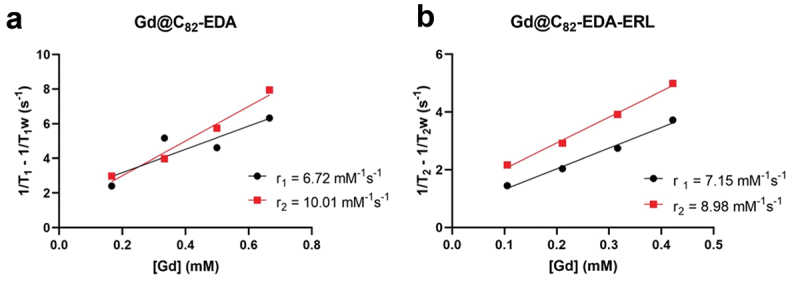
The r_1_ and r_2_ relaxivities of synthesized gadofullerenes.

The use of water-soluble nanomaterials as theranostic agents has increased significantly. One area that has received particular attention is the use of engineered gadofullerenes (Gd@C_82_ and Gd_3_N@C_80_) and iron oxide nanoparticles [52,53]. Gadofullerene nanoparticles are highly effective in enhancing MRI relaxivity by one to two orders of magnitude, offering a substantial improvement over conventional method. They protect the encapsulated gadolinium atoms from metabolic degradation, which effectively reduces the risk of gadolinium leakage. Recent advances in synthetic methods, such as hydroxylation, Bingel-Hirsch, and Prato reactions, have enabled the formulation of water-soluble gadofullerenes that are highly biocompatible [21,54]. To assess the MRI-contrast properties of synthesized gadofullerene Gd@C_82_EDA-ERL, we first measured the concentration of gadolinium in the sample using the TXRF method (Table S2). We then measured the T1 and T2 relaxation times of the synthesized gadofullerenes and calculated r_1_ and r_2_ relaxivities (Figure 5). It was found that the obtained nanotheranostic molecule Gd@C_82_EDA-ERL had a relaxivity parameter r_1_ of 7.15 mM^−1^s^−1^ (at 1 T), which was lower than that of previously described gadofullerenes containing β-alanine units (13 mM^−1^s^−1^). However, the r_1_ values of the clinically used Gd chelates are much lower, generally about 3–6 mM^−1^s^−1^ under similar measurement conditions. Moreover, the described gadofullerenes contain conjugated small molecules with biological activity, making them truly theranostic nanomaterials.

### 4.1. Biological studies of aminofullerenes and their nanoconjugates with erlotinib derivative

**Figure 6.**
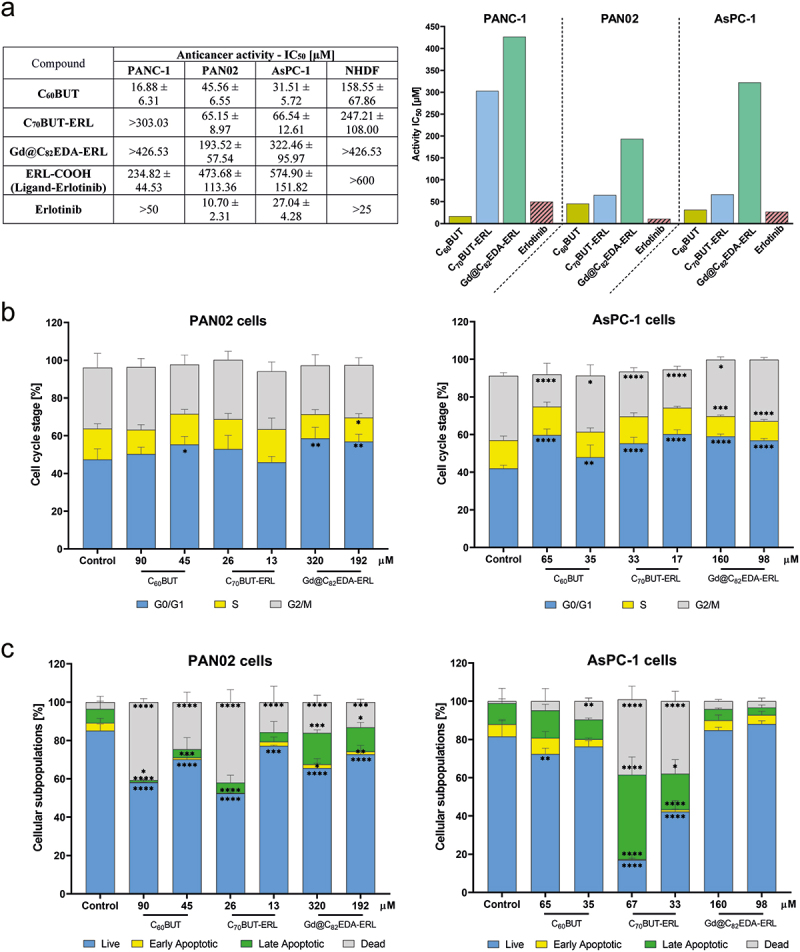
The antiproliferative activity of tested nanomaterials (C_60_BUT, C_70_BUT-ERL, and Gd@C_82_EDA-ERL), ligand ERL-COOH and erlotinib against pancreatic cells: PANC-1, PAN02, AsPC-1, and NHDF (a). The impact of tested nanomaterials on the progression of cell cycle (b) and apoptosis induction (c) in PAN02 and AsPC-1 cells. Statistical analysis was performed using one-way ANOVA with Dunnett’s post hoc test: *p < 0.05, **p < 0.01, ***p < 0.001, ****p < 0.0001 compared with the untreated cells (control).

Biological evaluation was carried out on synthesized fullerene nanomaterials to determine their antiproliferative activity on three pancreatic cancer cell lines, namely PANC-1, PAN02, and AsPC-1. The results are presented in Figure 6(a). Overall, C_60_BUT showed the highest efficacy against all tested cancer cells. In particular, this fullerene was most effective against human PANC-1 cells, with an IC_50_ value of 16.88 µM. Interestingly, the FDA-approved EGFR inhibitor, erlotinib, was not effective (IC_50_ >50 µM). It is important to note that PANC-1 cells were resistant to other tested nanomaterials, C_70_BUT-ERL and Gd@C_82_EDA-ERL. For the second tested human pancreatic cancer cell line, AsPC-1, the antiproliferative activity of C_60_BUT was similar to erlotinib, with IC_50_ values of 31.51 µM for fullerene and 27.04 µM, respectively. However, for mouse PAN02 cells, erlotinib was more effective (IC_50_ = 10.7 µM). Although C_60_BUT had more than 4-fold lower efficacy than erlotinib, it still displayed high activity (IC_50_ = 45.56 µM). Moreover, the C_70_BUT-ERL fullerene, with conjugated erlotinib, exhibited good antiproliferative activity against PAN02 and AsPC-1 cells (IC_50_ = 65–66 µM). On the other hand, the gadofullerene nanomaterial primarily inhibited cell proliferation of the murine pancreatic cells (IC_50_ = 193.52 µM), while being more resistant against both human pancreatic cancers. It is worth noting that the ligand ERL-COOH, which is erlotinib with triazole group with carboxyl function attached to phenyl ring (instead of ethynyl group), subsequently linked to C_70_ and Gd@C_82_ aminofullerenes, showed negligible activity, especially in PAN02 and AsPC-1 cell lines. It is worth noting that our nanomaterials had a high specificity against pancreatic cancer cells.

We conducted further studies on the molecular mechanism of action of the three nanomaterials- C_60_BUT, C_70_BUT-ERL, and Gd@C_82_EDA-ERL – to understand their impact on cell cycle progression, apoptosis induction, and regulation of EGFR cell signaling pathway. For this, we used two pancreatic cancer cell lines – PAN02 and AsPC-1—based on the activity of tested nanomaterials and the landscape of basal protein expression levels. Our Western Blot experiments confirmed large diversity in the expression proteins associated with the EGFR signaling pathway in tested pancreatic cancer cells (Figure S15). Namely, the AsPC-1 cells show a high level of EGFR and phospho-EGFR (Y1068) expression, while their expression is relatively deficient in the PAN02 cells. Of note, the observation of high EGFR receptor expression in AsPC-1 cells has been confirmed in reports [55,56]. The complete opposite pattern is observed in the basal expression levels of Akt and phospho-Akt (S473) in these pancreatic cell lines. Both PAN02 and AsPC-1 cells display elevated expression of the IDH1 protein (compared to PANC-1), which is one of the components of the tricarboxylic acid cycle and is involved in cellular metabolism, proliferation, and energy production [57]. Interestingly, we detected a very high level of PI3K expression in PAN02 cells, while it was absent in the AsPC-1 cell line. In light of this diversification, the behavior of the tested nanomaterials in cells is crucial for understanding the interactions of signaling networks, which may have implications for therapeutic efficacy and further potential clinical relevance.

**Figure 7.**
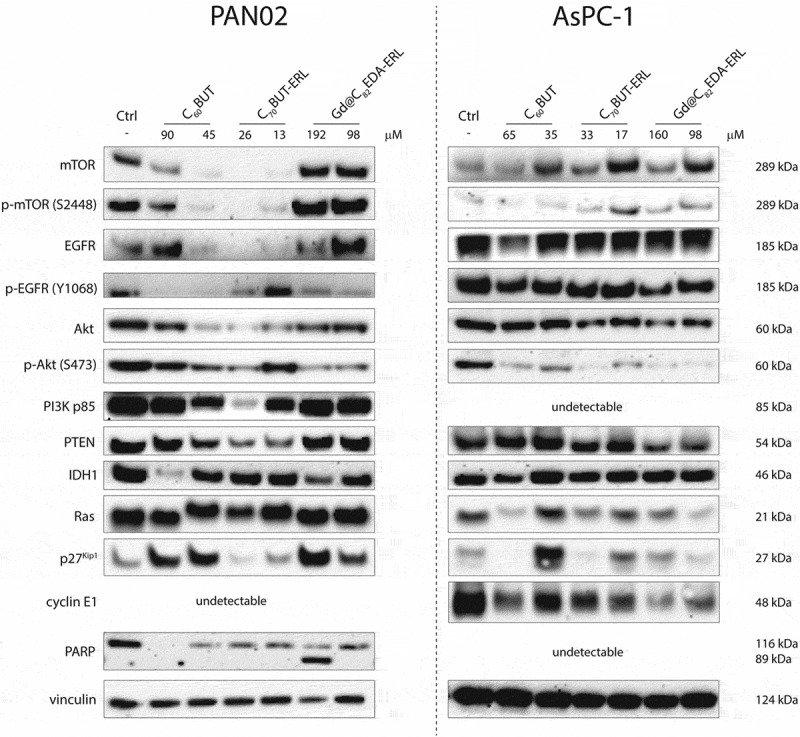
The level of protein expression after exposure to fullerene nanomaterials: C_60_BUT, C_70_BUT-ERL, and Gd@C_82_EDA-ERL in PAN02 and AsPC-1. Densitometric analyses of these results as well as uncropped and unmodified blots were provided in the Supporting Information.

The impact of fullerenes on the cell cycle progression and apoptosis induction in PAN02 and AsPC-1 cells was determined using flow cytometry. The charts are presented in Figures 6(b,c), and representative histograms for each nanomaterial in Figures S16-S17. Our results showed that the tested fullerenes arrested the cell cycle in the G0/G1 phase (Figure 6(b)). In PAN02 cells, the C_60_BUT and gadofullerene induced a statistically significant increase in the cell population in the G0/G1 phase by about 8%–11% compared to the control (untreated cells), with a simultaneous decrease in the number of cells in S and G2/M phases. Interestingly, a greater degree of inhibition was observed in AsPC-1 cells. Treatment with C_60_BUT at 65 µM caused a significant increase in the percentage of cells in the G0/G1 phase from 41.93% (in control) to 59.73%. Similar effects were observed in AsPC-1 cells after administration of C_70_BUT-ERL at 17 µM and Gd@C_82_EDA-ERL at 160 µM. It is worth noting that our previous studies on the anticancer activity of glycofullerenes did not indicate their effect on cell cycle inhibition, despite their ability to inhibit tyrosine kinase and redox imbalance [58]. Therefore, we determined the expression of p27^Kip1^ and cyclin E1 in cells after exposure to tested fullerenes. Both proteins are closely related to the ability of the cell cycle to enter from the G0/G1 to the S phase. The activity of the cyclin E1-cdk2 complex can be inhibited by p27^Kip1^, which is a negative regulator of the cell cycle and blocks the transition to the DNA synthesis phase [59]. As depicted in Figure 7, the C_60_BUT induced a more than 2-fold increase in the expression of p27^Kip1^ in PAN02 cells. In addition, this protein was almost 3.5-fold upregulated after exposure to Gd@C_82_EDA-ERL.

After incubation with tested nanomaterials, the p27^Kip1^ protein level was found to be higher in AsPC-1. The C_60_BUT at 35 µM induced a marked 5-fold increase in the expression of cyclin-dependent kinase inhibitor, while the C_70_BUT-ERL at 17 µM and Gd@C_82_EDA-ERL at 160 µM caused a more than 3-fold enhancement of p27 production (Figures 7 and S18). Since there were more prominent changes in cell cycle inhibition in AsPC-1 cells, we also examined the compounds’ effect on cyclin E1 expression. As expected, all of the tested fullerenes reduced the cyclin E1 activity by at least 2-fold. These results are consistent with flow cytometry measurements and prove that aminofullerenes have a significant effect on arresting the cell cycle in the G0/G1 phase.

The studies on apoptosis were performed using Annexin V-FITC and 7-ADD staining to detect early and late apoptotic cell populations. Annexin V is an important protein marker of the onset of apoptotic cell death that binds with phosphatidylserine, which is a phospholipid that changes its location from the inner side of the cell membrane to the outer surface side during the early phase of apoptosis. Thus, the cellular protein Annexin V conjugated with a fluorescence dye can bind to it. Our data indicate clear changes in live and dead populations in the PAN02 cells. However, the percentage of cells in the early and late apoptosis phases was quite low (Figure 6(c)). For example, the number of cells in the late apoptosis phase increased from 7.27% in control to 16.40% after treatment with Gd@C_82_EDA-ERL at 320 µM. A similar situation was observed in AsPC-1 cells, with one exception. C_70_BUT-ERL at 67 µM induced a significant increase in the population of late apoptotic cells from 11% in control to 44.24%.

Protein analysis unequivocally indicates that after treatment with Gd@C_82_EDA-ERL, cleaved PARP was registered on the PAN02 cell line (Figure 7). The results coincide with flow cytometer data where an increase in apoptotic cells was observed in the PAN02 line despite moderate levels, in contrast to the AsPC-1 cell line where (except in one case) the increase was negligible and PARP cleavage was not registered in Western Blot. Nevertheless, it seems that apoptosis is not the primary type of cell death induced by tested fullerenes. Instead, autophagic cell death may be more relevant. In our previous reports, glycofullerenes and glycine-derived [60]fullerene could activate autophagosome formation and induce autophagy through the regulation of LC3 and p62 [58,60]. Zhang et al. also described the role of the Nano-C_60_ in inducing ROS-dependent autophagy and doxorubicin chemosensitization in Hela cells by regulating Atg5 expression [61]. The activation of autophagy flux through up-regulation of cathepsin D, which caused cyclin D1 degradation and G0/G1 cell cycle arrest, has been reported for C_70_-EDA [26]. Interestingly, the functionalization of the surface of fullerenes may determine their special properties, as well as the induction of the cell death mechanism through apoptosis or autophagy [62].

The role of autophagy induction in cancer treatment is still controversial. However, recent reports have suggested that this therapeutic approach may be effective as it positively regulates and promotes the immune response [63]. One of the crucial complexes that regulate the survival signaling pathway and block autophagy stimulation is mTOR. To investigate this, we analyzed the expression of this protein and the activation of p-mTOR after treatment with tested nanomaterials at the cellular level (Figure 7). We observed a downregulation in mTOR protein levels after treatment with C_60_BUT and C_70_BUT-ERL in PAN02 cells. Additionally, both fullerenes induced a significant reduction in p-mTOR activation. C_60_BUT (45 µM) and C_70_BUT-ERL (26 µM) showed a 4-fold and 7.6-fold decrease in phosphorylation of mTOR at the serine 2448 site, respectively. In AsPC-1 cells, we also detected reduced levels of p-mTOR protein after exposure to C_60_BUT at higher concentrations. Surprisingly, both protein levels increased after exposure to gadofullerene in PAN02 and AsPC-1 cell lines.

We proceeded to analyze the impact of C_60_BUT, C_70_BUT-ERL, and Gd@C_82_EDA-ERL on the regulation of the EGFR protein and its downstream pathway targets such as Akt, p-Akt, PI3K, and PTEN. The activation of EGFR/Akt signaling can mediate cellular responses and influence mTOR expression to control metabolism, proliferation, cell size, survival, and motility [64]. The results are presented in Figure 7, and the densitometric analyses of the tested proteins are depicted in Figure S18. It is noteworthy that the effect of the tested nanomaterials on the total level of EGFR and its activation was much higher in mouse pancreatic cancer than in the case of AsPC-1. In PAN02 cells, the total level of EGFR protein was markedly lower after incubation with C_60_BUT and C_70_BUT-ERL. In the second cell line tested, there were less noticeable changes only after administration of C_60_BUT at a concentration of 65 µM. The activation of EGFR by tyrosine phosphorylation at the 1068 site was significantly inhibited by all of the tested fullerenes in PAN02 cells. The highest inhibitory effect was observed for C_60_BUT at both tested concentrations (10-fold decrease). C_70_BUT-ERL (26 µM) and gadofullerene (98 µM) caused a 2-fold reduction in p-EGFR protein expression.

Once EGFR is activated, the main effectors of its signaling cascade are Akt and PI3K proteins. As expected, a reduction in the protein levels of Akt and phosphorylated Akt on serine 473 was observed after incubation with fullerenes. C_60_BUT and C_70_BUT-ERL induced over 1.5-fold decrease in p-Akt activation, while gadofullerene reduced expression by almost 4-fold.

Interestingly, fullerenes had a greater impact on p-Akt levels in AsPC-1 cells. Specifically, C_60_BUT resulted in more than 3-fold, while C_70_BUT-ERL and Gd@C_82_EDA-ERL induced at least a 6.5-fold decrease in p-Akt levels. In addition, slightly reduced levels of PI3K p85 were detected in PAN02 cells. Notably, the PI3K activation leads to the transformation of phosphatidylinositol 4,5-bisphosphate (PIP2) to phosphatidylinositol 3,4,5-trisphosphate (PIP3), which mediates the direct launch of Akt signaling [65]. There were also interesting changes observed for the PTEN protein, which acts as an antagonist of PI3K activity. The use of C_60_BUT and C_70_BUT-ERL in PAN02 led to the downregulation of PTEN.

In contrast, gadofullerene caused a reduction of PTEN levels in AsPC-1. This reduction should have allowed Akt signaling to be maintained. However, it was noted that due to the multilevel impact of fullerenes and their ability to interact with and inhibit the entire EGFR/Akt/mTOR signaling pathway, it may induce its suppression.

Moreover, IDH1 and Ras proteins play pivotal roles in cellular metabolism and the regulation of proliferation. In PAN02, we observed a significant decrease in IDH1 expression following treatment with C_60_BUT and gadofullerene. Notably, the Ras protein, alongside other proteins within the RAF-MEK-ERK-MAPK signaling pathway, serves as a critical mediator of the EGFR response. The interaction of ERK and MAPK proteins with various substrates initiates cellular responses regulating cell growth, proliferation, differentiation, and apoptosis evasion [66]. Additionally, Ras protein can bind to PI3K via the p110 subunit, influencing the activation of cellular events associated with the Akt pathway [67]. Our analyses revealed that the tested fullerenes can modulate the Ras protein levels in AsPC-1 cells. Specifically, C_60_BUT and gadofullerene caused a more than 2.5-fold reduction in Ras expression, while C_70_BUT-ERL decreased protein levels to a slightly lesser extent. However, significant changes were not observed in mouse pancreatic cells. Comparing the complexity of the regulation of cellular molecules and pathways, as well as the rate of cellular responses, despite the lack of clear changes in the AsPC-1 cell line, it appears that fullerenes can suppress EGFR signaling pathway activity, as evidenced by substantial changes in p-Akt and Ras protein activity.

**Figure 8.**
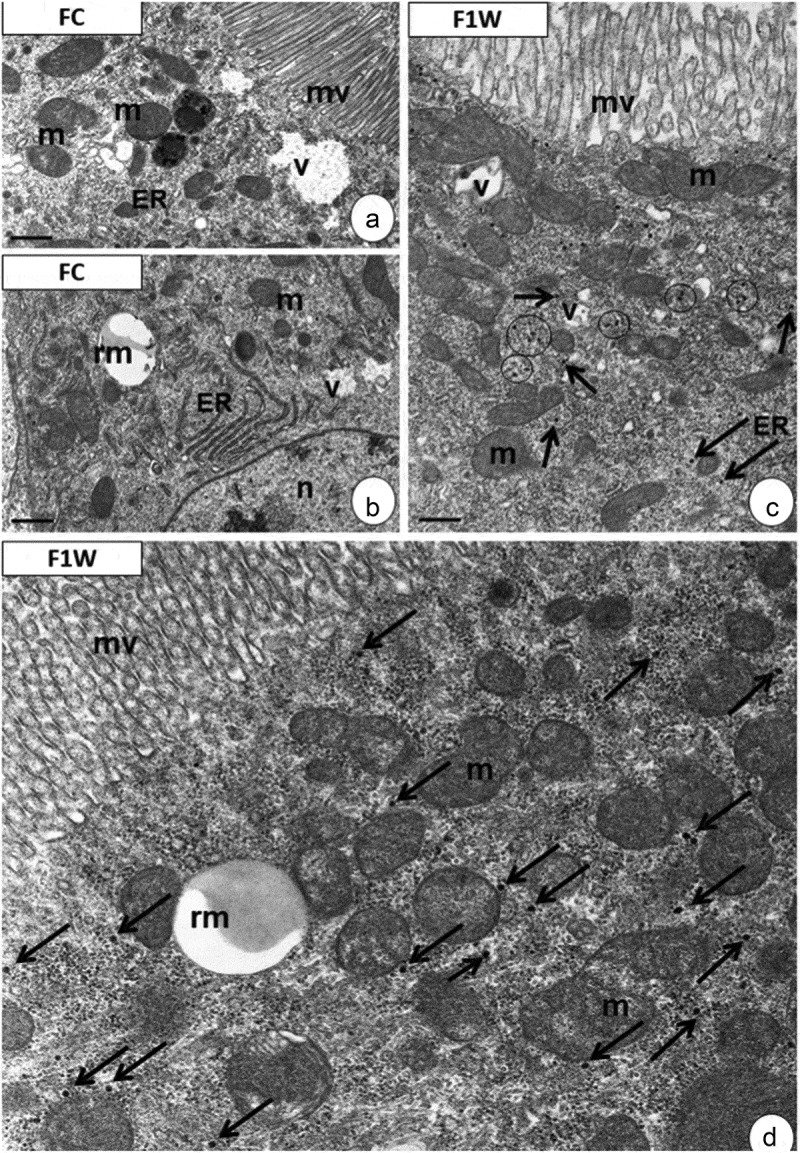
Midgut of *D. melanogaster*. (a-b) FC group (control group). Apical (a) and perinuclear (b) cytoplasm of midgut enterocytes. (c-d) F1W experimental group. Specimens fed with fullerenes for 1 week. Mitochondria (m), microvilli (mv), nucleus (n), cisterns of endoplasmic reticulum (ER), vesicles (v), reserve material (rm), electron-dense granules (black arrows). (a) Scale bar = 1.2 µm. (b) Scale bar = 1.2 µm. (c) Scale bar = 1 µm. (d) Scale bar = 0.8 µm.

In further toxicological research at the *in vivo* level, we chose to study the gadofullerene Gd@C_82_EDA-ERL nanomaterial due to its potential for translational studies (Figure 8). We used the fruit fly model (*Drosophila melanogaster*) for this purpose, as it has a short lifespan and is an excellent choice for genetic and cell biology research [68]. However, there are limited studies on how water-soluble fullerene nanoparticles interact with fruit flies [31]. We observed no changes in cell ultrastructure in the midgut epithelium of *D. melanogaster* individuals (males and females) compared to the control group (Figures 8(a,b)). All organelles had the correct structure. After one week of the experiment, TEM revealed the presence of electron-dense, homogenous granules distributed throughout the cytoplasm (Figures 8(c,d)). These granules were not observed in the cytoplasm of midgut enterocytes of control specimens.

## 5. Conclusions

In summary, we have successfully created versatile fullerene nanomaterials and their conjugates with erlotinib derivative to improve the treatment accuracy for pancreatic cancer. Our innovative aminofullerenes C_60_BUT, C_70_BUT-ERL, and Gd@C_82_EDA-ERL formed spherical or fluffy-type aggregates in water. The structures were confirmed using several spectroscopic techniques such as FT-IR, NMR, UV-VIS, XPS, and high-resolution ESI MS. We also conducted detailed biological analysis of fullerene nanomaterials of *in* *vitro* and *in* *vivo* models. Cytotoxicity studies on pancreatic cancer cell lines revealed high anticancer activity of C_60_BUT and C_70_BUT-ERL. The C_60_BUT nanomaterial showed similar biological activity to erlotinib (IC_50_ in the range of 16–45 µM). Further cellular studies on PAN02 and AsPC-1 cell lines showed the ability of the tested fullerene nanomaterials to arrest the cell cycle in the G0/G1 phase, which was also confirmed by changes in the expression of p27 and cyclin E1 proteins. Moreover, induction of cell death by autophagy for C_60_BUT and C_70_BUT-ERL and apoptosis for Gd@C_82_EDA-ERL were indicated. Most importantly, we revealed the impact of the tested fullerene nanomaterials on the inhibition of the EGFR signaling pathway by reducing the expression of p-EGFR, p-Akt, PI3K, and Ras proteins. Finally, we confirmed the lack of toxicity of Gd@C_82_EDA-ERL on *Drosophila melanogaster*.

## Supplementary Material

Supplemental Material

Supplemental Material

## Article highlights

- The successful synthesis of three aminofullerenes was demonstreted (C_60_BUT, C_70_BUT, and Gd@C_82_EDA) as well as their two nanoconjugates (C_70_BUT-ERL, Gd@C_82_EDA-ERL). They were obtained *via* straightforward direct amination of buckyball cores, followed by further nanonocjugation and structurally confirmed by multiple spectroscopy techniques (FT-IR, UV-Vis, NMR and XPS).
- It was revealed that C_60_BUT exhibits significant anti-cancer efficacy against several pancreatic cancer cell lines (PANC-1, AsPC-1, PAN02), with IC_50_ values comparable to, or better than, the FDA-approved inhibitor erlotinib.
- The unique dispersion behaviors of these novel aminofullerenes in aqueous solutions should be higlighted, forming well-defined nanoscale aggregates suitable for biomedical applications.
- It was shown that the fullerene derivatives arrest the cell cycle in G0/G1 phase in both PAN02 and AsPC-1 cell lines, driven by elevated p27 and reduced cyclin E1 expression.
- It was demonstrated that C_60_BUT and C_70_BUT-ERL favor autophagy induction, while Gd@C_82_EDA-ERL triggers apoptosis, underscoring distinct modes of cell death.
- The suppression of EGFR signaling (p-EGFR, p-Akt, PI3K, Ras) in pancreatic cancer cells was confirmed, providing a mechanistic explanation for the robust anticancer effects.
- The safety profile of Gd@C_82_EDA-ERL was verified *in vivo* using the *Drosophila melanogaster* model, showing no detectable toxicity or detrimental ultrastructural changes in midgut epithelium.
- The translational potential of gadofullerene-based theranostics should be highlighted, demonstrating favorable MRI relaxivity while simultaneously carrying biologically active compounds.
