# Supplementary material for: Aminofullerenes as targeted inhibitors of EGFR: from pancreatic cancer inhibitors to Drosophila m. Toxicology
Source: Nanomedicine (Lond). 2025 Feb 7;20(6):585–601. doi: 10.1080/17435889.2025.2461985 (PMC11881853; doi:10.1080/17435889.2025.2461985)
Supplement: Supplemental Material [file INNM_A_2461985_SM9660.docx]

Aminofullerenes targeting EGFR: From pancreatic cancer inhibition to toxicology in Drosophila melanogaster

# Chemistry

- - *Spectral data of 2-azidoacetic acid*
  - *NMR spectrum of* ERL-COOH *(^1^H- and ^13^C-NMR)*
  - *HRMS spectrum of ERL-COOH*
  - *FT-IR spectra of synthesized aminofullerenes and ERL-COOH*
  - *UV-VIS spectra of synthesized aminofullerenes and ERL-COOH*
  - *DLS measurements of synthesized aminofullerenes*
  - *TXRF spectrum of gadofullerene Gd@C82EDA and Gd@C82EDA-ERL*
  - *Elemental analysis of synthesized aminofullerenes*
  - *The chemical composition of Gd@C82EDA and Gd@C82EDA-ERL determined with TXRF spectrometry.*

# Biology

- - *Basal protein level in tested pancreatic cells*
  - *Histograms from cell cycle studies*
  - *Histograms from apoptosis studies*
  - *Densitometric analysis of studied proteins after treatment with nanomaterials*
  - *Uncropped and unmodified blots*
  - *Spectral data of 2-azidoacetic acid*

**O**

**N**

**3**

**OH**

The compound was synthesized using modified procedure of Dyke^1^.

**^1^H-NMR (*500 MHz, CDCl3, ppm* ):** 8.39 (s, 1H, -COOH), 3.97 (s, 2H, CH2).

**^13^C-NMR (*126 MHz, CDCl3, ppm*):** 173.39 (C=O), 50.04 (CH2).

- ***Spectral data of ERL-COOH (2-(4-(3-((6,7-bis(2-methoxyethoxy)quinazolin-4- yl)amino)phenyl)-1****H****-1,2,3-triazol-1-yl)acetic acid)***

**^1^H-NMR (*500 MHz, CDCl3, ppm* ):** *δ 9.91 (s, 1H, N****H****), 8.47 (s, 1H, aryl* ***H****), 8.41 (s, 1H, aryl* ***H****),*

8.35 (t, J = 2.0 Hz, 1H, aryl **H**), 8.12 (s, 1H, aryl **H**), 7.95 (ddd, J = 8.0, 2.2, 1.0 Hz, 1H, aryl **H**),

7.54 (dt, J = 7.6, 1.4 Hz, 1H, aryl **H**), 7.41 (t, J = 7.9 Hz, 1H, aryl **H**), 7.21 (s, 1H, aryl **H**), 4.86

(s, 2H, -C**H**2), 4.38 – 4.33 (m, 2H, -C**H**2), 4.31 – 4.25 (m, 2H, -C**H**2), 3.81 – 3.71 (m, 4H, -C**H**2),

3.36 (d, J = 5.5 Hz, 6H, -C**H**3).

**^13^C-NMR (*126 MHz, CDCl3, ppm*):** *δ 169.54 (****C****OOH), 156.94 ( aryl* ***C****), 153.94 ( aryl* ***C****), 153.37*

(aryl **C**), 148.46 (aryl **C**), 147.37 (aryl **C**), 146.27 ( triazole ring **C**), 140.65 (aryl **C**), 131.85 (triazole ring **C**), 129.26 (aryl **C**), 123.02 (aryl **C**), 121.89 (aryl **C**), 120.44 (aryl **C**), 119.20 (aryl **C**), 109.60 (aryl **C**), 108.56 (aryl **C**), 104.14 (aryl **C**), 70.58 (d, J = 12.8 Hz, **C**H2), 68.95 (**C**H2),

68.46 (**C**H2), 58.82 (**C**H3), 49.02 (**C**H2).

**Melting point:** 217 °C

**HR-ESI MS (negative polarisation):** *Chemical Formula [M-H]^-^: C24H25N6O6; Exact Mass (calculated: 493.1841 Da); measured: 493.1829 Da.*


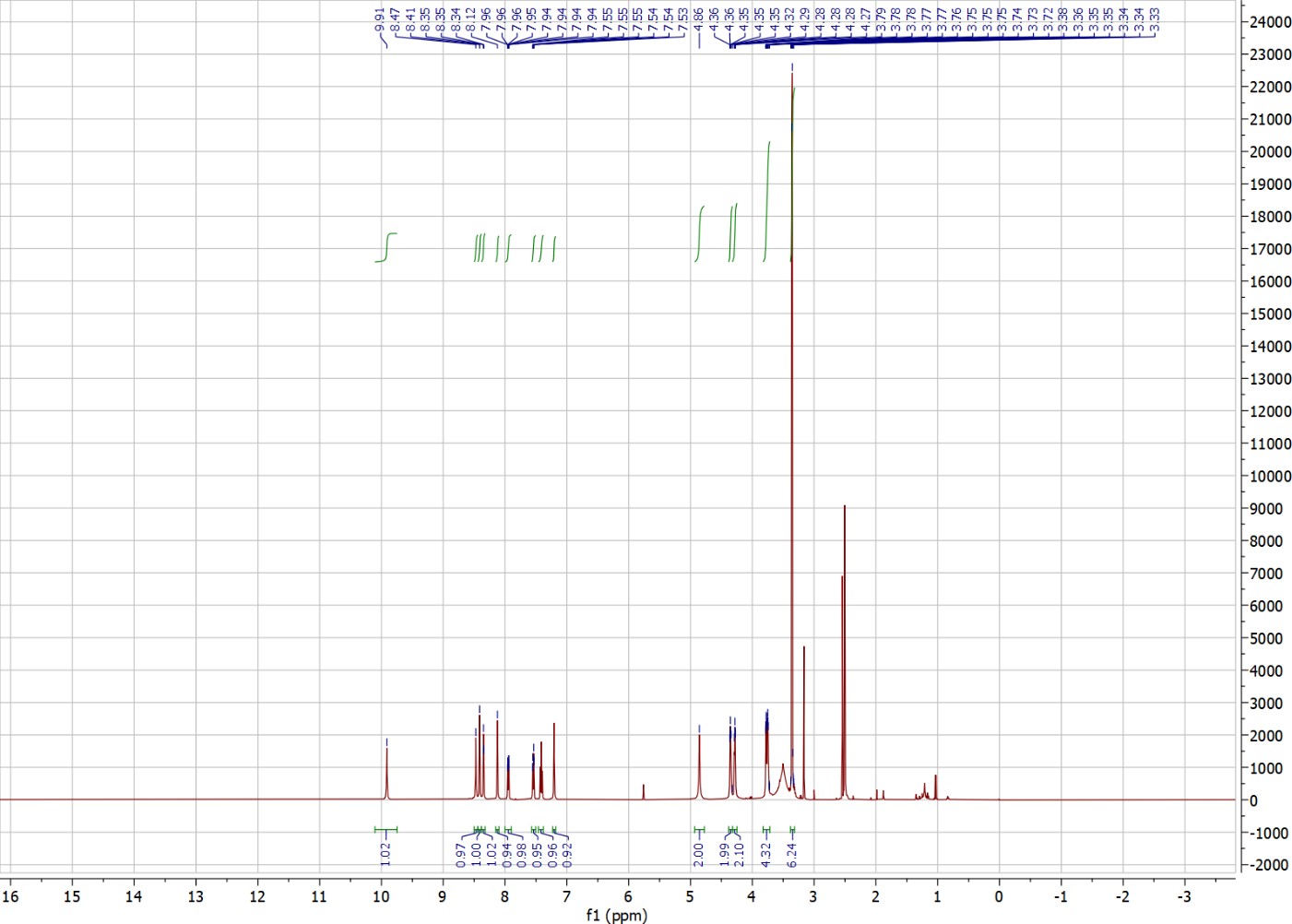


# Figure S1

^1^H-NMR of ERL-COOH.


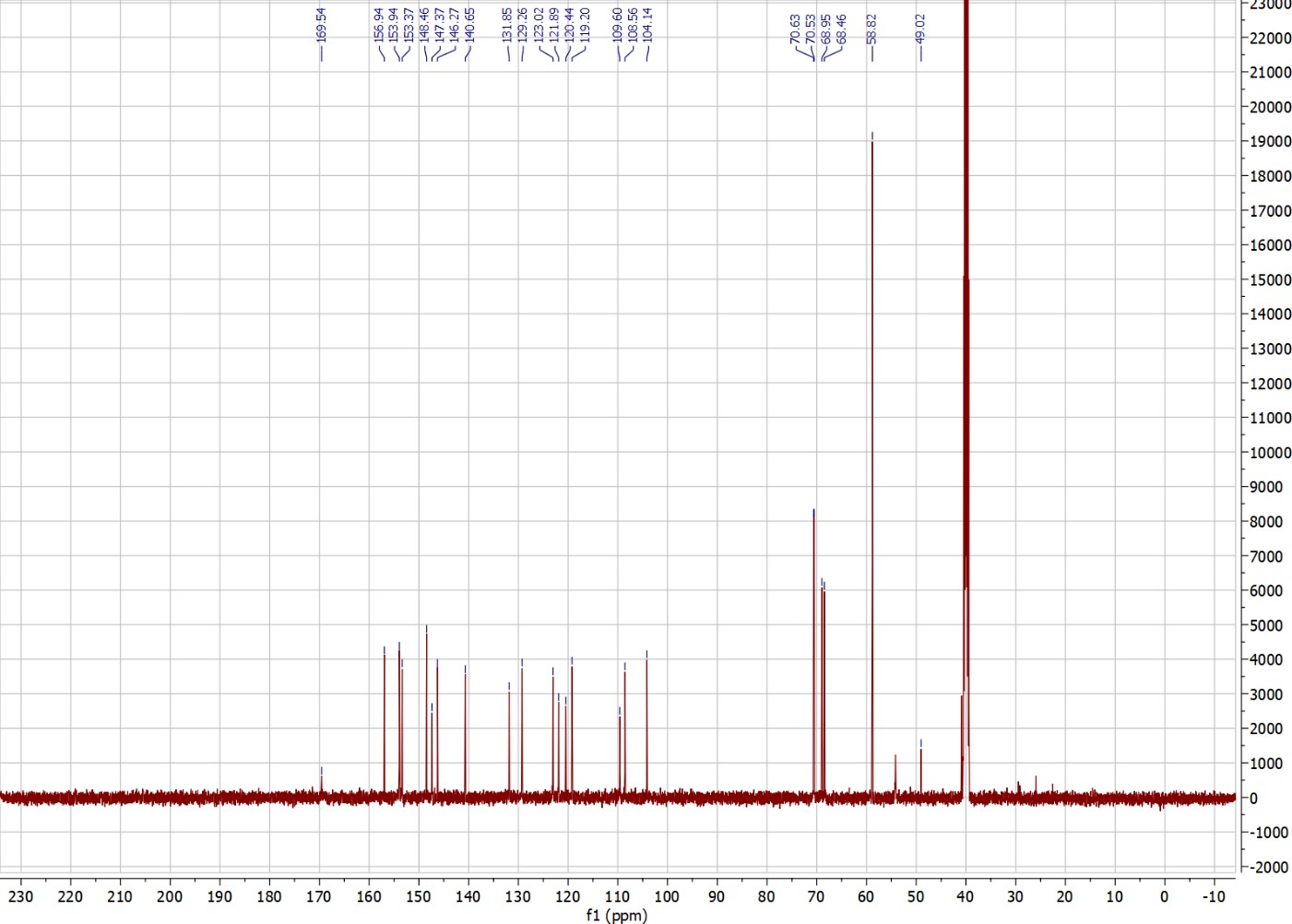


# Figure S2

^13^C-NMR of ERL-COOH


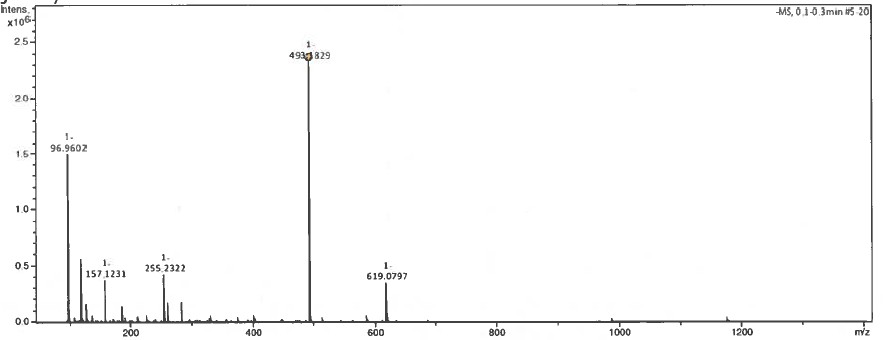

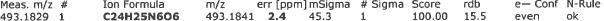


# Figure S3

High resolution ESI spectrum of compound ERL-COOH

38000

CVYSd$67TgS7G5VLKBKhdg.9323.lid C601,4BUT

13C

t-

t-

t-

t-

+ +

T r

+

t

+

36000

| + |  | + | + | + | + |  | + | 34000 |
| --- | --- | --- | --- | --- | --- | --- | --- | --- |
| + |  |  | + |  |  | I + |  | 32000 |
| + | + | | + | + | + | + | + | 30000 |
| + | t- | | + |  | t- | t- | + | 28000 |
| + | + | | + | + | + | + | + | 26000 |
| + | t- | | + | + | t- | t- | + | 24000 |
| -1- |  | | + |  |  | + |  | 22000 |
| + | + | | + | + | + | + | + | 20000 |
| t- |  | | + |  |  | + |  | 18000 |
| + | + | | + | + | + | + | + | 16000 |
| + | t- | | + |  | t- | t- | + | 14000 |
| + | + | | + |  | + | + | + | 12000 |
| + | + | | + | + | + | +- | + | 10000 |
| + |  | | + |  |  | + |  | 8000 |
| + | +- | | + | + | +- | +- | + | 6000 |
| + | t- | | + |  | t- | t- | + | 4000 |
| i | | | | | | ...,.,,.. .J. |  | 2000  -2000  -4000 |

230 220 210 200 190 180 170 160 150 140 130 120 110 100 90 80 70 60 50 40 30 20 10 ·10

11 (ppm)

# Figure S4

^13^C-NMR spectrum of C60BUT in D2O

240 230 220 210 200 190 180 170 160 150 140 130 120 110 100 90 80 70 60 50 40 30 20 10

11 (ppm)

170000

160000

| c0SkPJ$eQw+kCJmQ3nEWaw.474.fid  C701.4 BUT HCI +-  13C  I T | | |
| --- | --- | --- |
| +- +-  t- + t- t-  +- +- +- +- | |  |
| +- +- +-  + + +  t- t- t-  +- +- t- +-  + + +  + +  t- t- t-  +- +- +- +-  + + +  + +  t- t- t-  +- +- +- +- |  |  |
|  | | |

150000

140000

130000

120000

110000

100000

90000

80000

70000

60000

50000

40000

30000

20000

10000

-10000

# Figure S5

^13^C-NMR spectrum of C70BUT in D2O


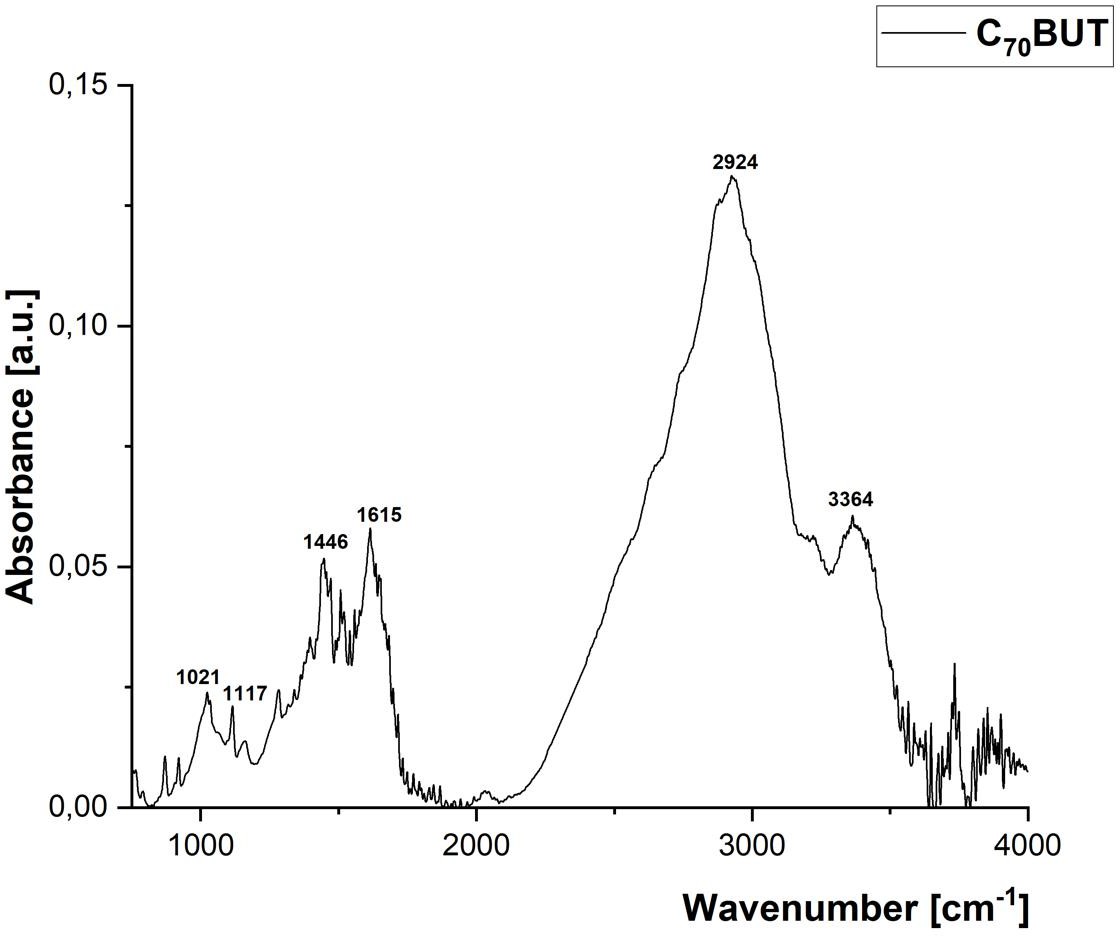


# Figure S6

FT-IR spectrum of fullerene nanomaterial C70BUT


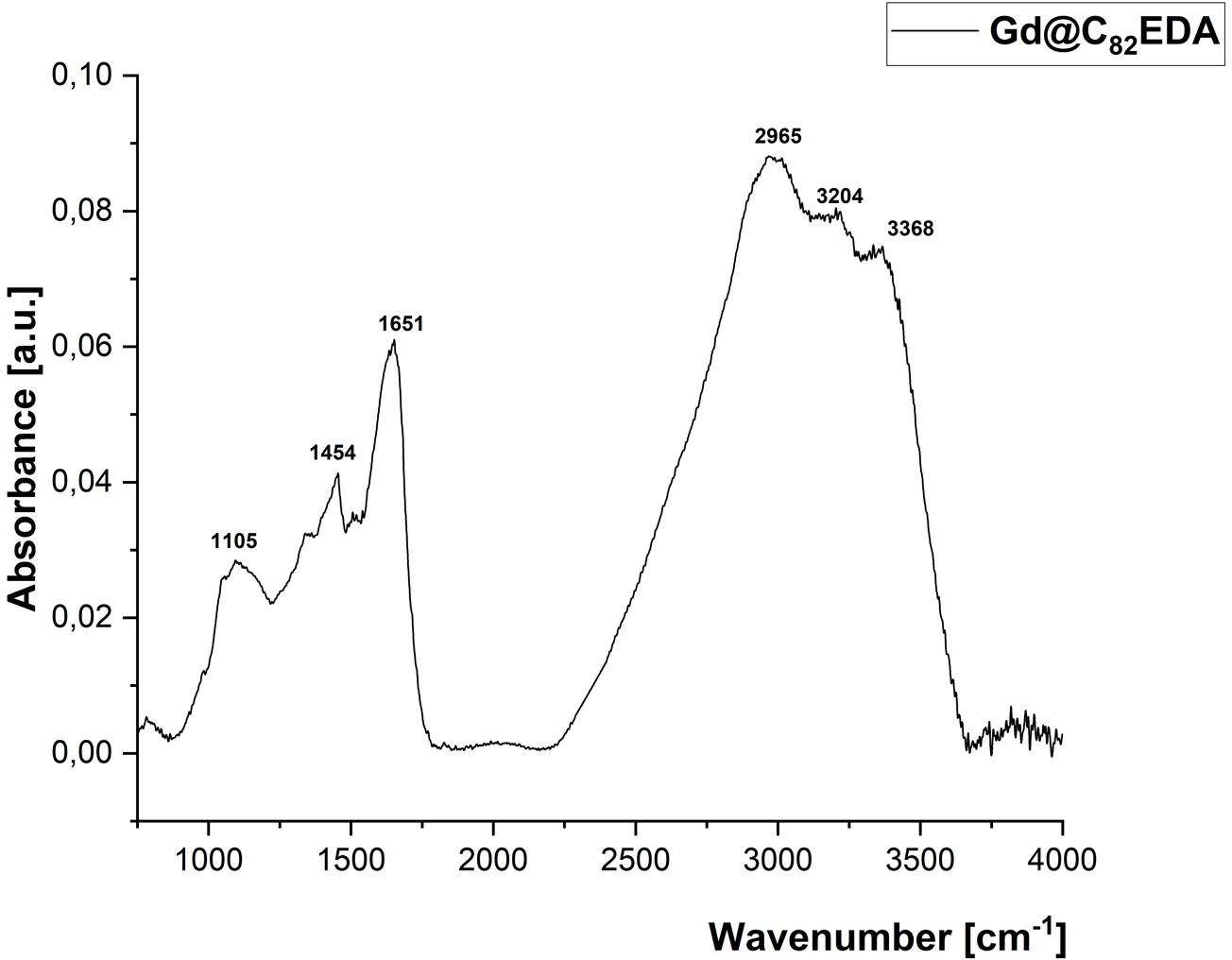


# Figure S7

FT-IR spectrum of fullerene nanomaterial Gd@C82EDA.


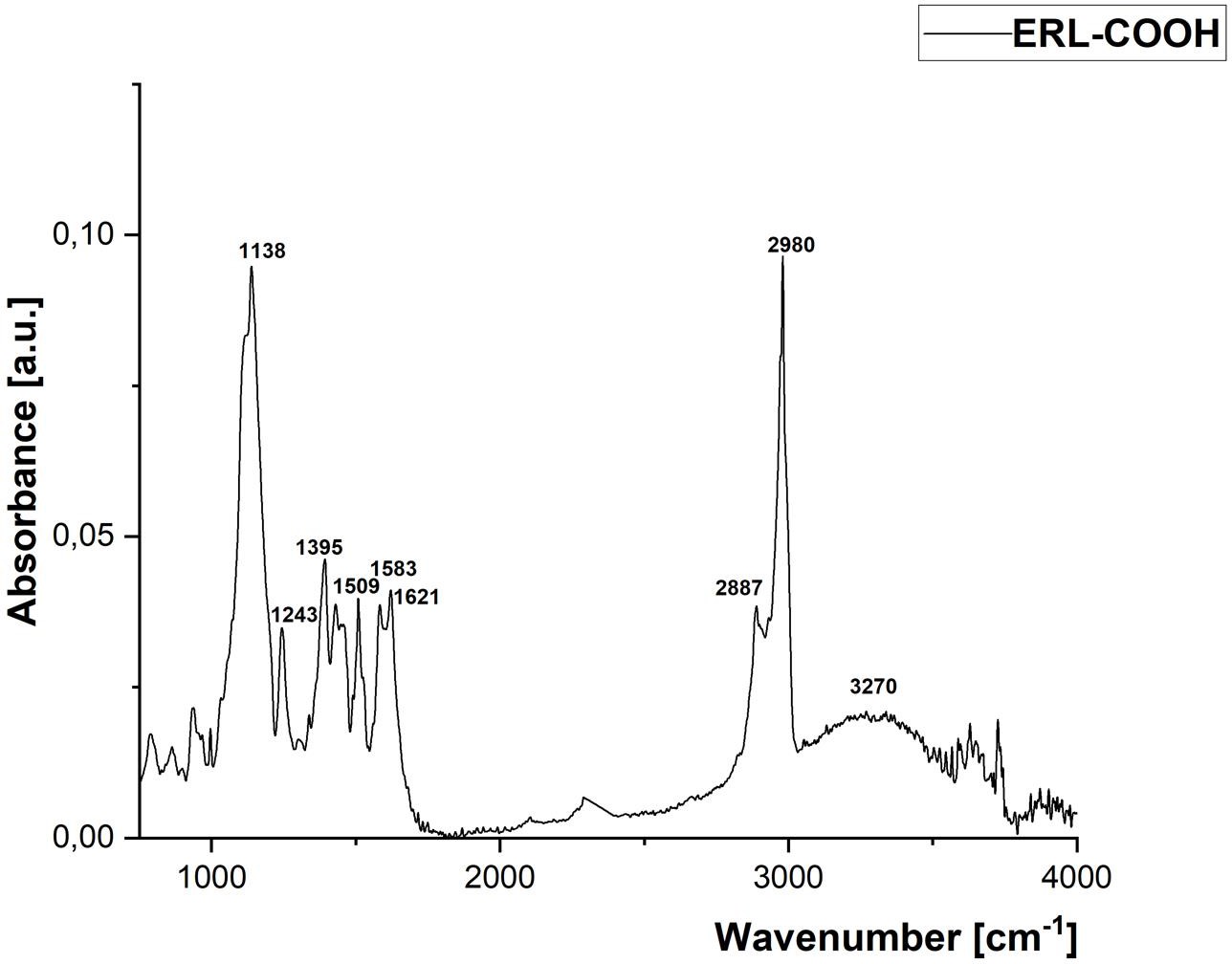


# Figure S8

FT-IR spectrum of ERL-COOH *[2-(4-(3-((6,7-bis(2-methoxyethoxy)quinazolin-4- yl)amino)phenyl)-1H-1,2,3-triazol-1-yl)acetic acid]*


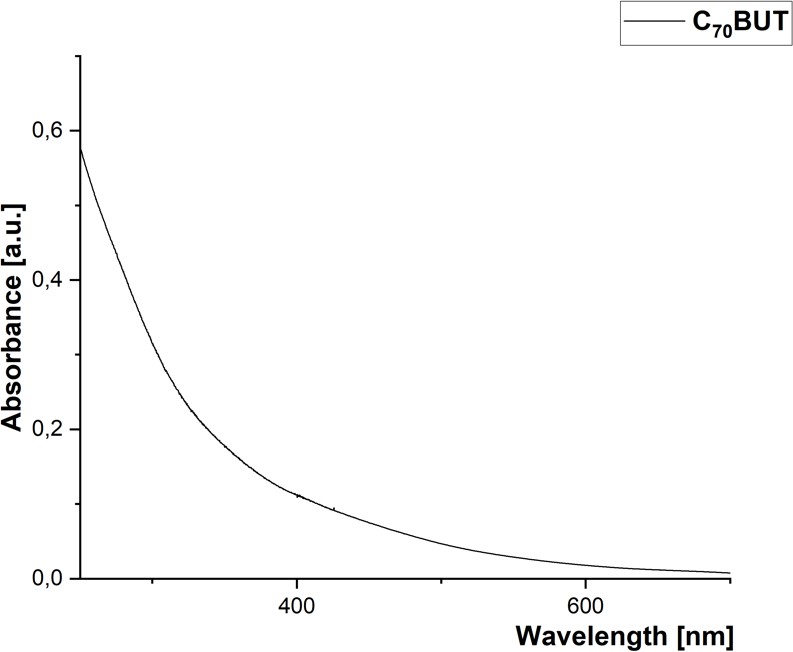


# Figure S9

UV-VIS spectrum of C70BUT.


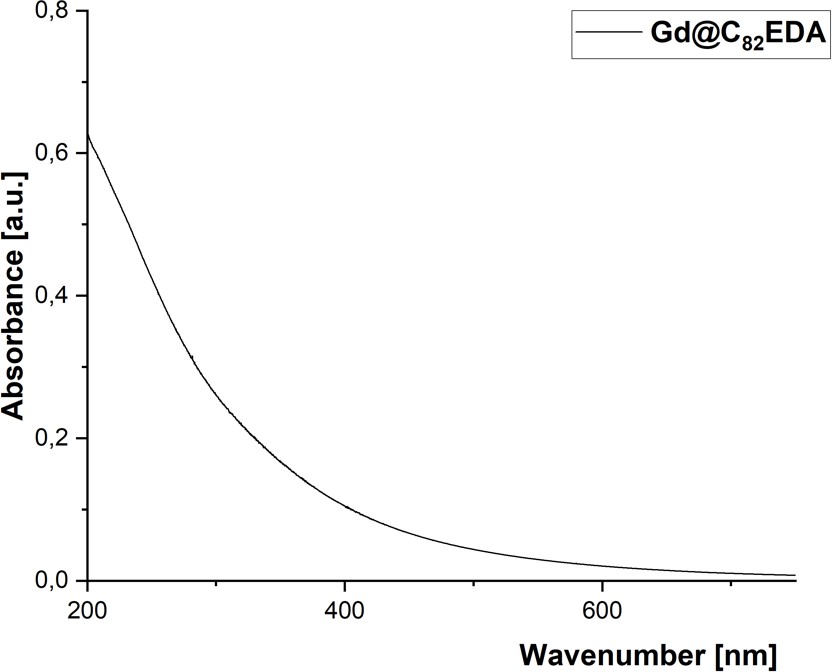


# Figure S10

UV-VIS spectrum of Gd@C82EDA.


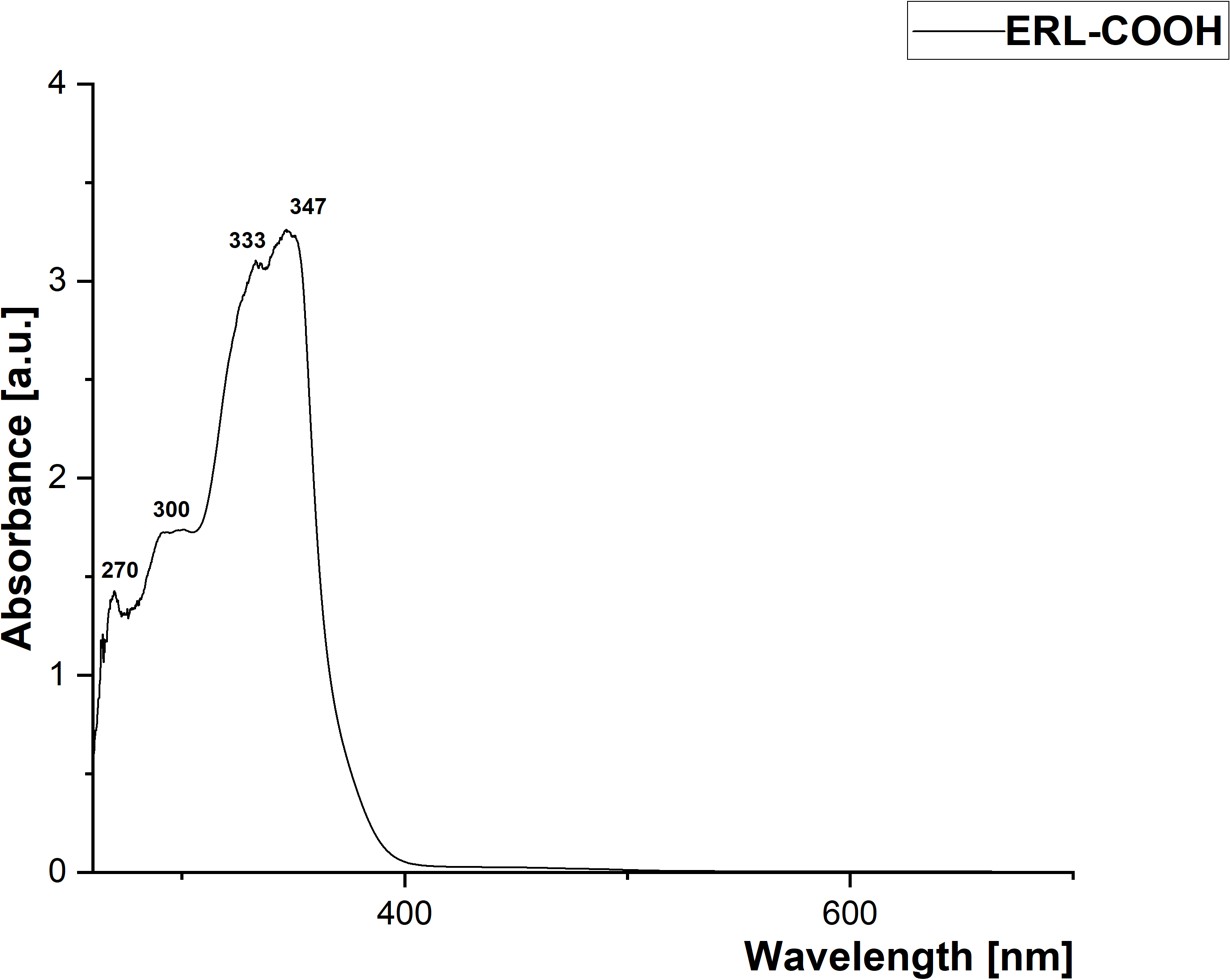


# Figure S11

UV-VIS spectrum of ERL-COOH *[2-(4-(3-((6,7-bis(2-methoxyethoxy)quinazolin-4- yl)amino)phenyl)-1H-1,2,3-triazol-1-yl)acetic acid]*


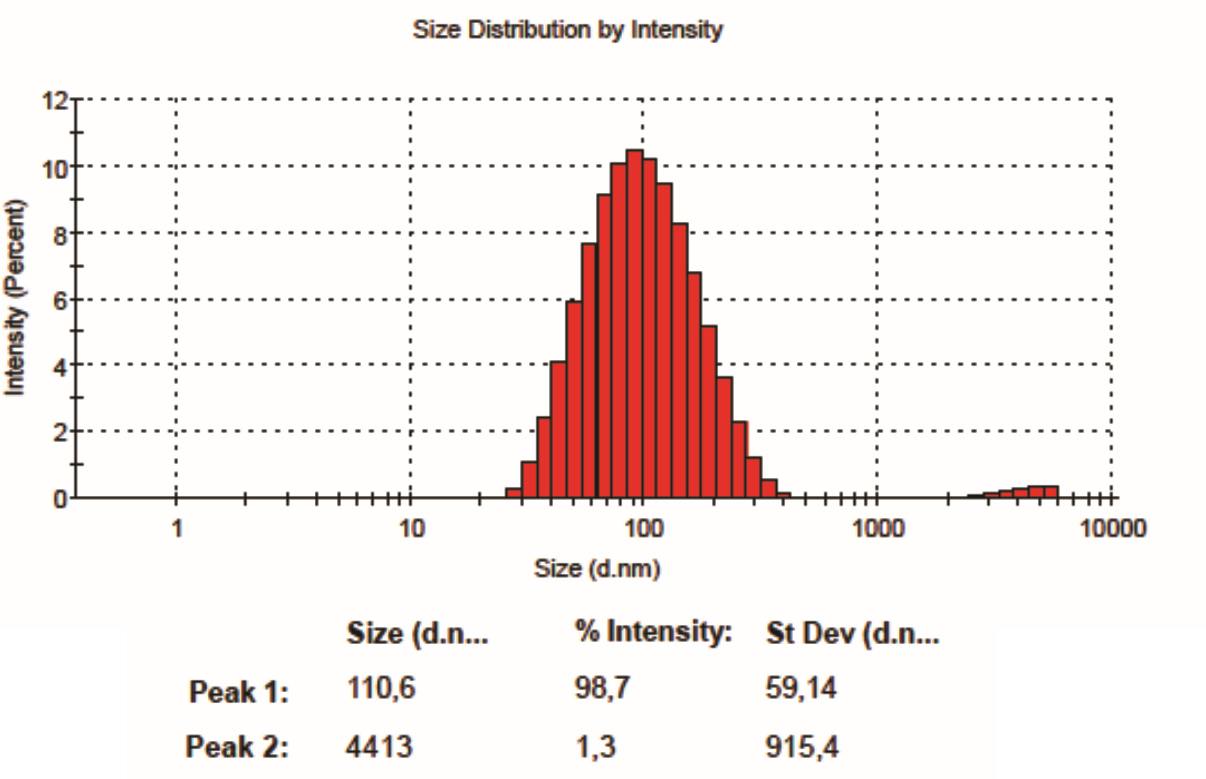


# Figure S12

The size of C60BUT nanoparticles measured using DLS technique (c=0.1 mg/mL).


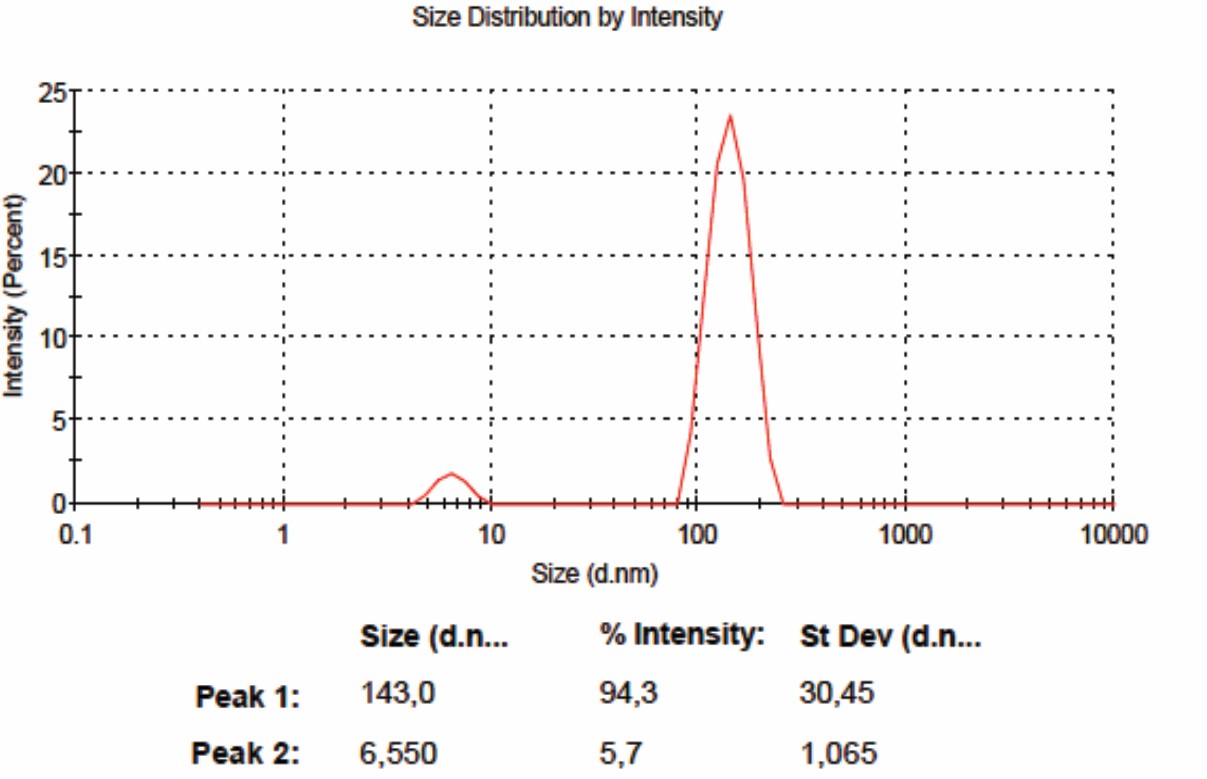


# Figure S13

The size of C70BUT-ERL nanoparticles measured using DLS technique (c=0.1 mg/mL).


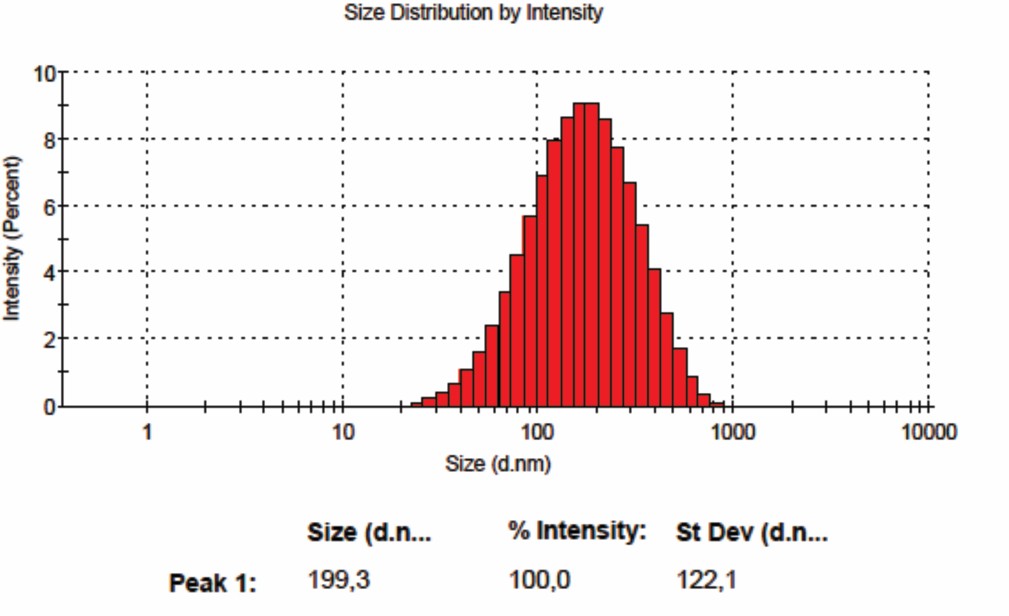


# Figure S13

The size of Gd@C82EDA-ERL nanoparticles measured using DLS technique (c=0.1 mg/mL).


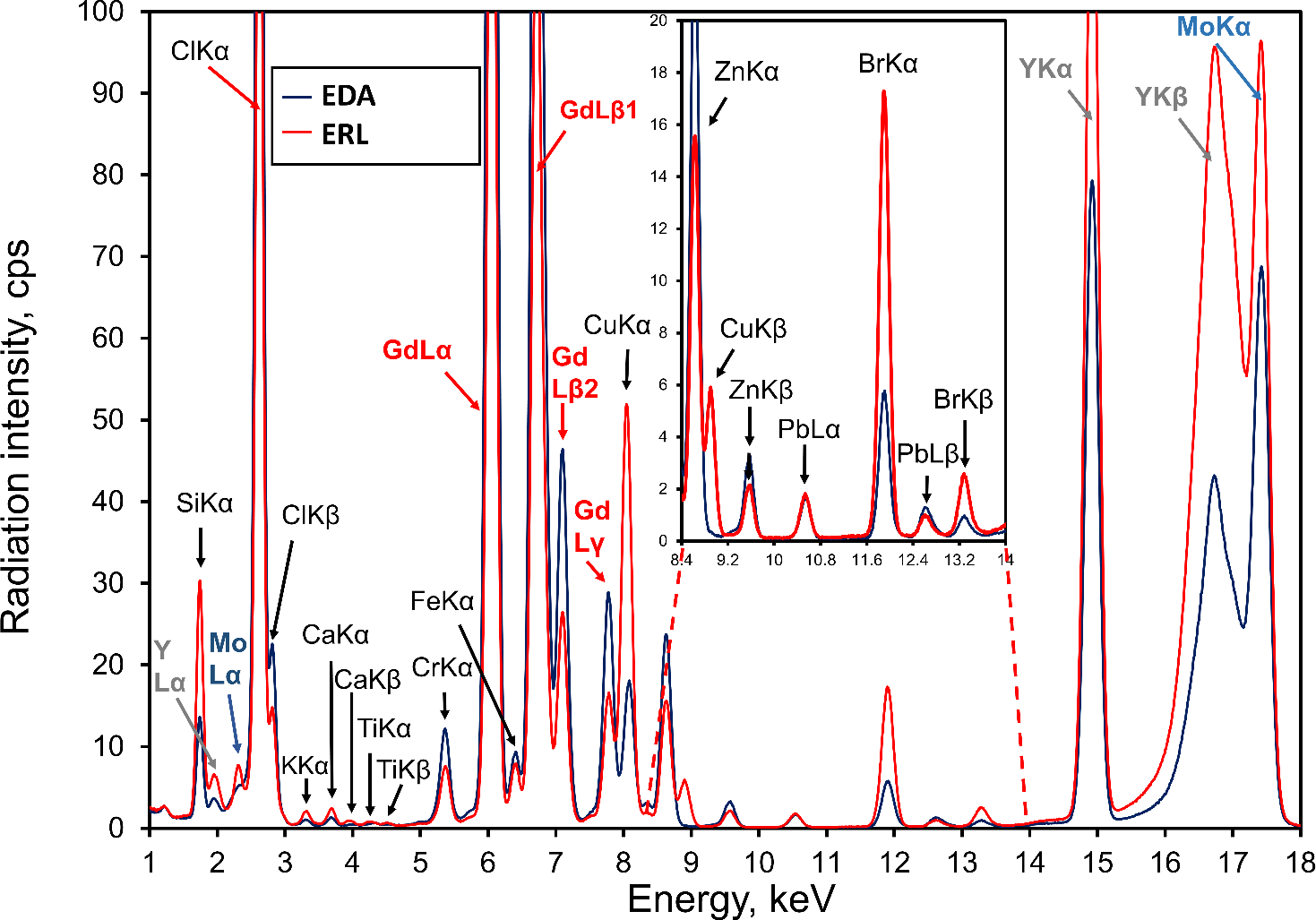


# Figure S14

TXRF spectra of Gd@C82EDA (blue line), and Gd@C82EDA-ERL (red line) suspensions (50 kV, 1000 µA, ambient air atmosphere, 1000 s counting time).

**C60BUT**

| **Element** | **Concentration** |
| --- | --- |
| C | 75.84 |
| N | 6.72 |
| H | 17.44 |

**C70BUT**

| **Element** | **Concentration** |
| --- | --- |
| C | 88.41 |
| N | 7.14 |
| H | 4.45 |

**Gd@C82EDA**

| **Element** | **Concentration** |
| --- | --- |
| C | 62.12 |
| N | 11.41 |
| H | 3.92 |

- C60(NHCH2CH2CH2CH2NH2)x; *N/C* ratio= 28𝑋 = 6.72 ; x= 3

720+48𝑋 75.84

- C70(NHCH2CH2CH2CH2NH2)x; *N/C* ratio = 28𝑋 = 7.14 ; x= 3

840+48𝑋 88.41

- Gd@C82(NHCH2CH2NH2)x; *N/C* ratio = 28𝑋 = 11.41; x= 8

984+24𝑋 62.12

# Table S1

Elemental analysis of synthesized aminofullerenes (*C/N/H*).

|  | **Gd@C82EDA** |  | **Gd@C82EDA ERL** | |
| --- | --- | --- | --- | --- |
| Element | Concentration, µg mL^-1^ | SD | Concentration, µg mL^-1^ | SD,  µg mL^-1^ |
| Chlorine | 242 | 1 | 83.1 | 0.2 |
| Potassium | 0.62 | 0.01 | 0.23 | 0.01 |
| Calcium | 0.38 | 0.01 | 0.361 | 0.004 |
| Titanium | 0.012 | 0.001 | 0.021 | 0.001 |
| Iron | 0.342 | 0.002 | 0.254 | 0.002 |
| Copper | 0.066 | 0.001 | 0.958 | 0.003 |
| Zinc | 0.152 | 0.001 | 0.086 | 0.001 |
| Bromine | 0.010 | 0.001 | 0.075 | 0.001 |
| Lead | 0.029 | 0.001 | 0.009 | 0.001 |
| **Gadolinium** | **13.76** | **0.03** | **9.19** | **0.02** |

# Table S2

The chemical composition of **Gd@C82EDA** and **Gd@C82EDA-ERL** determined with TXRF spectrometry.


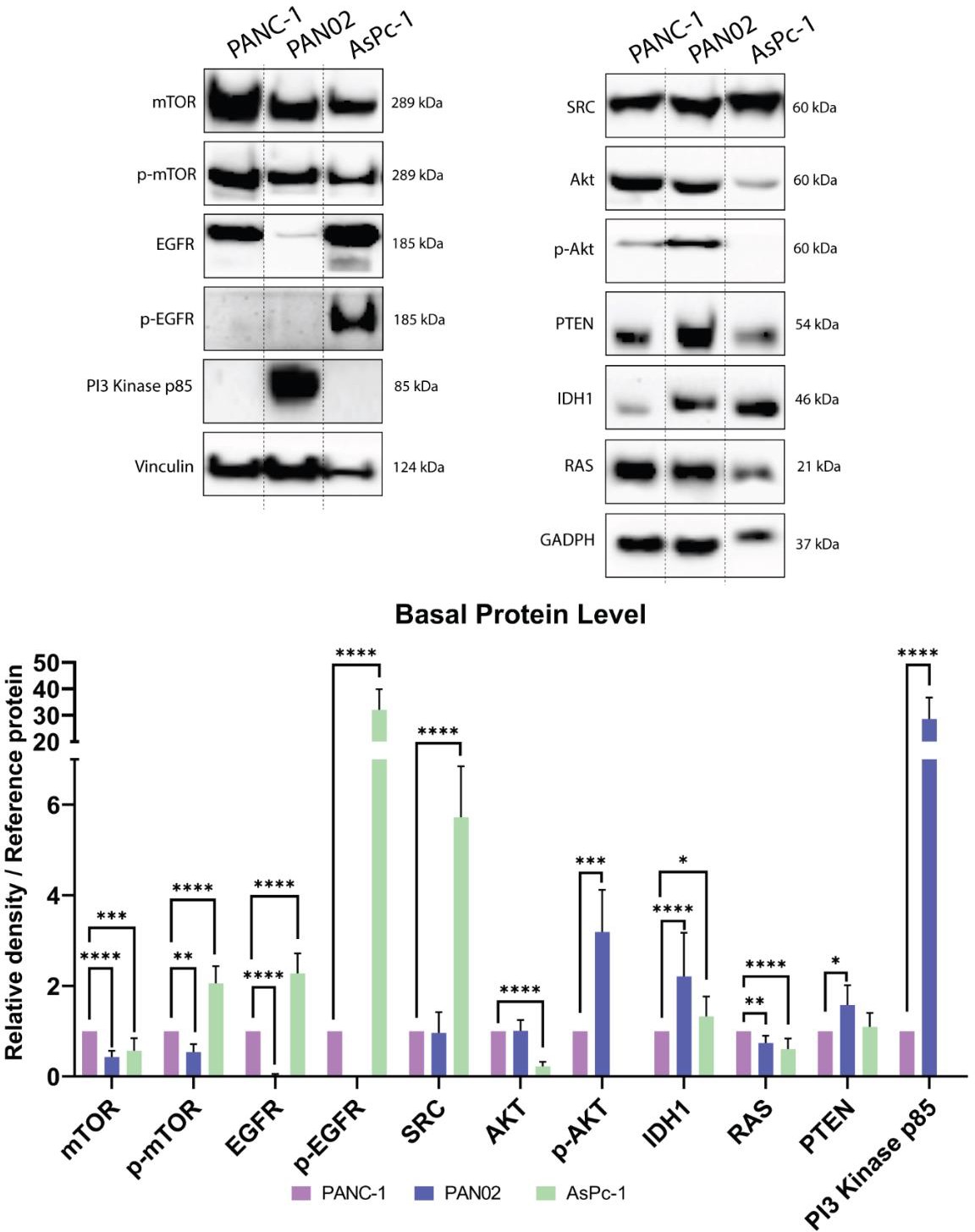


# Figure S15

The landscape of basal protein expression levels associated with EGFR signalling pathway in pancreatic cell lines: PANC-1, PAN02, and AsPC-1. The protein levels are presented in relation to PANC-1 cells. The statistic was done using one-way ANOVA with Šídák's multiple comparison with use of GraphPad 9.0.


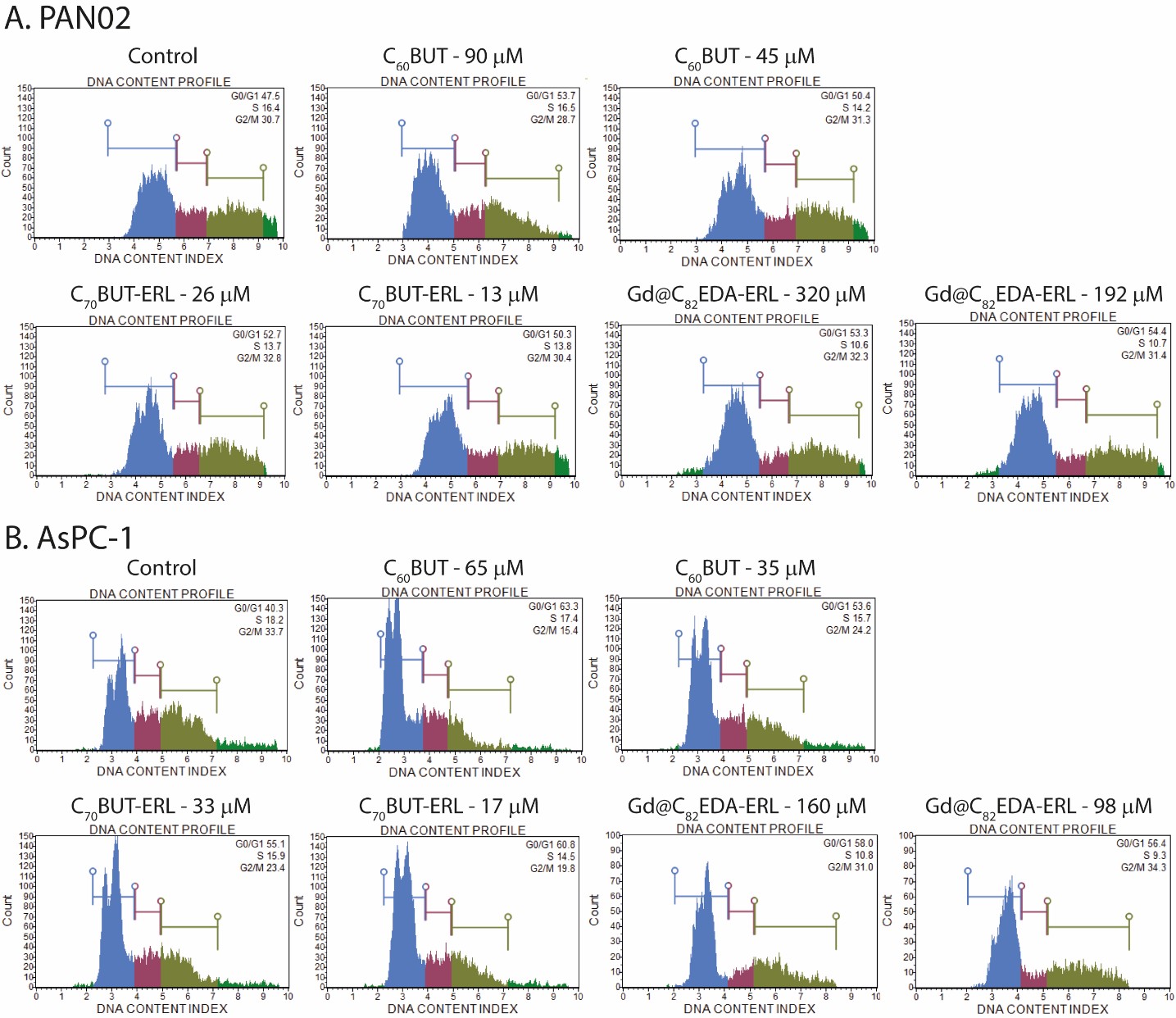


# Figure S16

Representative histograms from cell cycle studies using flow cytometry: (A) PAN02 and (B) AsPC-1 cells.


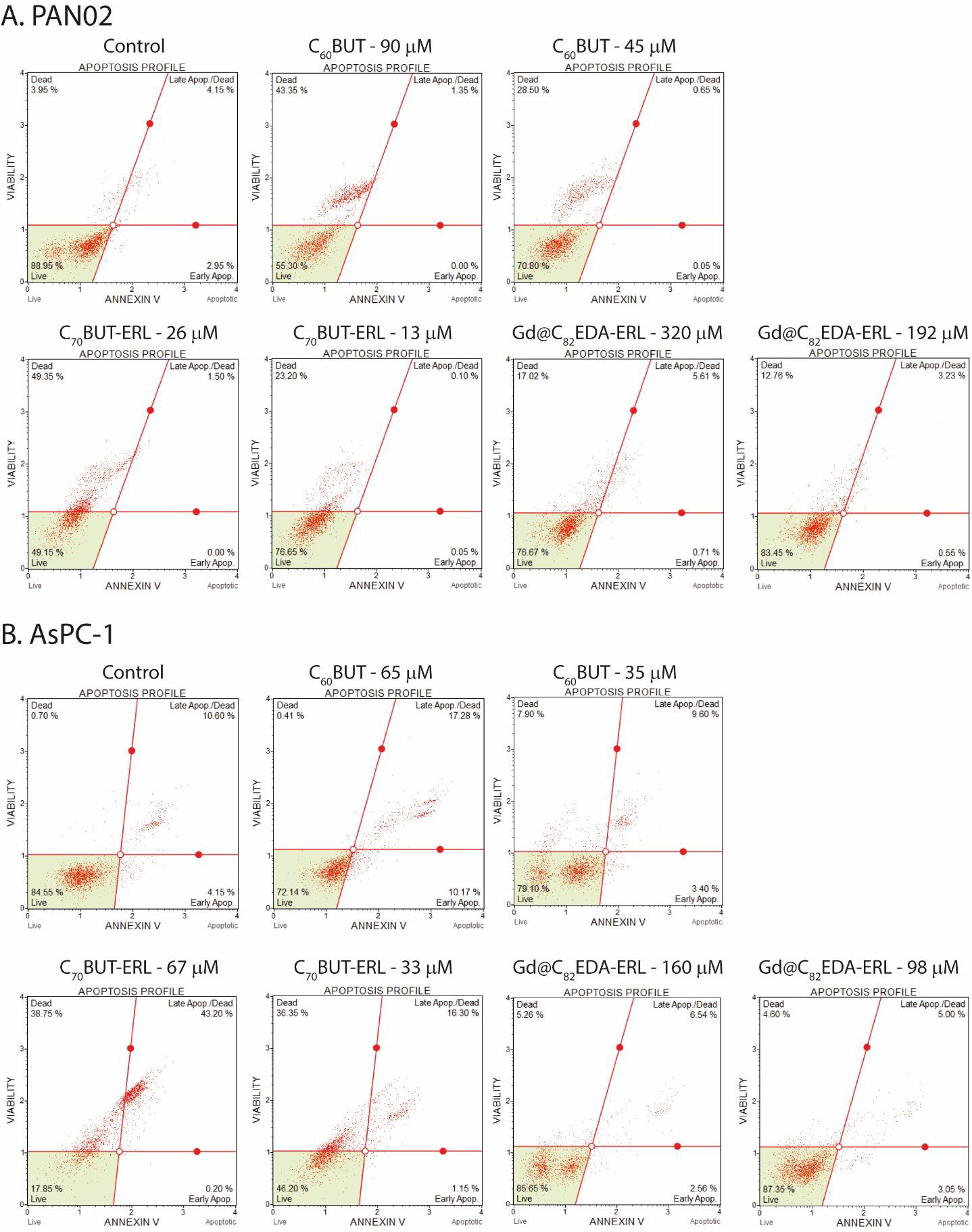


**Figure S17** Representative histograms from apoptosis studies using flow cytometry: (A) PAN02 and (B) AsPC-1 cells.


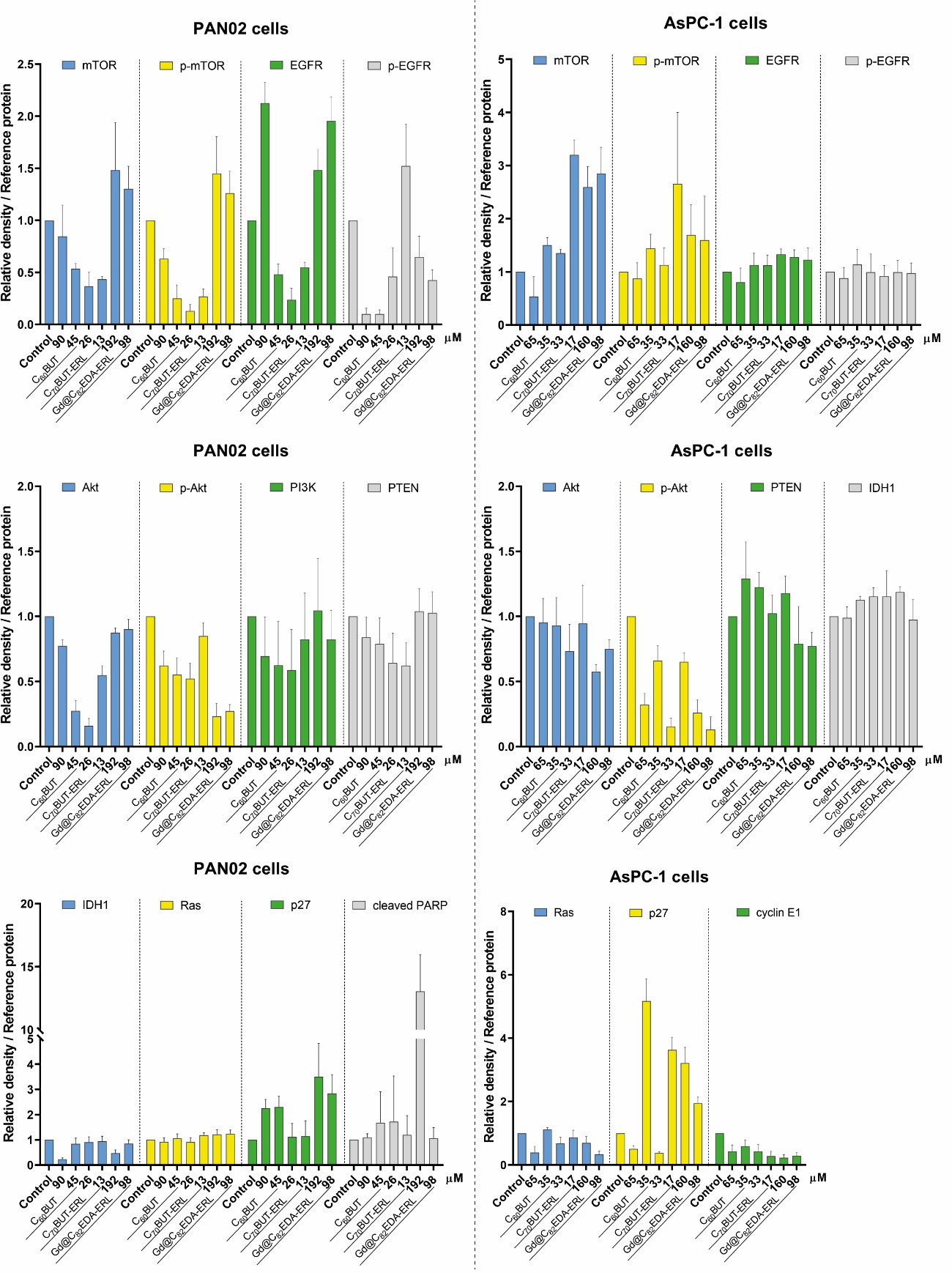


# Figure S18

The densitometric analysis charts of the expression of tested proteins after treatment with nanomaterials. Results were normalized to the reference protein and are from four independent experiments.

# Images of the gels prepared during this study

1. *PAN02 cells (Fig. 7 in main text); exposure: 39.8sec*

We used prestained molecular weight ladder: Novex Sharp Pre-stained Protein Standard (#LC5800, Thermo Scientific).

kDa kDa a


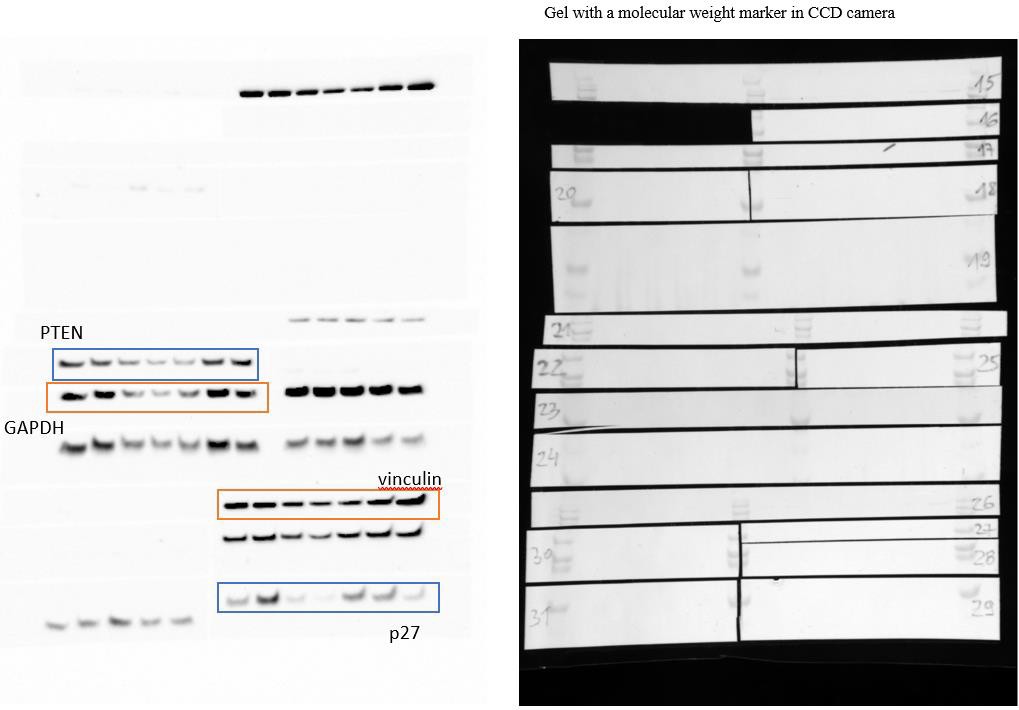


Control, samples →

160 kDa

110 kDa

80 kDa

60 kDa 50kDa 40 kDa

30 kDa

20 kDa

15 kDa

160

110

80 kD

60 kD

50kD

40

🡨 Samples, Control

30

a

a kDa

kDa

a a a a


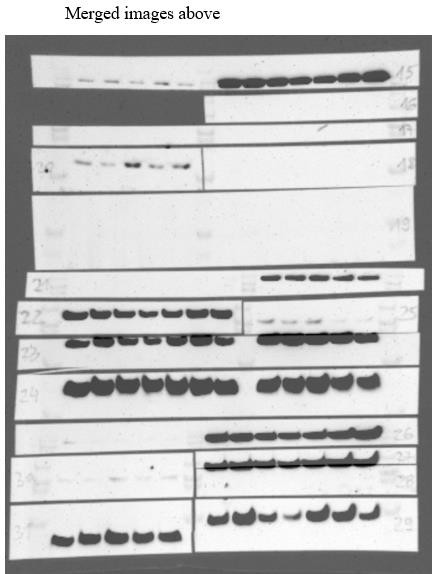


160 kDa

110 kDa

80 kDa

60 kDa 50kDa 40 kDa

30 kDa

20 kDa

15 kDa

160 kD

110 kD

80 kD

60 kD

50kDa 40 kD

30

a

kDa

1. *PAN02 cells (Fig. 7 in main text); exposure 39.8sec*

We used prestained molecular weight ladder: Novex Sharp Pre-stained Protein Standard (#LC5800).


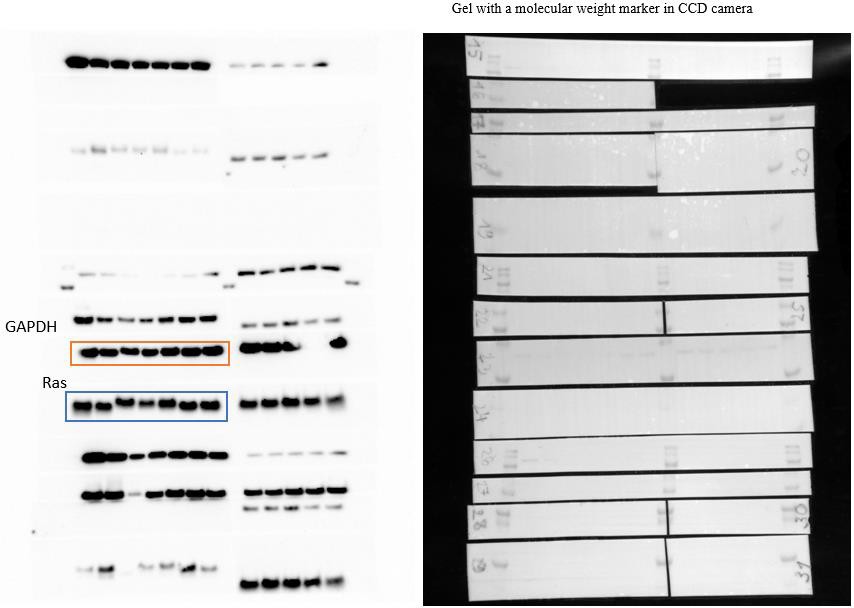


260 kDa

160 kDa

110 kDa

80 kDa

60 kDa

Control, samples →

50kDa

40 kDa

30 kDa

20 kDa

15 kDa


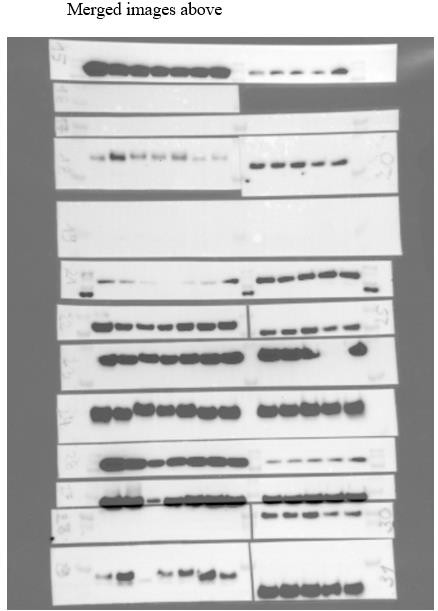


260 kDa

160 kDa

110 kDa

80 kDa

60 kDa

50kDa 40 kDa

30 kDa

20 kDa

15 kDa

1. *PAN02 cells (Fig. 7 in main text); exposure 10.0sec + 39.8sec + 248.0 sec*

We used prestained molecular weight ladder: Spectra Multicolor High Range Protein Ladder (#26625, Thermo Scientific) and Novex Sharp Pre-stained Protein Standard (#LC5800).


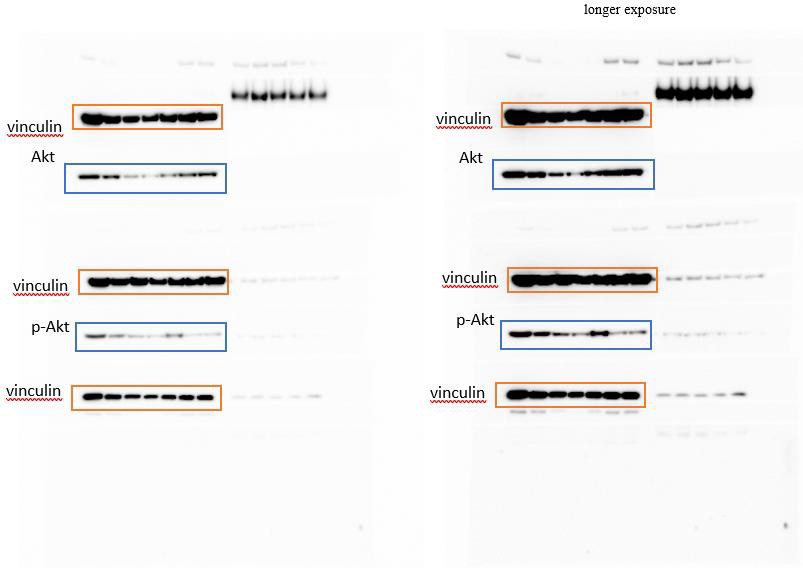


Control, samples 🡺


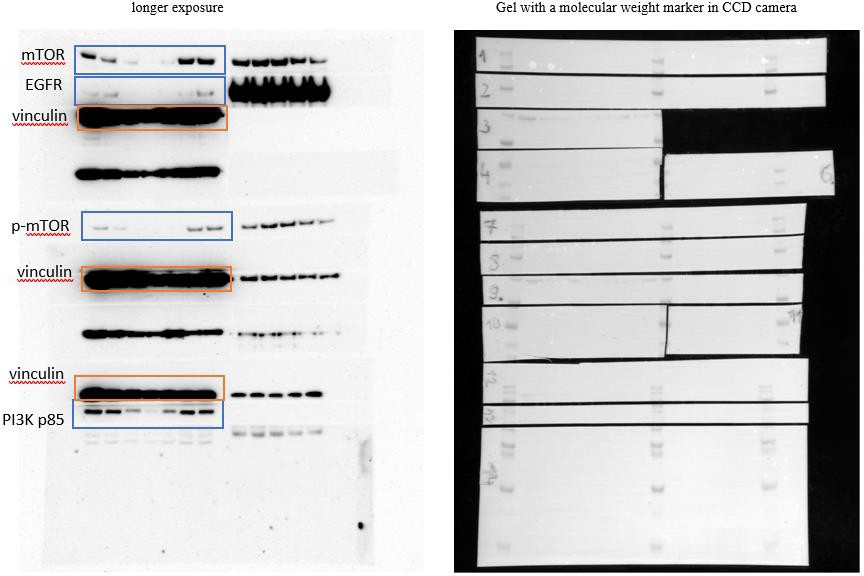


Control, samples 🡺

**300 kDa**

**250 kDa**

**180 kDa**

**130 kDa**

**100 kDa**

**70 kDa**

**50 kDa**

**40 kDa**

**300 kDa**

**250 kDa**

**180 kDa**

**130 kDa**

**100 kDa**

**70 kDa**

**50 kDa**

260 kDa

160 kDa

110 kDa

80 kDa

60 kDa 50kDa 40 kDa

30 kDa

20 kDa

15 kDa


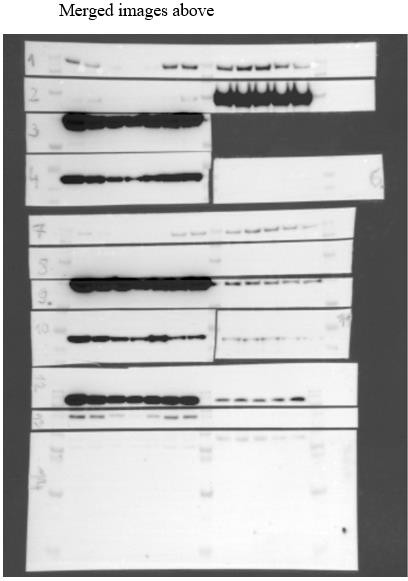


**300 kDa**

**250 kDa**

**180 kDa**

**130 kDa**

**100 kDa**

**70 kDa**

**50 kDa**

**40 kDa**

**300 kDa**

**250 kDa**

**180 kDa**

**130 kDa**

**100 kDa**

**70 kDa**

**50 kDa**

260 kDa

160 kDa

110 kDa

80 kDa

60 kDa

50kDa 40 kDa

30 kDa

20 kDa

15 kDa

1. *PAN02 cells (Fig. 7 in main text) – exposure 813.2sec*

We used prestained molecular weight ladder: Spectra Multicolor High Range Protein Ladder (#26625).

**250 kDa**


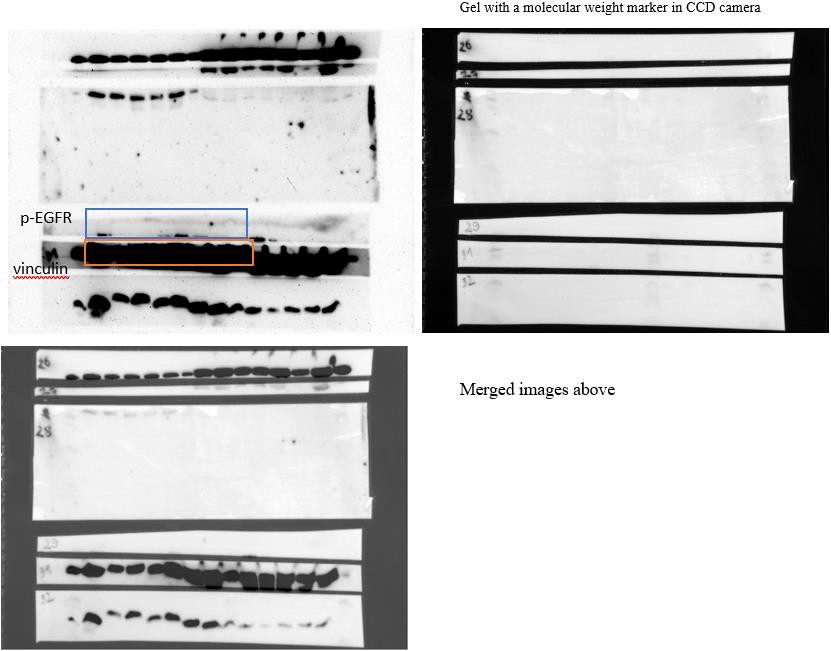


Control, samples 🡺

**250 kDa**

**130 kDa**

**100 kDa**

**70 kDa**

**50 kDa**

**40 kDa**

**130 kDa**

**100 kDa**

**70 kDa**

**50 kDa**

**40 kDa**

1. *PAN02 cells (Fig. 7 in main text) – exposure 724.0sec*

We used prestained molecular weight ladder: Novex Sharp Pre-stained Protein Standard (#LC5800).


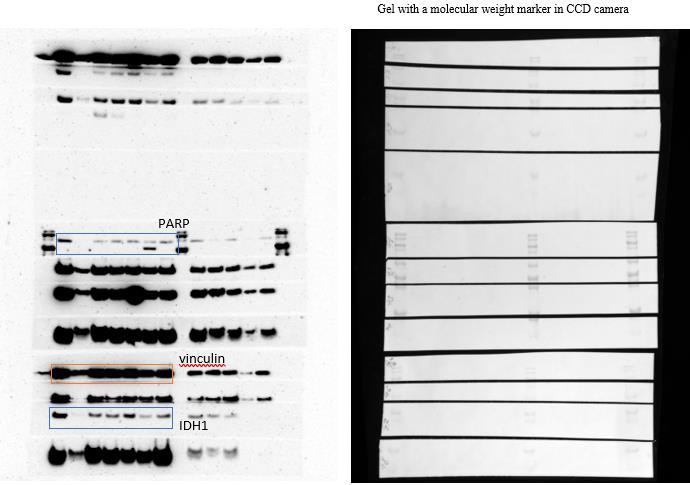


Control, samples 🡺

260 kDa

160 kDa

110 kDa

80 kDa

60 kDa

50kDa 40 kDa

30 kDa

20 kDa


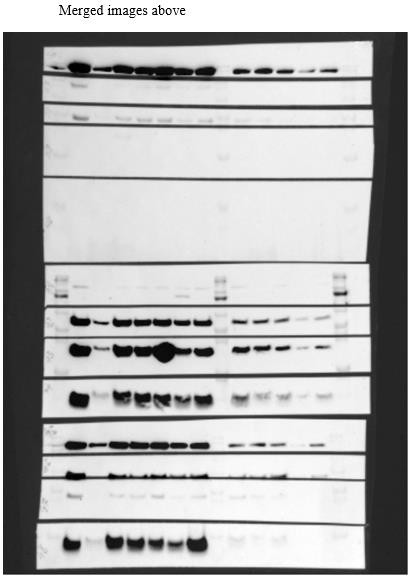


260 kDa

160 kDa

110 kDa

80 kDa

60 kDa

50kDa 40 kDa

30 kDa

20 kDa

1. *AsPC-1 cells (Fig. 7 in main text) – exposure 337.2sec + 813.2sec*

We used prestained molecular weight ladder: Spectra Multicolor High Range Protein Ladder (#26625).


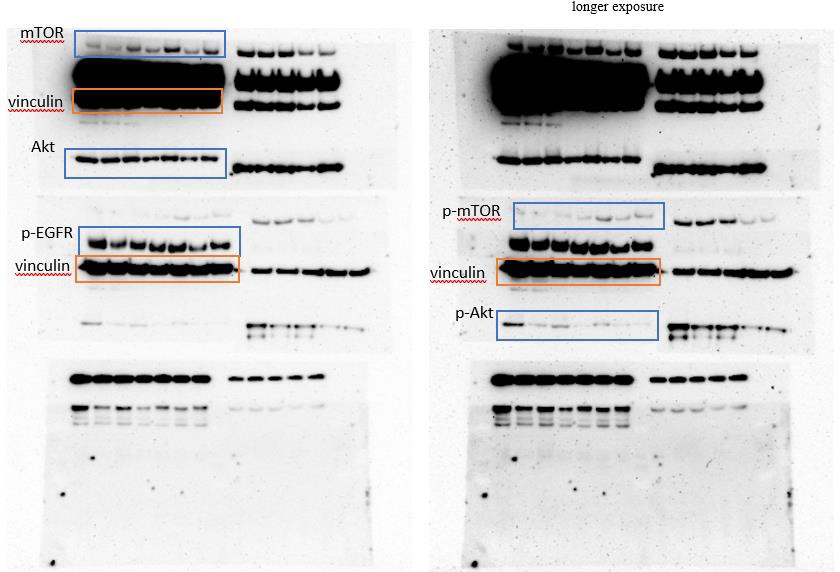


Control, samples 🡺

Control, samples 🡺


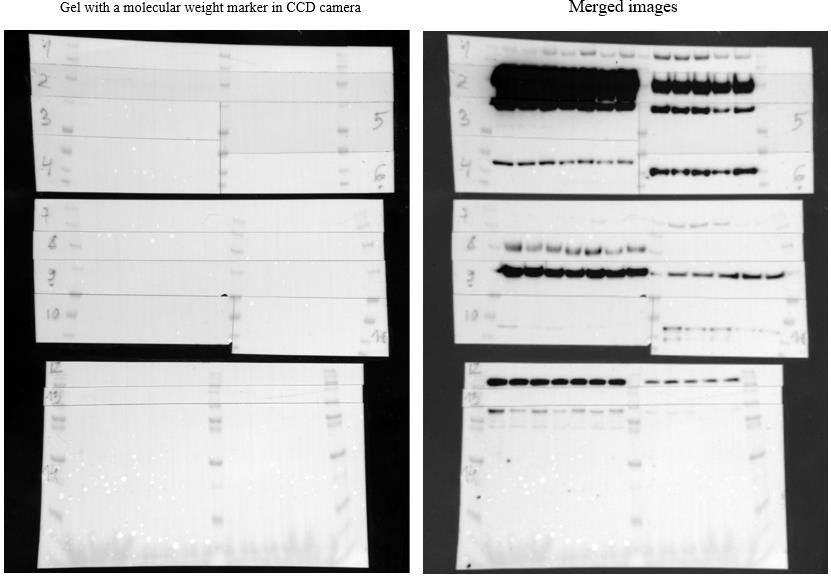


**300 kDa**

**250 kDa**

**180 kDa**

**130 kDa**

**100 kDa**

**70 kDa**

**50 kDa**

**40 kDa**

**300 kDa**

**250 kDa**

**180 kDa**

**130 kDa**

**100 kDa**

**70 kDa**

**50 kDa**

**40 kDa**

1. *AsPC-1 cells (Fig. 7 in main text) – exposure 456.2sec*

We used prestained molecular weight ladder: Novex Sharp Pre-stained Protein Standard (#LC5800).

110 kDa


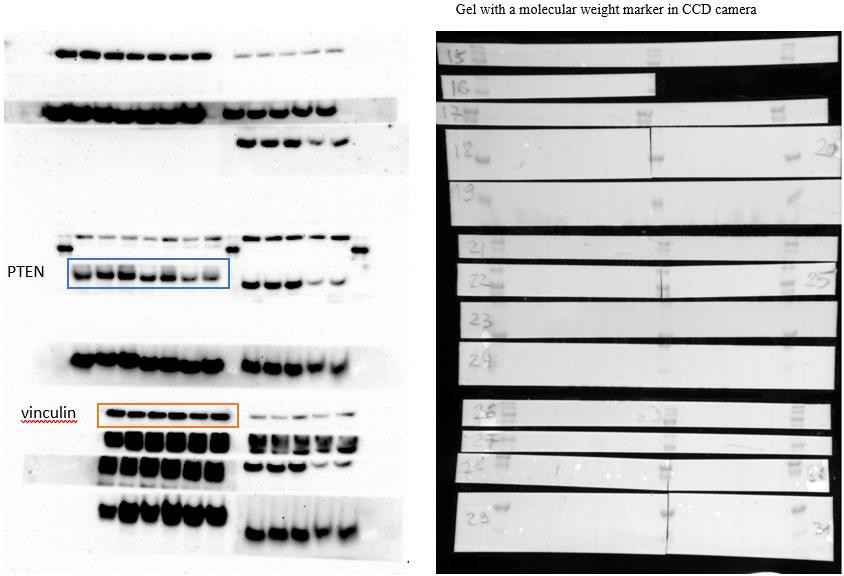


Control, samples 🡺

80 kDa

60 kDa

50kDa 40 kDa

30 kDa

20 kDa

160 kDa

110 kDa

80 kDa

60 kDa

50kDa 40 kDa

30 kDa

20 kDa

110 kDa


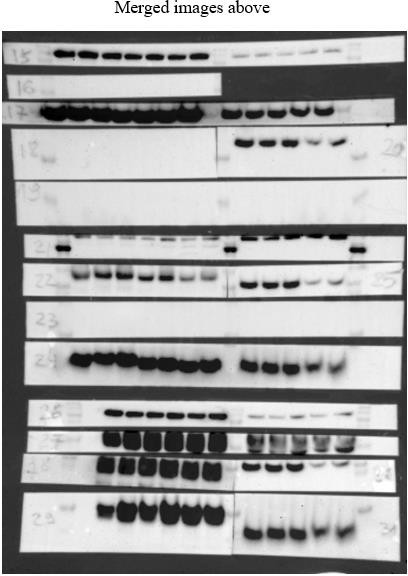


80 kDa

60 kDa

50kDa 40 kDa

30 kDa

20 kDa

160 kDa

110 kDa

80 kDa

60 kDa

50kDa 40 kDa

30 kDa

20 kDa

1. *AsPC-1 cells (Fig. 7 in main text) - 99.2sec*

We used prestained molecular weight ladder: Spectra Multicolor High Range Protein Ladder (#26625).

**a a**


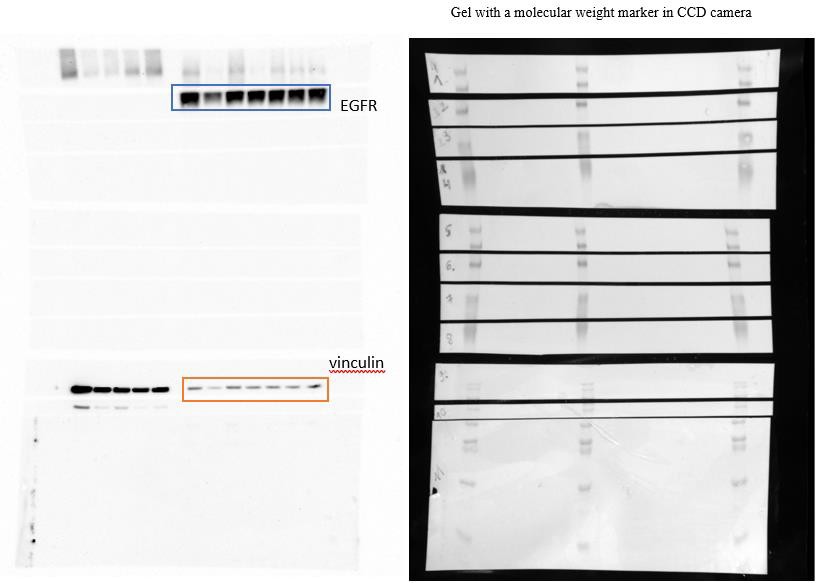


Control, samples 🡺

**300 kD**

**250 kD**

**180 kD**

**130 kD**

**100 kD**

**70 kDa**

260 kDa

160 kDa

110 kDa

80 kDa

60 kDa 50kDa 40 kDa

30 kDa

20 kDa

**a**

**a a**

**a**


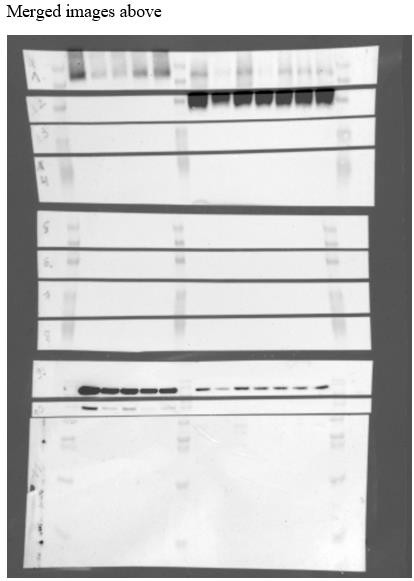


**300 kD**

**250 kDa**

**180 kDa**

**130 kDa**

**100 kDa**

**70 kDa**

260 kDa

160 kDa

110 kDa

80 kDa

60 kDa 50kDa 40 kDa

30 kDa

20 kDa

1. *AsPC-1 cells (Fig. 7 in main text) – exposure 29.0sec + 337.2sec + 1051.2sec*

We used prestained molecular weight ladder: Novex Sharp Pre-stained Protein Standard (#LC5800).


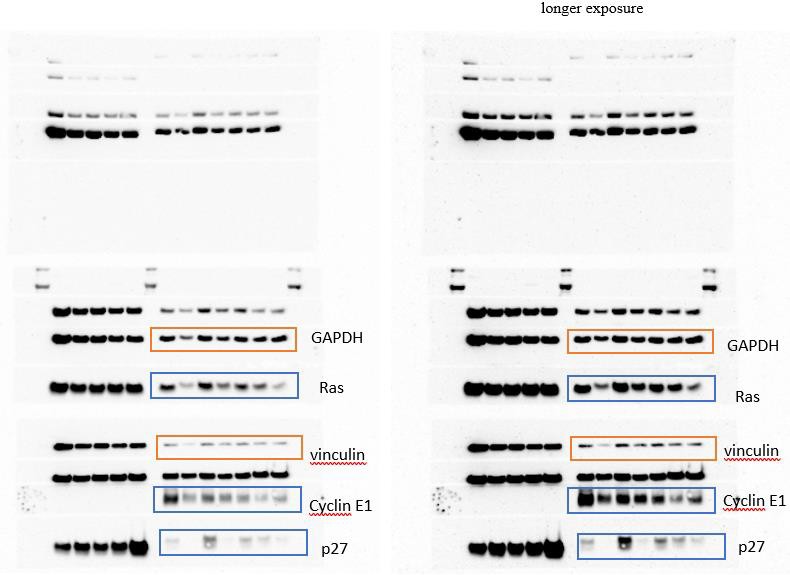


Control, samples 🡺


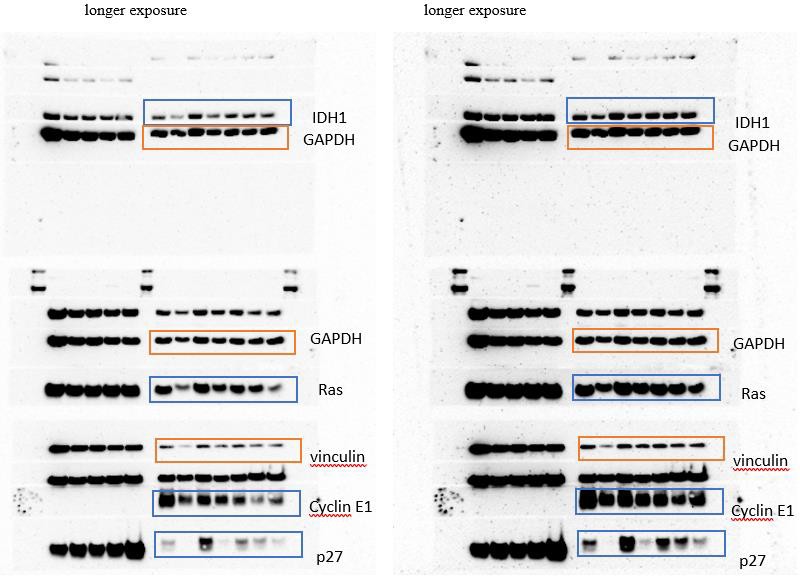


Control, samples 🡺

Control, samples 🡺


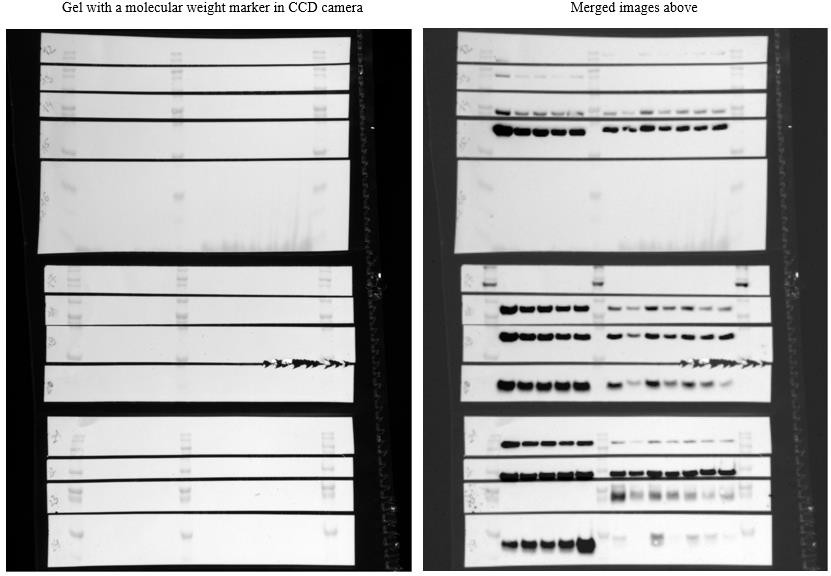


260 kDa

160 kDa

110 kDa

80 kDa

60 kDa

50kDa 40 kDa

30 kDa

20 kDa

160 kDa

110 kDa

80 kDa

60 kDa

50kDa 40 kDa

30 kDa

20 kDa

260 kDa

160 kDa

110 kDa

80 kDa

60 kDa

50kDa

40 kDa

30 kDa

1. *Basal expression (Fig. S15) – exposure 30.0 sec*

We used prestained molecular weight ladder: Spectra Multicolor High Range Protein Ladder (#26625).


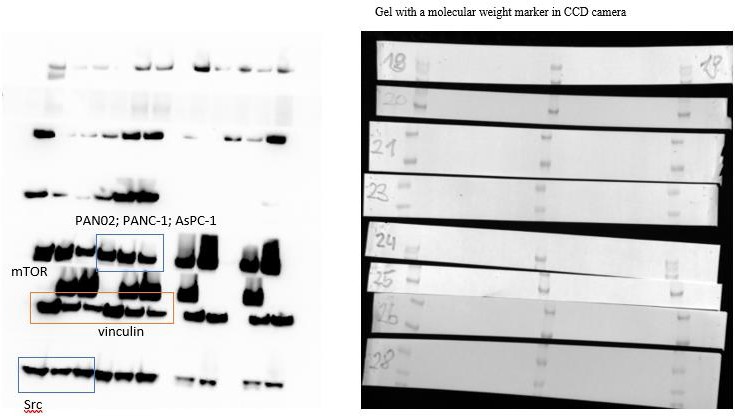


**300 kDa**

**250 kDa**

**180 kDa**

**130 kDa**

**100 kDa**

**70 kDa**

**50 kDa**

**a**


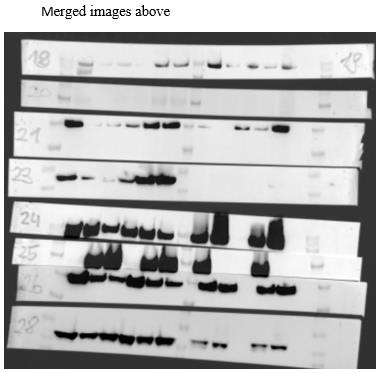


**300 kDa**

**250 kDa**

**180 kDa**

**130 kDa**

**100 kDa**

**70 kD**

**50 kD**

**a**

1. *Basal expression (Fig. S15) – exposure 30.0 sec + 120.0 sec*

We used prestained molecular weight ladder: Novex Sharp Pre-stained Protein Standard (#LC5800).


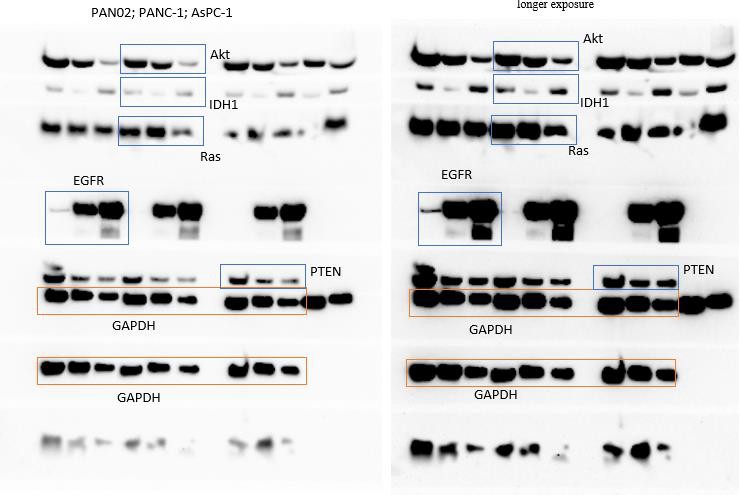


**80 kDa**


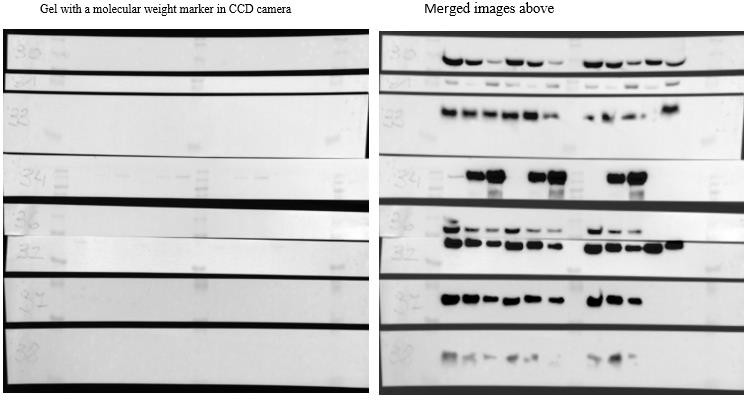


**80 kDa**

**60 kDa**

**50 kDa**

**40 kDa**

**20 kDa**

**15 kDa**

**260 kDa**

**160 kDa**

**110 kDa**

**60 kDa**

**50 kDa**

**30 kDa**

**40 kDa**

**30 kDa**

**15 kDa**

**60 kDa**

**50 kDa**

**40 kDa**

**20 kDa**

**15 kDa**

**260 kDa**

**160 kDa**

**110 kDa**

**60 kDa**

**50 kDa**

**30 kDa**

**40 kDa**

**30 kDa**

**15 kDa**

1. *Basal expression (Fig. S15) - exposure 30.0 sec + 660.0 sec*

We used prestained molecular weight ladder: Spectra Multicolor High Range Protein Ladder (#26625).


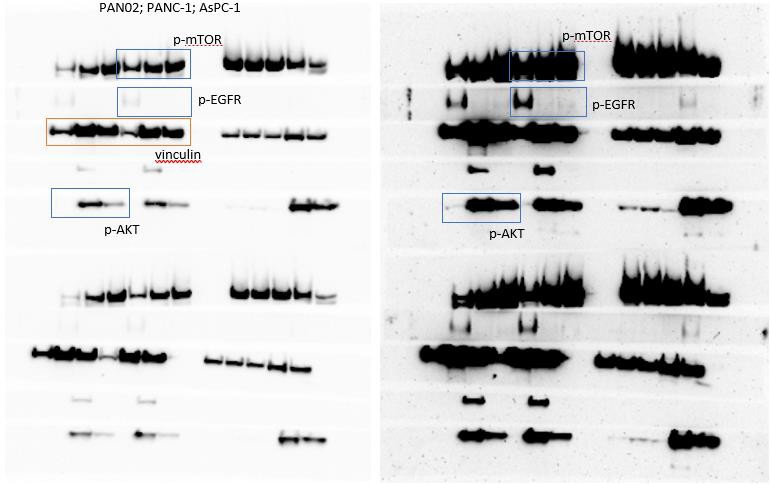


Longer exposure


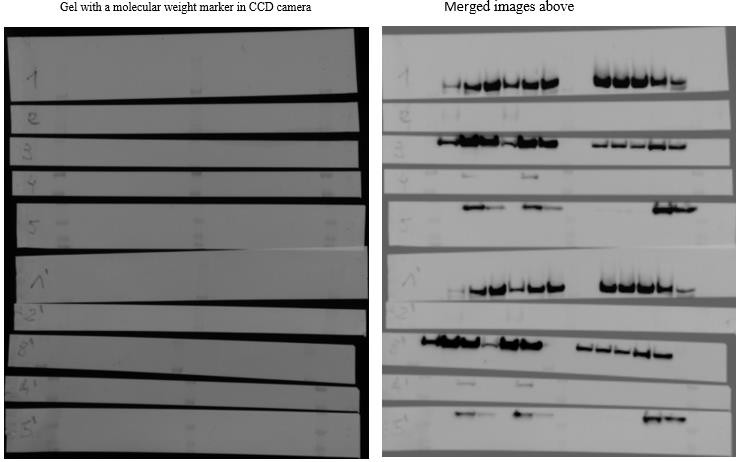


**300 kDa**

**250 kDa**

**180 kDa**

**130 kDa**

**100 kDa**

**70 kDa**

**50 kDa**

**40 kDa**

1. *Basal expression (Fig. S15) - exposure 120.0 sec*

We used prestained molecular weight ladder: Novex Sharp Pre-stained Protein Standard (#LC5800).


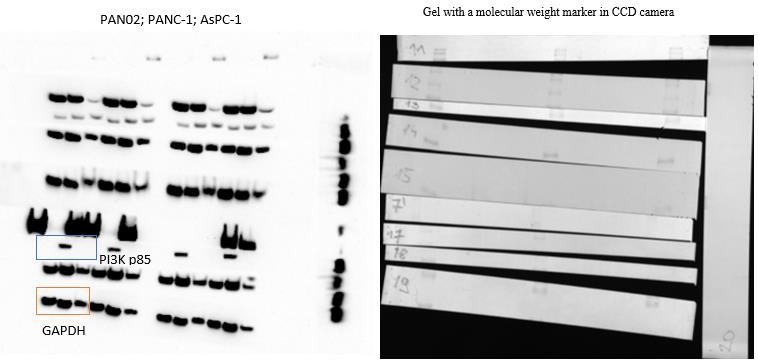


**85 kDa**

**60 kDa**

**50 kDa**

**40 kDa**

**30 kDa**


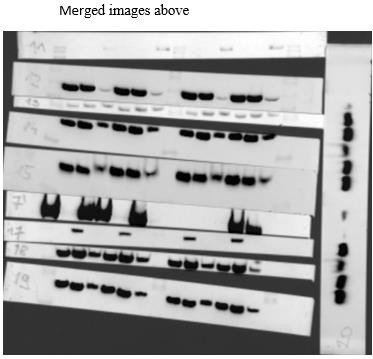


**85 kDa**

**60 kDa**

**50 kDa**

**40 kDa**

**30 kDa**

# Figure S19

The uncropped and unmodified blots. Proteins relevant to this study are marked in boxes along with reference proteins.

# REFERENCES

1. J. Dyke, A. Groves, A. Morris, J. Ogden, A. Dias, A. Oliveira, M. Costa, M. Barros, M. Cabral and A. Moutinho, *Journal of the American Chemical Society*, 1997, **119**, 6883-6887.
