# Supplementary material for: Aminofullerenes as targeted inhibitors of EGFR: from pancreatic cancer inhibitors to Drosophila m. Toxicology
Source: Nanomedicine (Lond). 2025 Feb 7;20(6):585–601. doi: 10.1080/17435889.2025.2461985 (PMC11881853; doi:10.1080/17435889.2025.2461985)
Supplement: Supplemental Material [file INNM_A_2461985_SM9658.pdf]

Aminofullerenes targeting EGFR: From pancreatic cancer inhibition to toxicology in Drosophila melanogaster

1. **Chemistry**

- *Spectral data of 2-azidoacetic acid*
- *NMR spectrum of* ERL-COOH *(^{1}H- and ^{13}C-NMR)*
- *HRMS spectrum of ERL-COOH*
- *FT-IR spectra of synthesized aminofullerenes and ERL-COOH*
- *UV-VIS spectra of synthesized aminofullerenes and ERL-COOH*
- *DLS measurements of synthesized aminofullerenes*
- *TXRF spectrum of gadofullerene Gd@C_{82}EDA and Gd@C_{82}EDA-ERL*
- *Elemental analysis of synthesized aminofullerenes*
- *The chemical composition of Gd@C_{82}EDA and Gd@C_{82}EDA-ERL determined with TXRF spectrometry.*

2. Biology

- *Basal protein level in tested pancreatic cells*
- *Histograms from cell cycle studies*
- *Histograms from apoptosis studies*
- *Densitometric analysis of studied proteins after treatment with nanomaterials*
- *Uncropped and unmodified blots*

- *Spectral data of 2-azidoacetic acid*

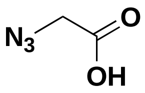

The compound was synthesized using modified procedure of Dyke[1].

^{1}H-NMR (*500 MHz, CDCl$_3$, ppm*): 8.39 (s, 1H, -COOH), 3.97 (s, 2H, CH_2).

^{13}C-NMR (*126 MHz, CDCl$_3$, ppm*): 173.39 (C=O), 50.04 (CH_2).

- ***Spectral data of ERL-COOH (2-(4-(3-((6,7-bis(2-methoxyethoxy)quinazolin-4-yl)amino)phenyl)-1*H*-1,2,3-triazol-1-yl)acetic acid)***

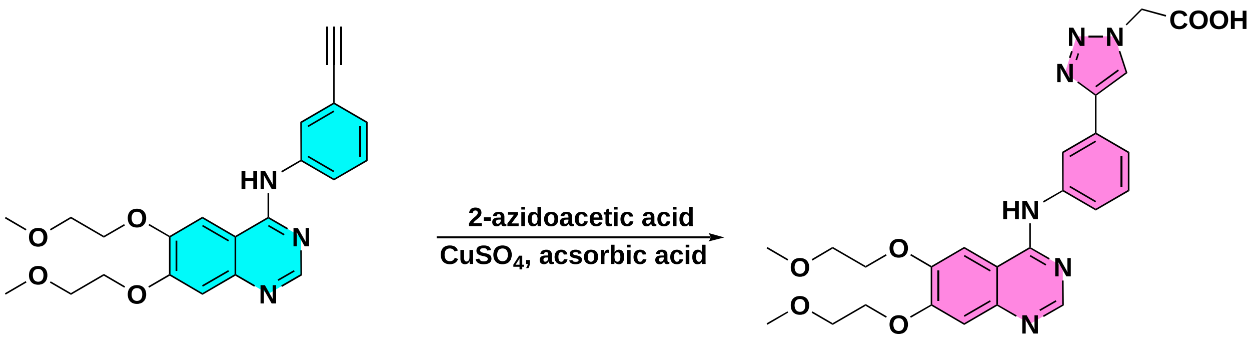

ERLOTINIB (ERL)

(ERL-COOH)

^{1}H-NMR (*500 MHz, CDCl$_3$, ppm*): *δ 9.91 (s, 1H, N**H**), 8.47 (s, 1H, aryl **H**), 8.41 (s, 1H, aryl **H**), 8.35 (t, J = 2.0 Hz, 1H, aryl **H**), 8.12 (s, 1H, aryl **H**), 7.95 (ddd, J = 8.0, 2.2, 1.0 Hz, 1H, aryl **H**), 7.54 (dt, J = 7.6, 1.4 Hz, 1H, aryl **H**), 7.41 (t, J = 7.9 Hz, 1H, aryl **H**), 7.21 (s, 1H, aryl **H**), 4.86 (s, 2H, -C**H**$_2$), 4.38 – 4.33 (m, 2H, -C**H**$_2$), 4.31 – 4.25 (m, 2H, -C**H**$_2$), 3.81 – 3.71 (m, 4H, -C**H**$_2$), 3.36 (d, J = 5.5 Hz, 6H, -C**H**$_3$).*

^{13}C-NMR (*126 MHz, CDCl$_3$, ppm*): *δ 169.54 (COOH), 156.94 (aryl **C**), 153.94 (aryl **C**), 153.37 (aryl **C**), 148.46 (aryl **C**), 147.37 (aryl **C**), 146.27 (triazole ring **C**), 140.65 (aryl **C**), 131.85 (triazole ring **C**), 129.26 (aryl **C**), 123.02 (aryl **C**), 121.89 (aryl **C**), 120.44 (aryl **C**), 119.20 (aryl **C**), 109.60 (aryl **C**), 108.56 (aryl **C**), 104.14 (aryl **C**), 70.58 (d, J = 12.8 Hz, **C**H$_2$), 68.95 (**C**H$_2$), 68.46 (**C**H$_2$), 58.82 (**C**H$_3$), 49.02 (**C**H$_2$).*

Melting point: 217 °C

HR-ESI MS (negative polarisation): *Chemical Formula [M-H]$^-$: $C_{24}H_{25}N_6O_6$; Exact Mass (calculated: 493.1841 Da); measured: 493.1829 Da.*

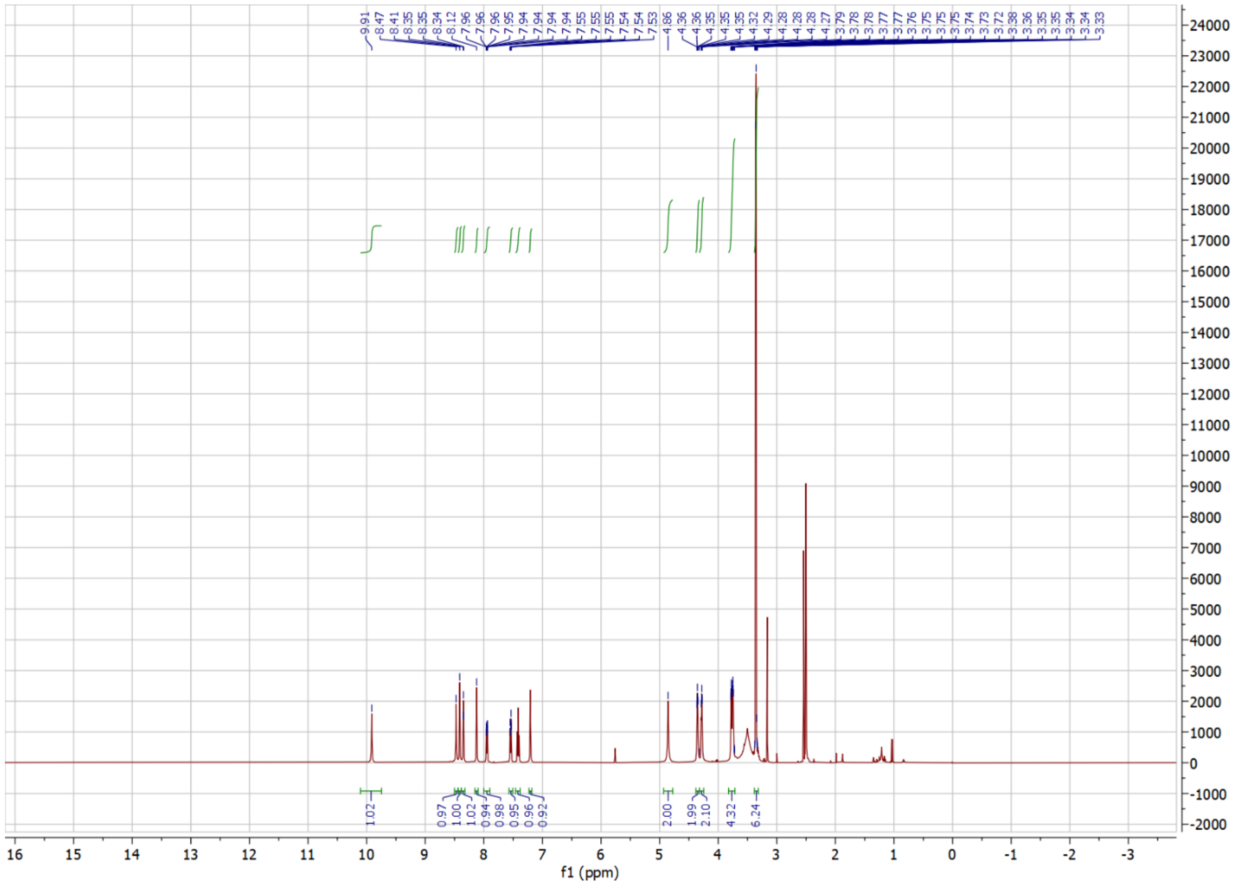

Figure S1

^{1}H-NMR of ERL-COOH.

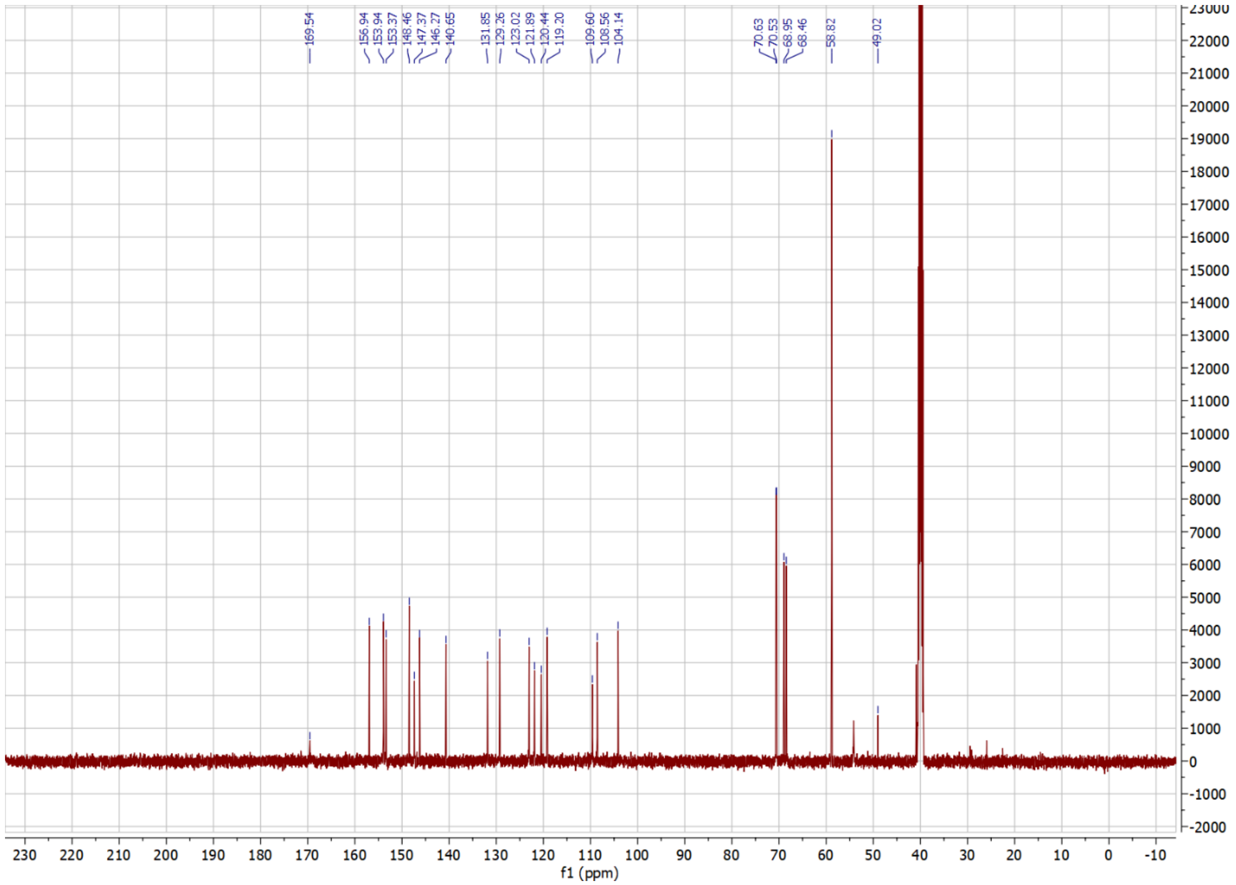

Figure S2

^{13}C-NMR of ERL-COOH

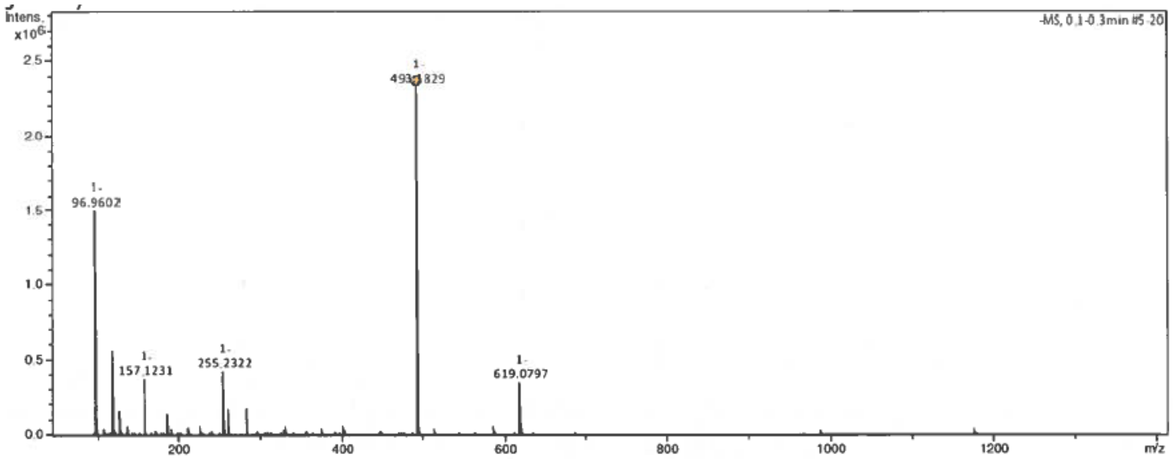

Meas. m/z	#	Ion Formula	m/z	err [ppm]	mSigma	# Sigma	Score	rdb	e⁻ Conf	N-Rule
493.1829	1	**C24H25N6O6**	493.1841	**2.4**	45.3	1	100.00	15.5	even	ok

Figure S3

High resolution ESI spectrum of compound ERL-COOH

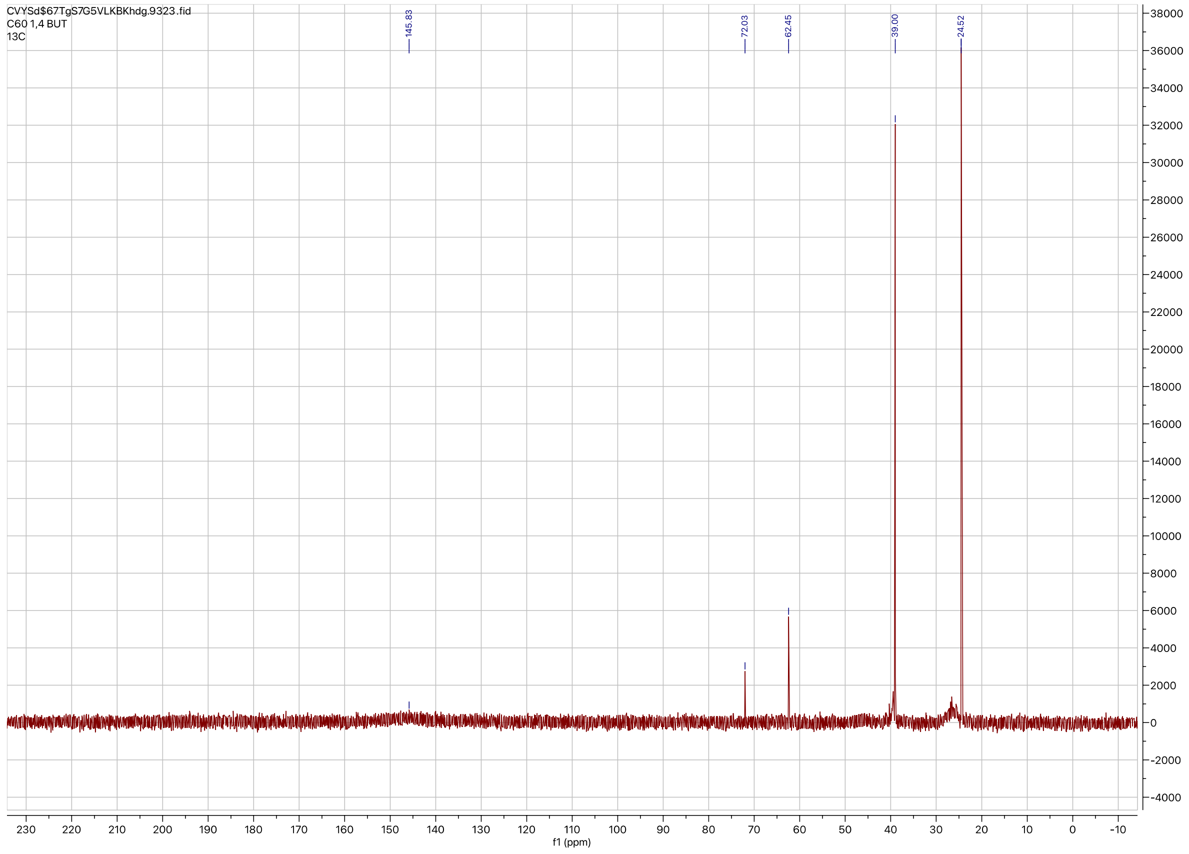

Figure S4

^{13}C-NMR spectrum of C_{60}BUT in D_2O

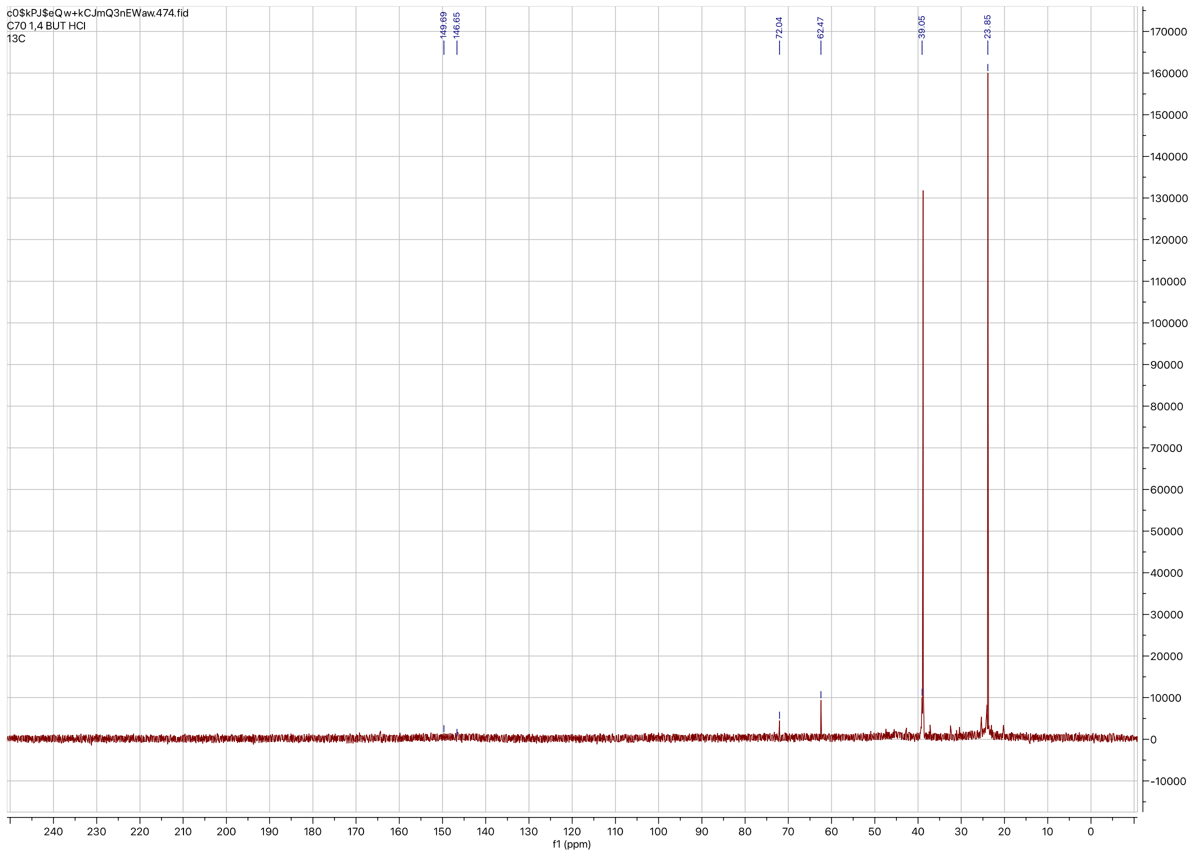

Figure S5

^{13}C-NMR spectrum of C_{70}BUT in D_2O

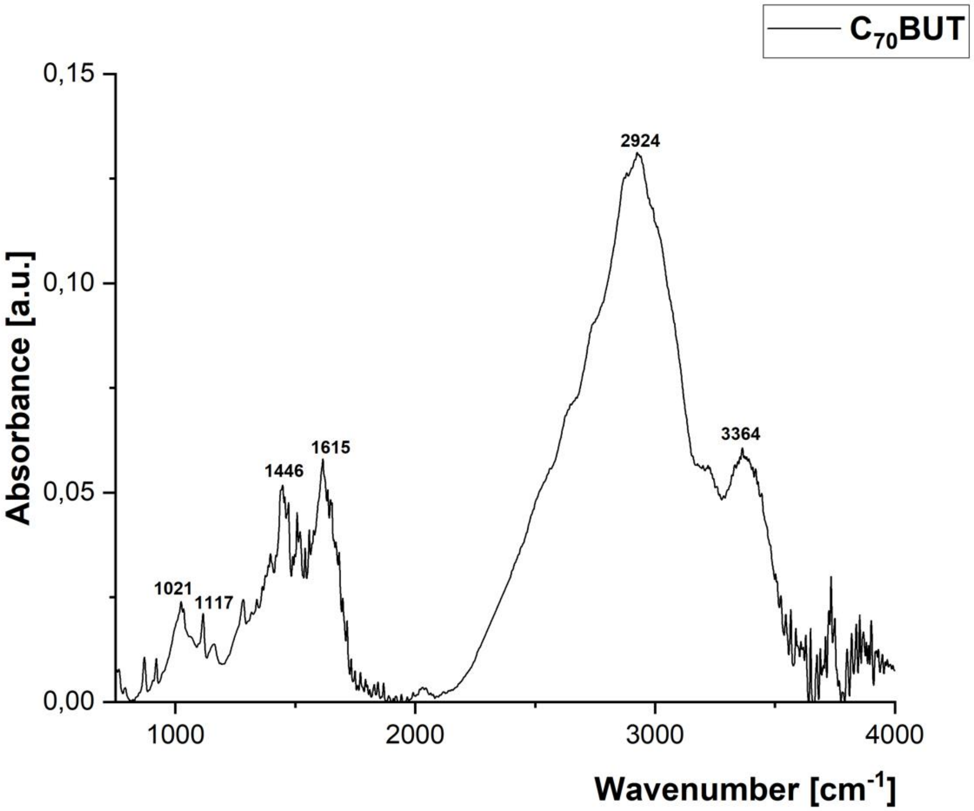

Figure S6

FT-IR spectrum of fullerene nanomaterial C_{70}BUT

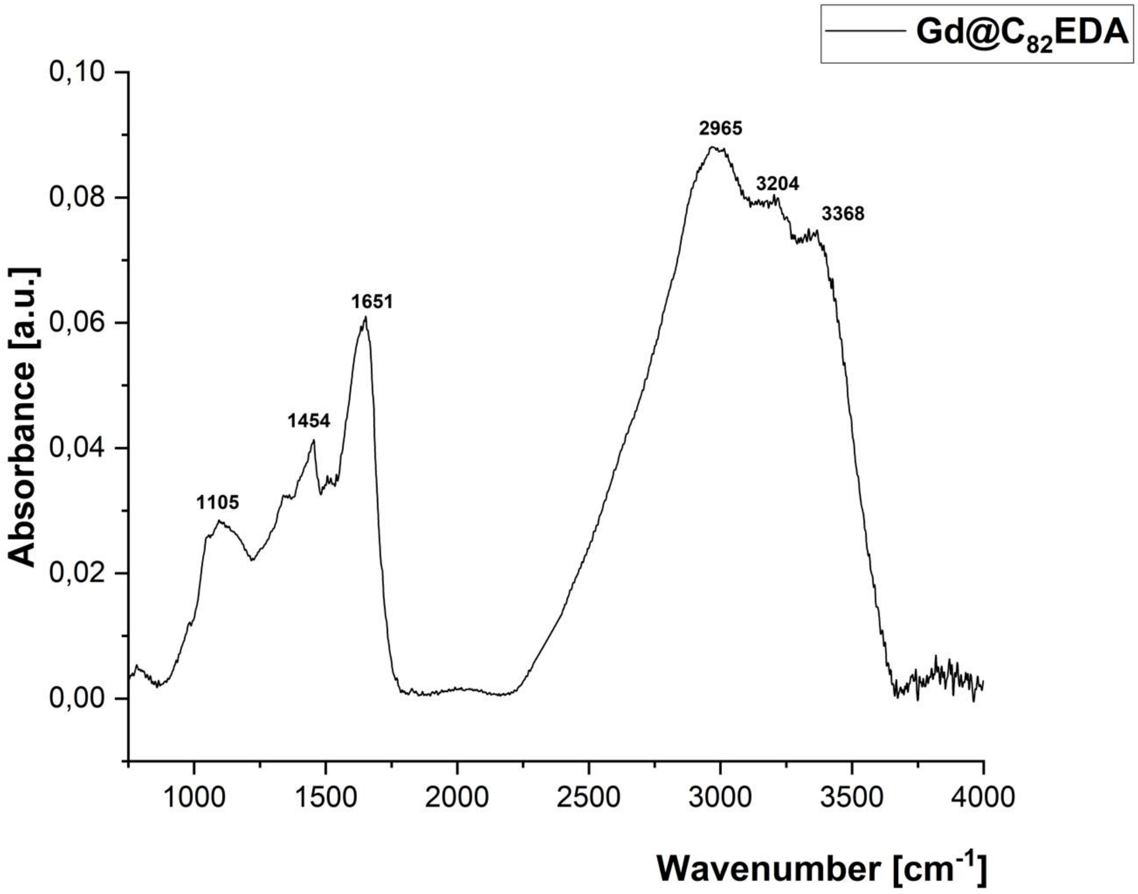

Figure S7

FT-IR spectrum of fullerene nanomaterial Gd@C_{82}EDA.

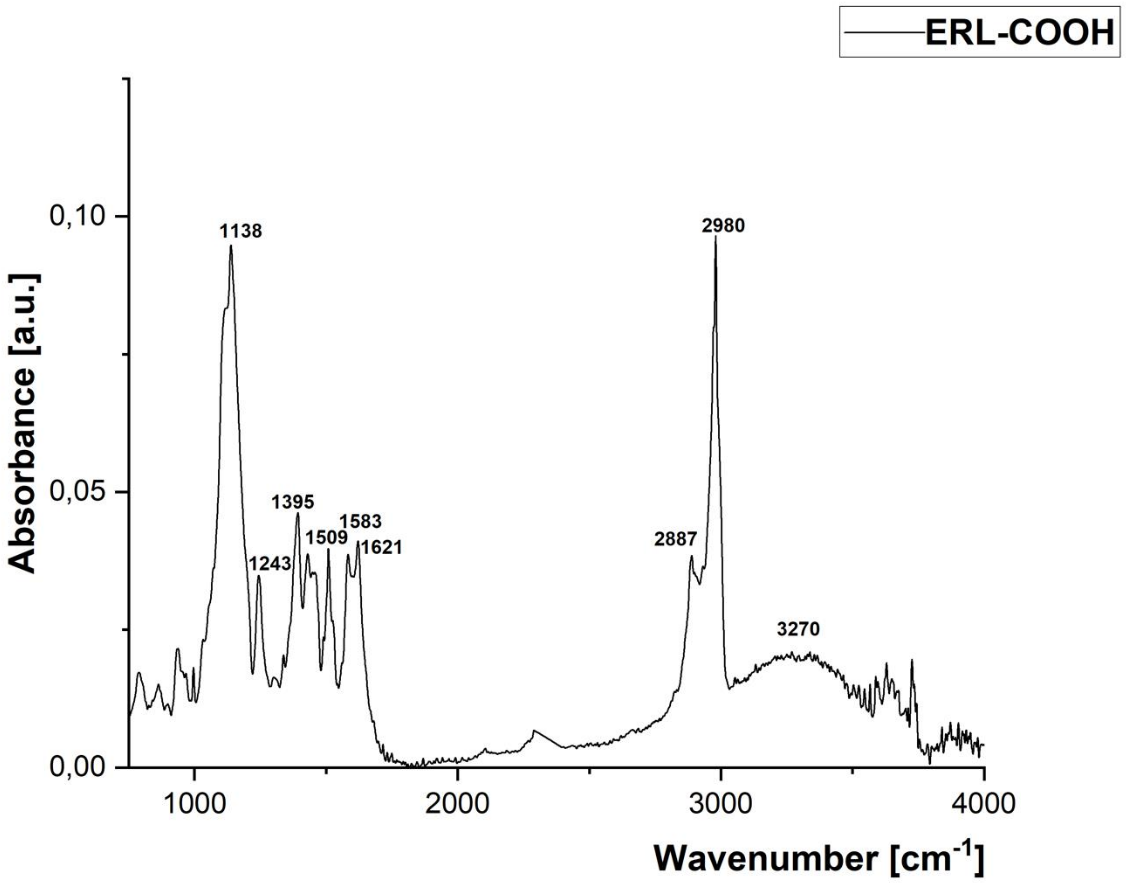

Figure S8

FT-IR spectrum of ERL-COOH *[2-(4-(3-((6,7-bis(2-methoxyethoxy)quinazolin-4-yl)amino)phenyl)-1H-1,2,3-triazol-1-yl)acetic acid]*

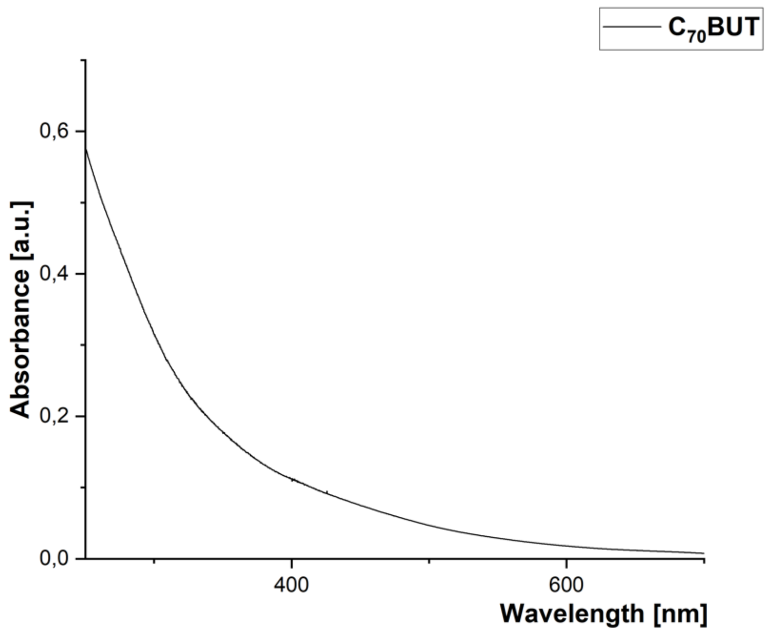

Figure S9

UV-VIS spectrum of C_{70}BUT.

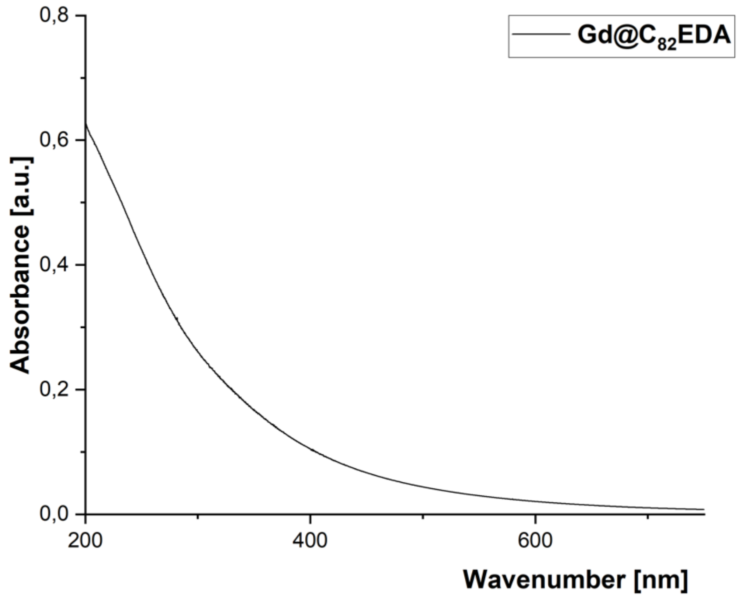

Figure S10

UV-VIS spectrum of Gd@C_{82}EDA.

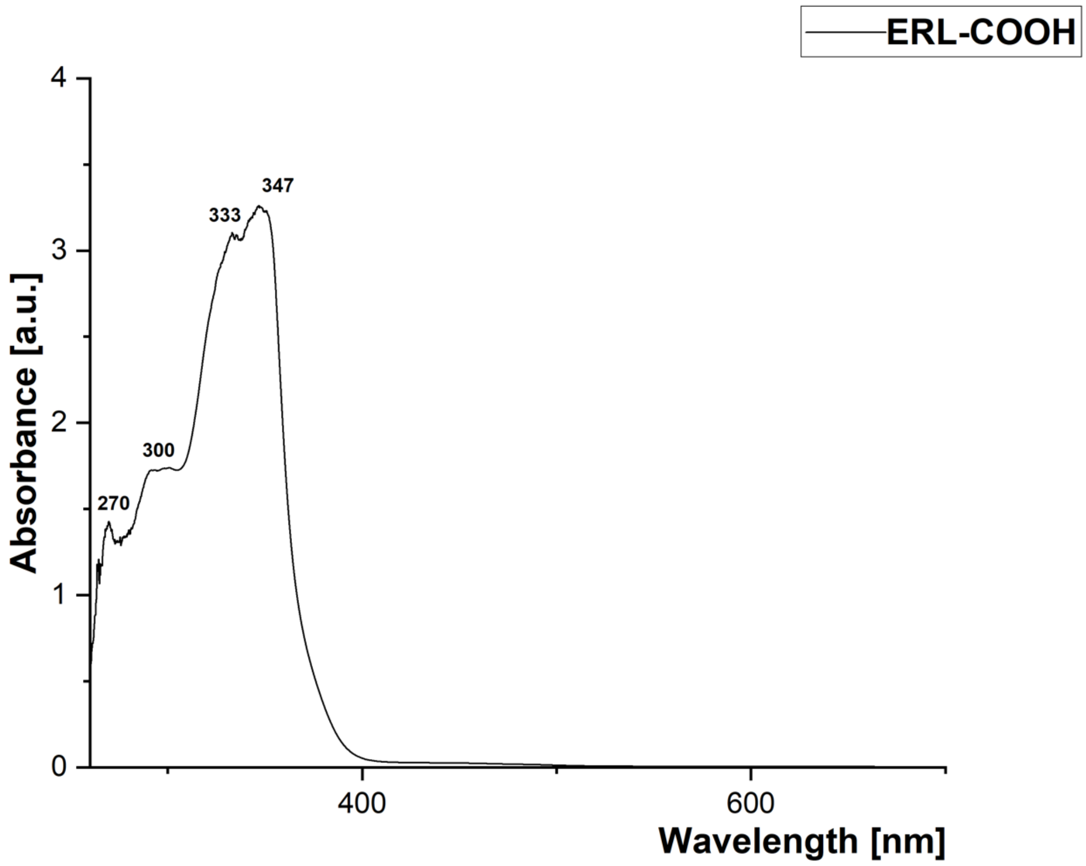

Figure S11

UV-VIS spectrum of ERL-COOH *[2-(4-(3-((6,7-bis(2-methoxyethoxy)quinazolin-4-yl)amino)phenyl)-1H-1,2,3-triazol-1-yl)acetic acid]*

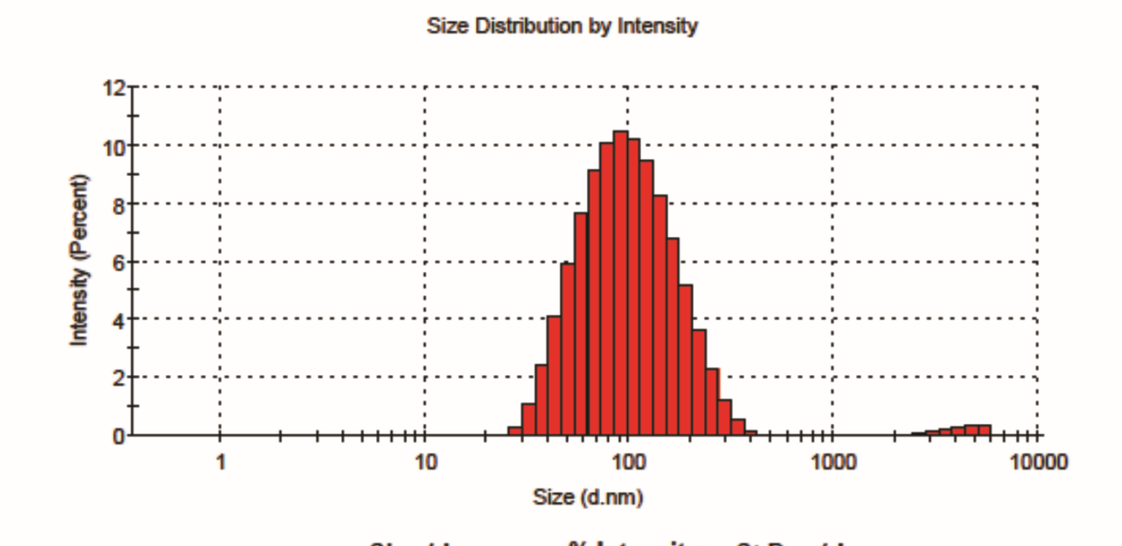

	Size (d.n...	% Intensity:	St Dev (d.n...
Peak 1:	110,6	98,7	59,14
Peak 2:	4413	1,3	915,4

Figure S12

The size of C_{60}BUT nanoparticles measured using DLS technique (c=0.1 mg/mL).

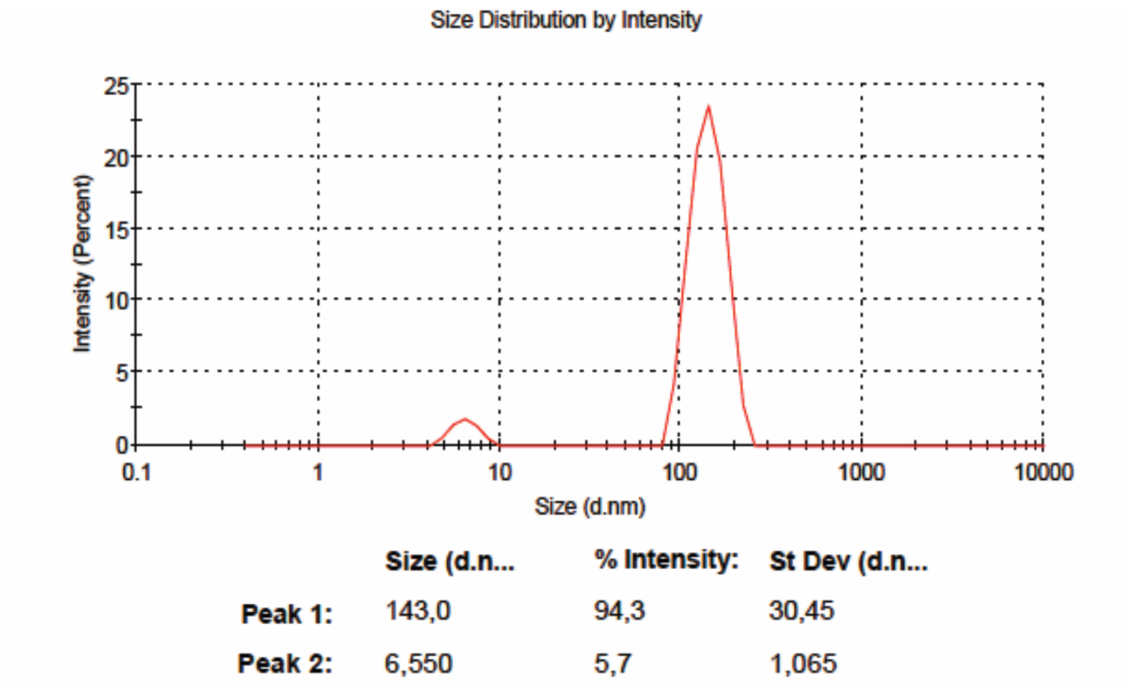

Figure S13

The size of C_{70}BUT-ERL nanoparticles measured using DLS technique (c=0.1 mg/mL).

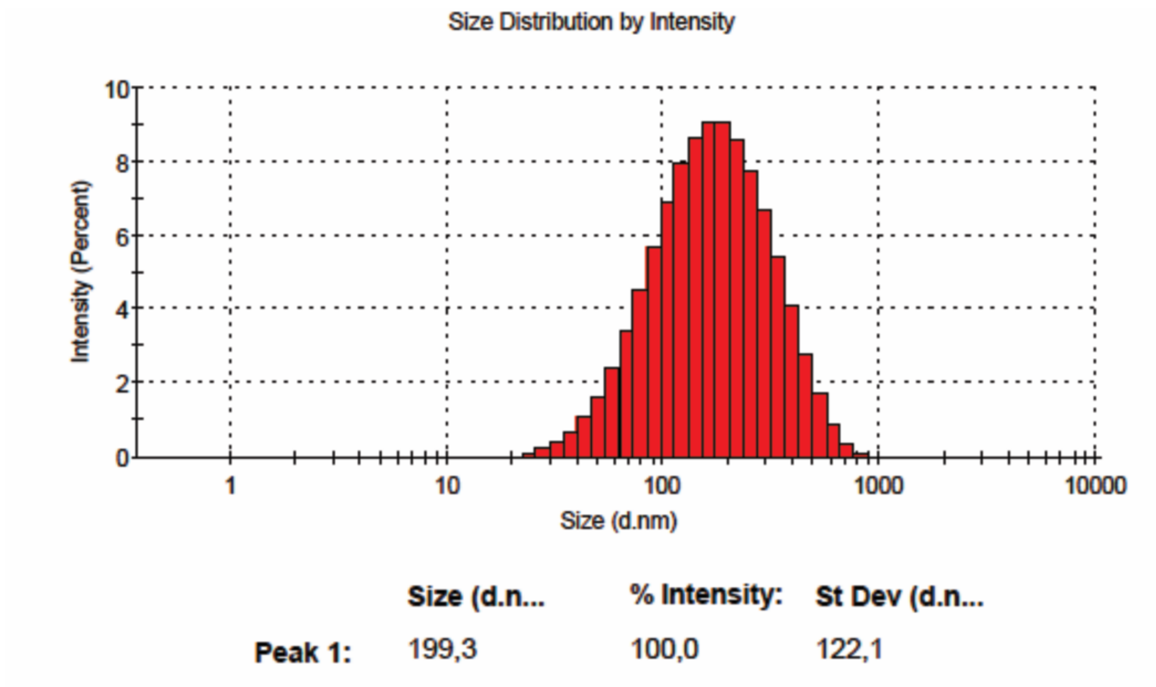

Figure S13

The size of Gd@C_{82}EDA-ERL nanoparticles measured using DLS technique (c=0.1 mg/mL).

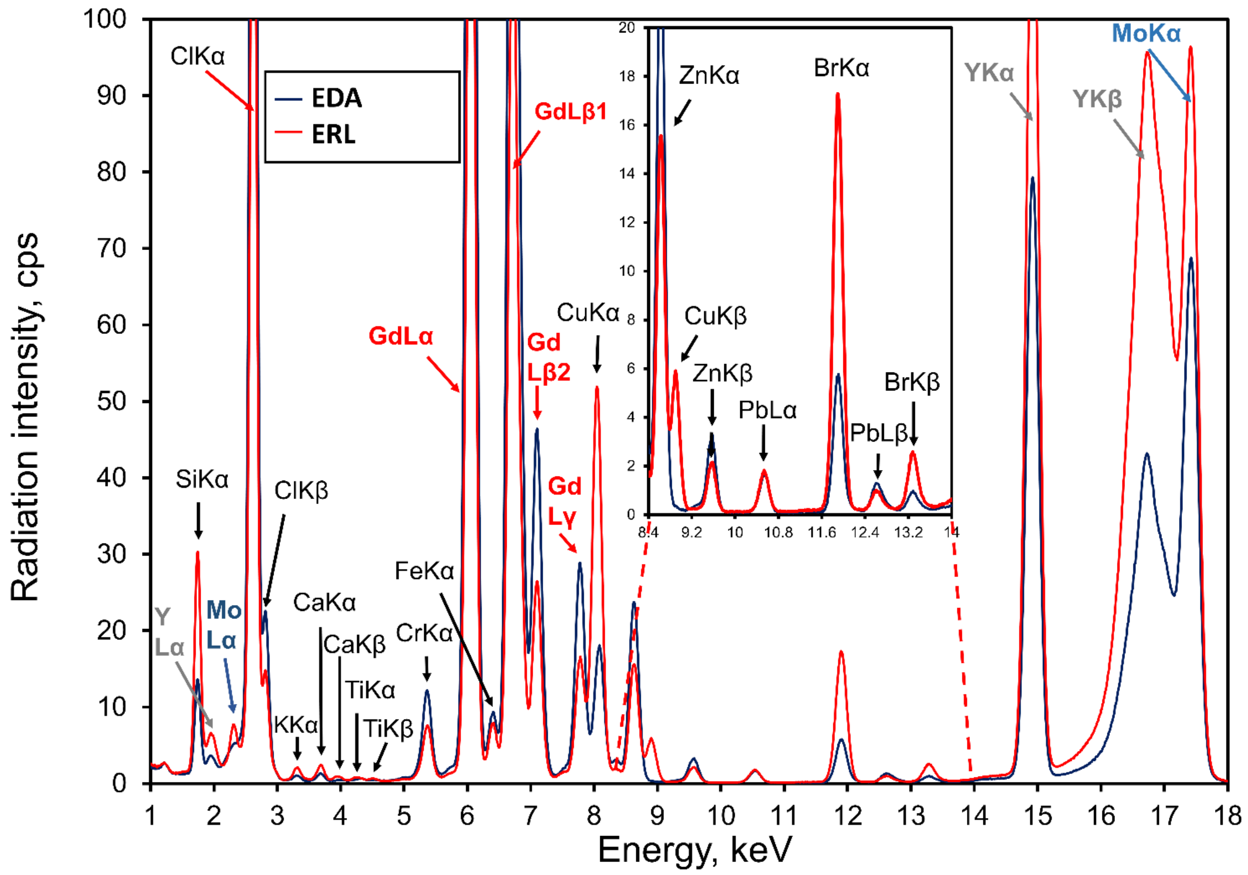

Figure S14

TXRF spectra of Gd@C_{82}EDA (blue line), and Gd@C_{82}EDA-ERL (red line) suspensions (50 kV, 1000 µA, ambient air atmosphere, 1000 s counting time).

C_{60}BUT

**Element**	**Concentration**
C	75.84
N	6.72
H	17.44

C_{70}BUT

**Element**	**Concentration**
C	88.41
N	7.14
H	4.45

Gd@C_{82}EDA

**Element**	**Concentration**
C	62.12
N	11.41
H	3.92

- $C_{60}(NHCH_2CH_2CH_2CH_2NH_2)_x$; *N/C* ratio= $\frac{28X}{720+48X}$= $\frac{6.72}{75.84}$; x= 3
- $C_{70}(NHCH_2CH_2CH_2CH_2NH_2)_x$; *N/C* ratio =$\frac{28X}{840+48X}$= $\frac{7.14}{88.41}$; x= 3
- Gd@$C_{82}(NHCH_2CH_2NH_2)_x$; *N/C* ratio =$\frac{28X}{984+24X}$= $\frac{11.41}{62.12}$; x= 8
-

Table S1

Elemental analysis of synthesized aminofullerenes (*C/N/H*).

Element	$Gd@C_{82}EDA$		$Gd@C_{82}EDA$ ERL	
	Concentration, μg mL^{-1}	SD	Concentration, μg mL^{-1}	SD, μg mL^{-1}
Chlorine	242	1	83.1	0.2
Potassium	0.62	0.01	0.23	0.01
Calcium	0.38	0.01	0.361	0.004
Titanium	0.012	0.001	0.021	0.001
Iron	0.342	0.002	0.254	0.002
Copper	0.066	0.001	0.958	0.003
Zinc	0.152	0.001	0.086	0.001
Bromine	0.010	0.001	0.075	0.001
Lead	0.029	0.001	0.009	0.001
**Gadolinium**	**13.76**	**0.03**	**9.19**	**0.02**

Table S2

The chemical composition of **$Gd@C_{82}EDA$** and **$Gd@C_{82}EDA$-ERL** determined with TXRF spectrometry.

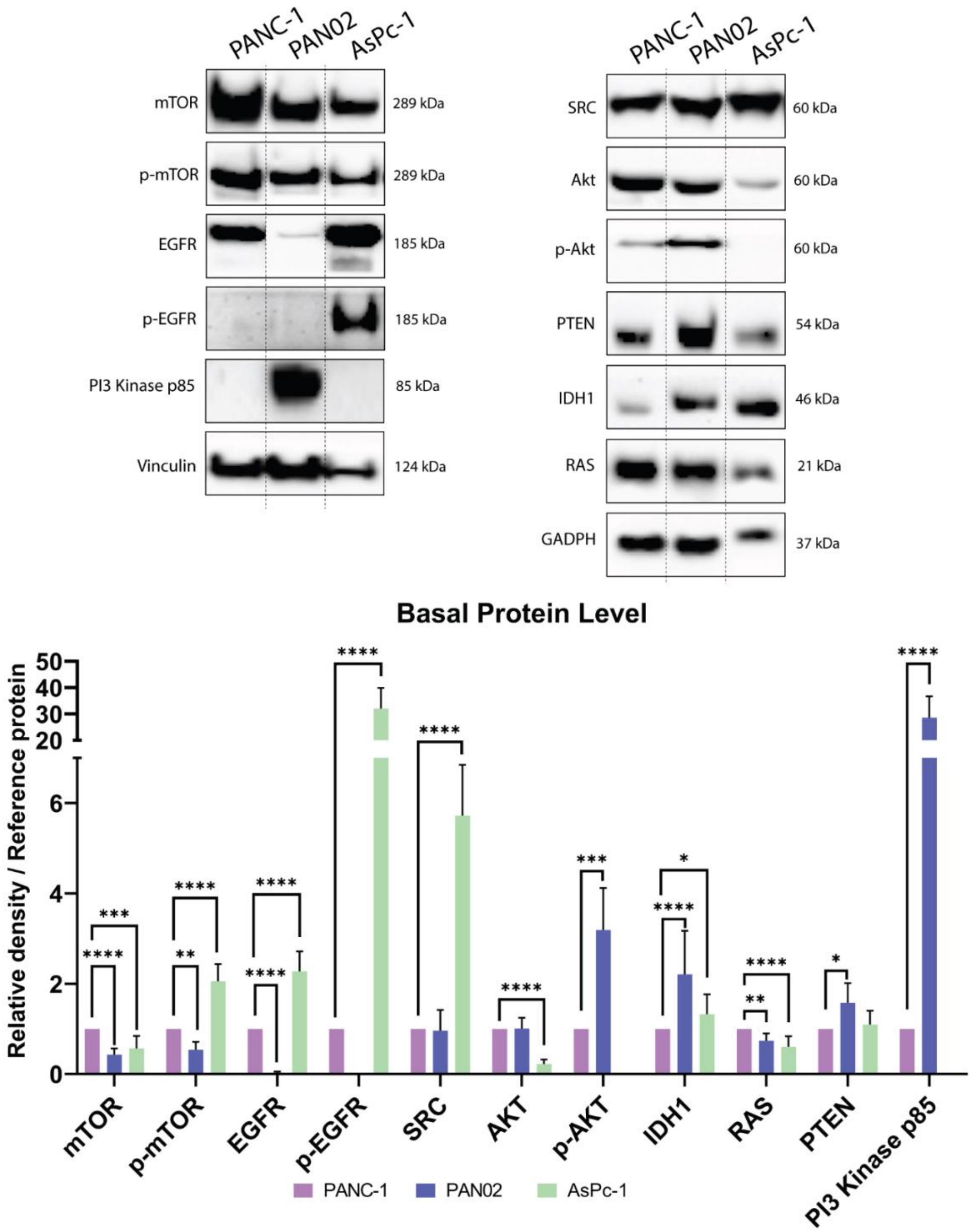

Figure S15

The landscape of basal protein expression levels associated with EGFR signalling pathway in pancreatic cell lines: PANC-1, PAN02, and AsPC-1. The protein levels are presented in relation to PANC-1 cells. The statistic was done using one-way ANOVA with Šídák's multiple comparison with use of GraphPad 9.0.

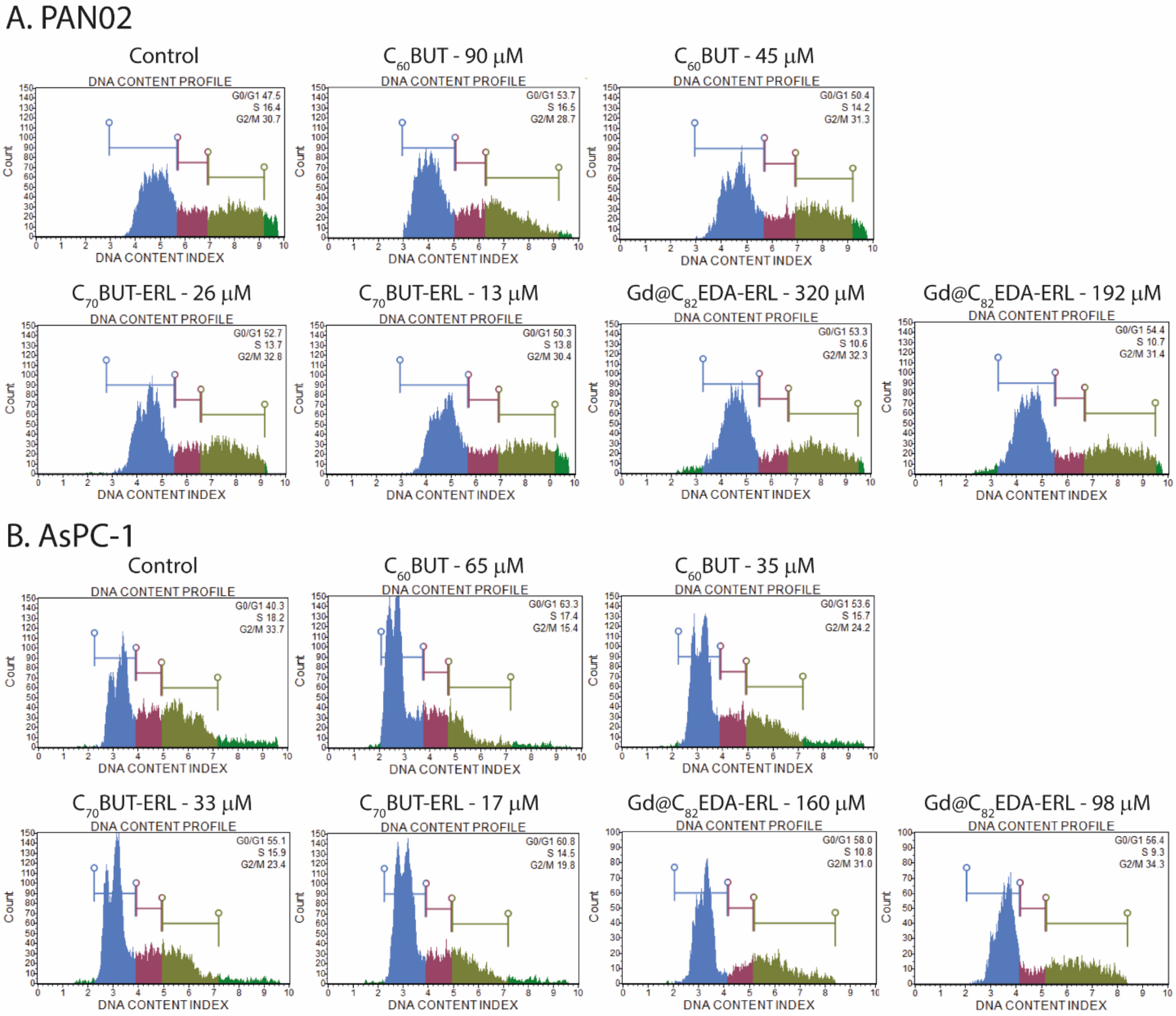

Figure S16

Representative histograms from cell cycle studies using flow cytometry: (A) PAN02 and (B) AsPC-1 cells.

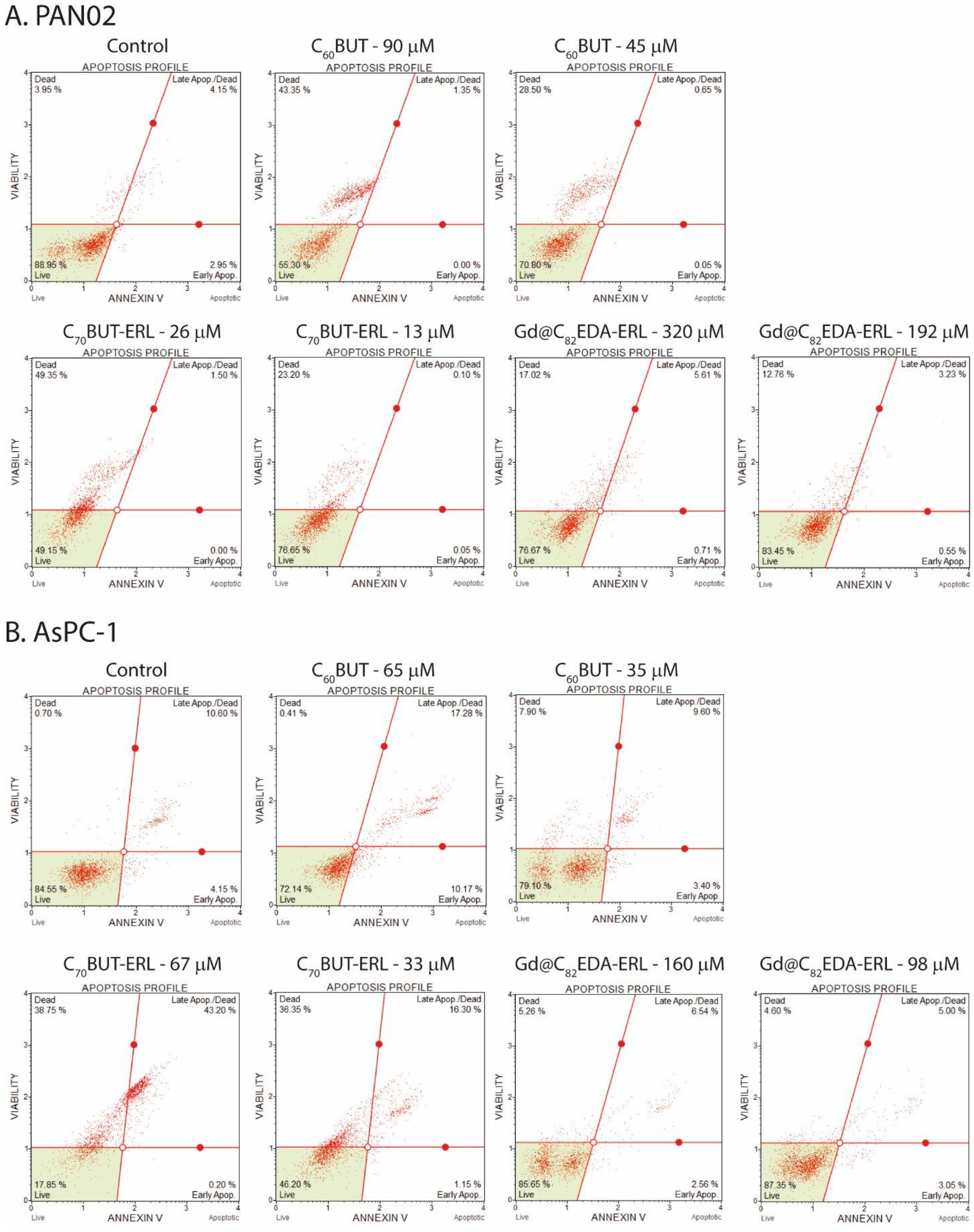

Figure S17 Representative histograms from apoptosis studies using flow cytometry: (A) PAN02 and (B) AsPC-1 cells.

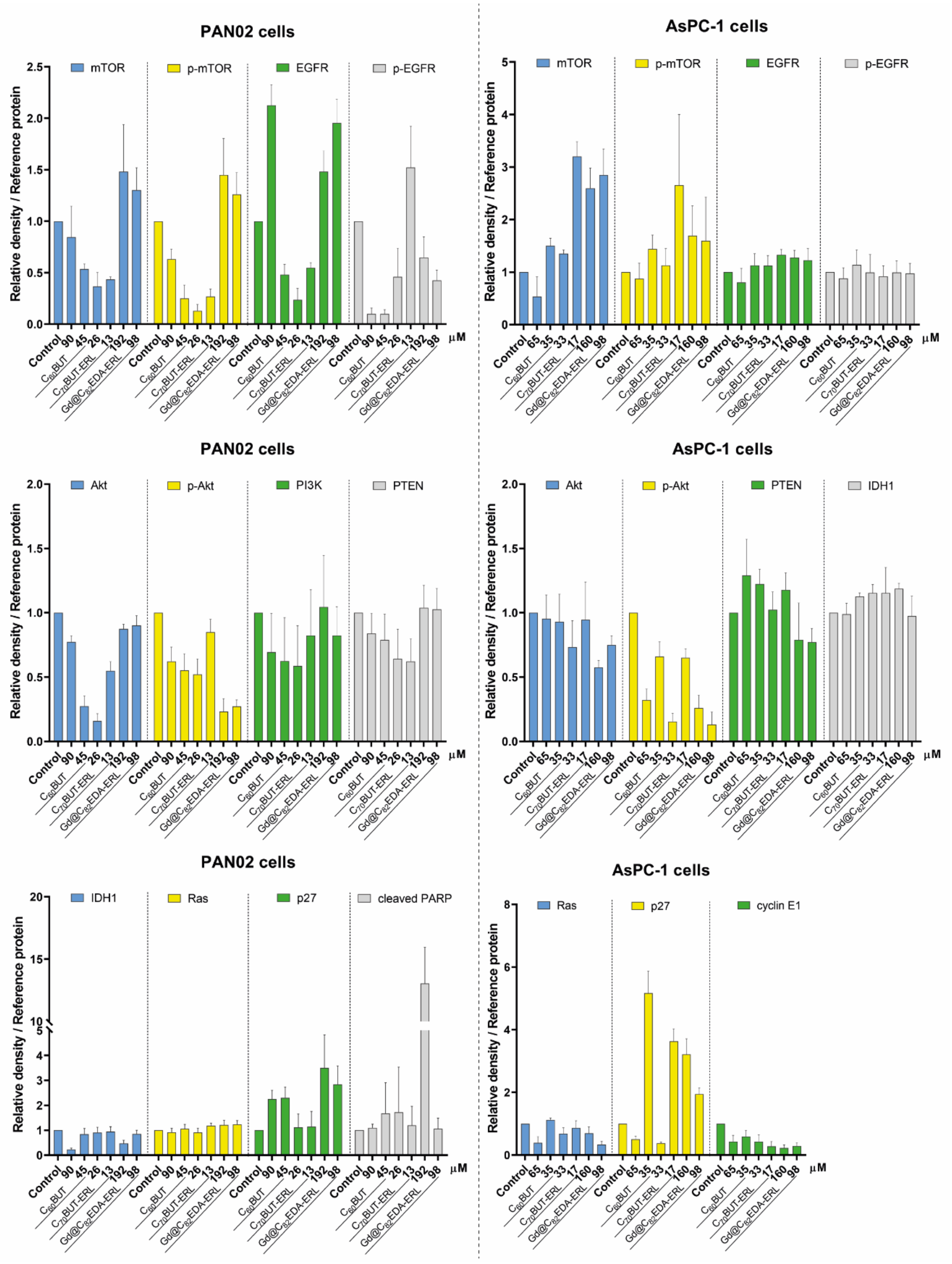

Figure S18

The densitometric analysis charts of the expression of tested proteins after treatment with nanomaterials. Results were normalized to the reference protein and are from four independent experiments.

Images of the gels prepared during this study

A. *PAN02 cells (Fig. 7 in main text); exposure: 39.8sec*

We used prestained molecular weight ladder: Novex Sharp Pre-stained Protein Standard (#LC5800, Thermo Scientific).

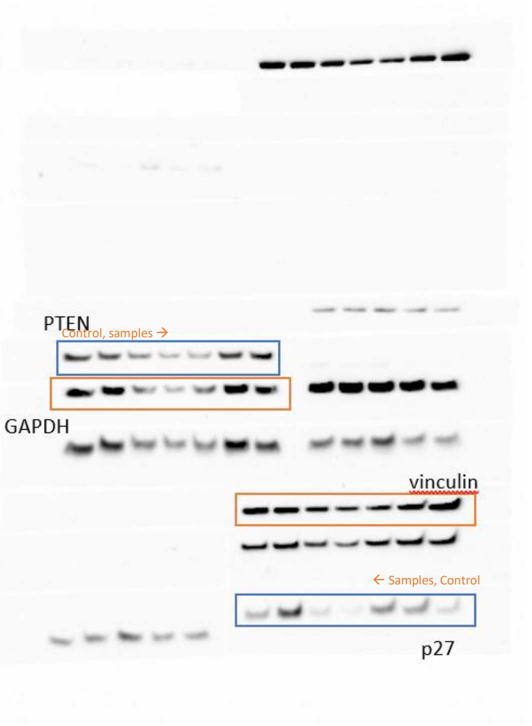

Gel with a molecular weight marker in CCD camera

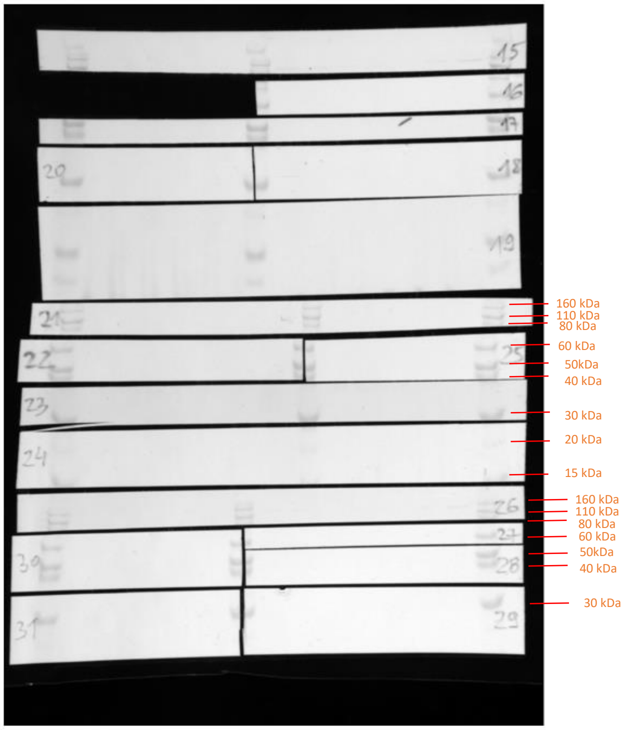

Merged images above

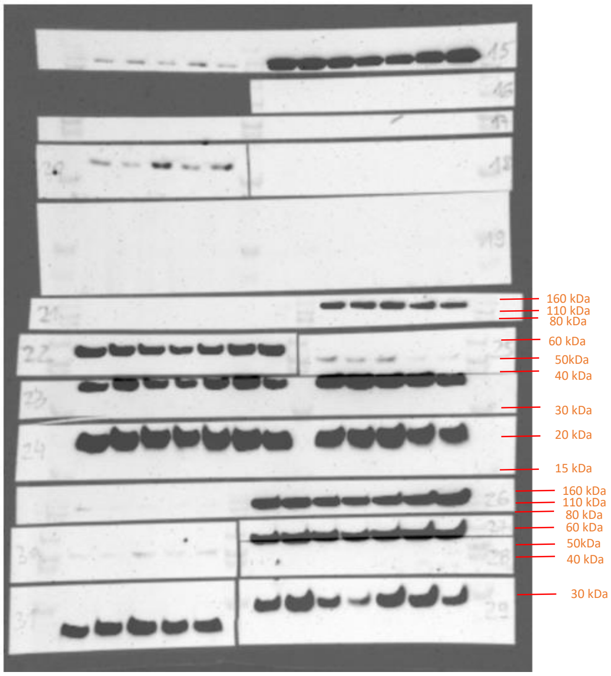

B. *PAN02 cells (Fig. 7 in main text); exposure 39.8sec*

We used prestained molecular weight ladder: Novex Sharp Pre-stained Protein Standard (#LC5800).

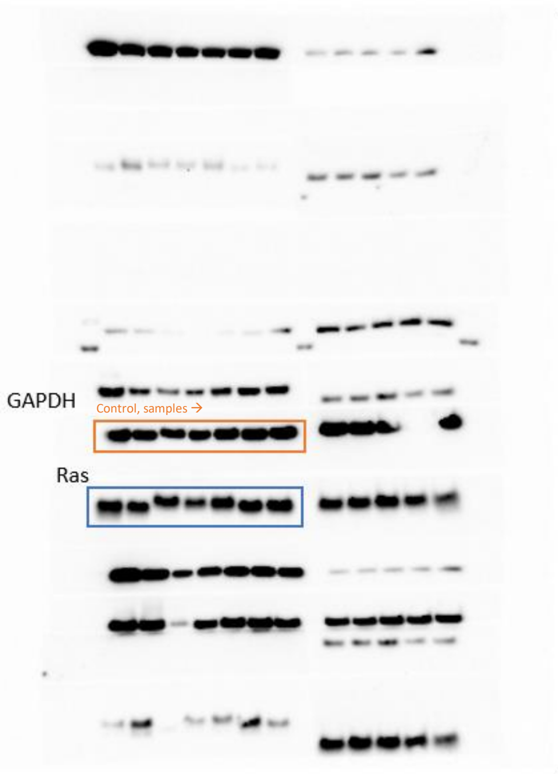

Gel with a molecular weight marker in CCD camera

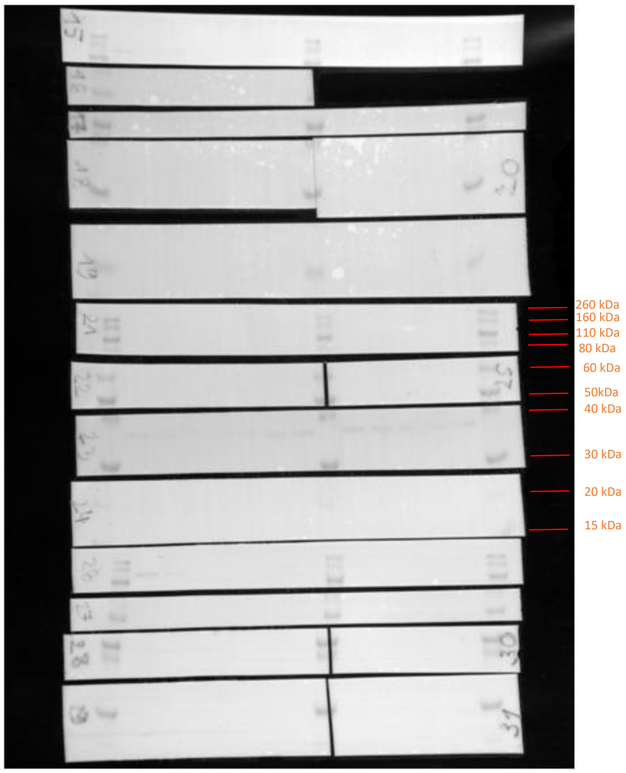

Merged images above

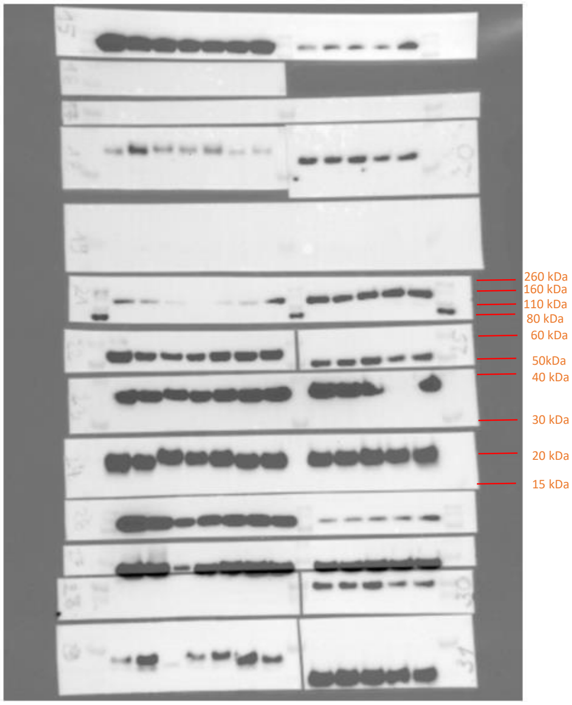

C. *PAN02 cells (Fig. 7 in main text); exposure 10.0sec + 39.8sec + 248.0 sec*

We used prestained molecular weight ladder: Spectra Multicolor High Range Protein Ladder (#26625, Thermo Scientific) and Novex Sharp Pre-stained Protein Standard (#LC5800).

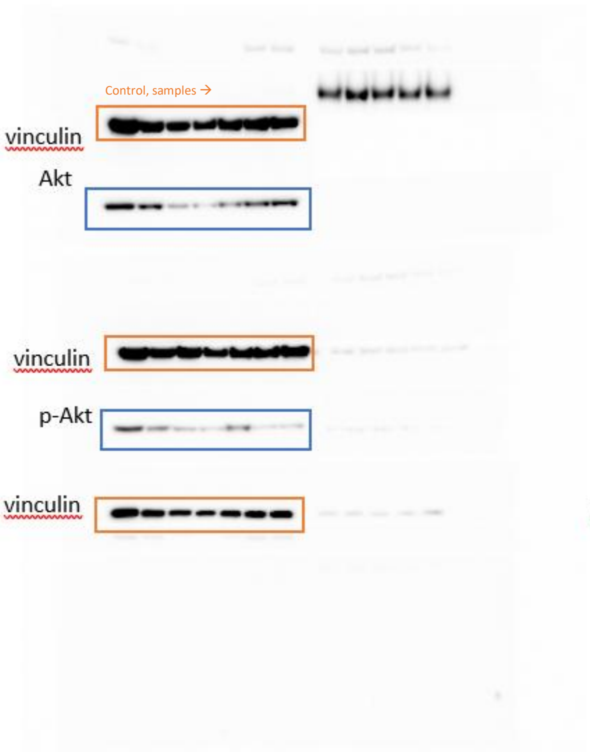

longer exposure

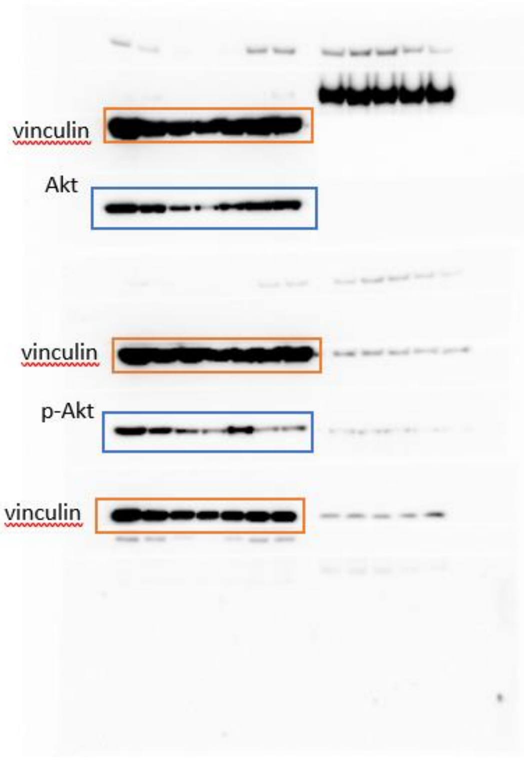

longer exposure

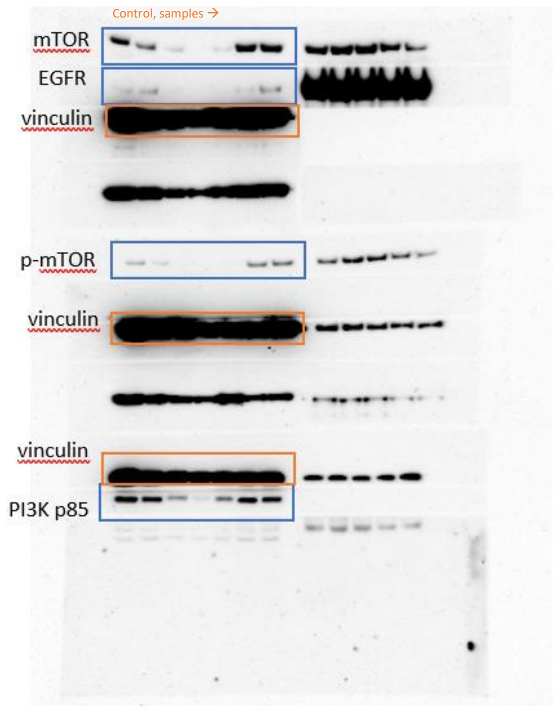

Gel with a molecular weight marker in CCD camera

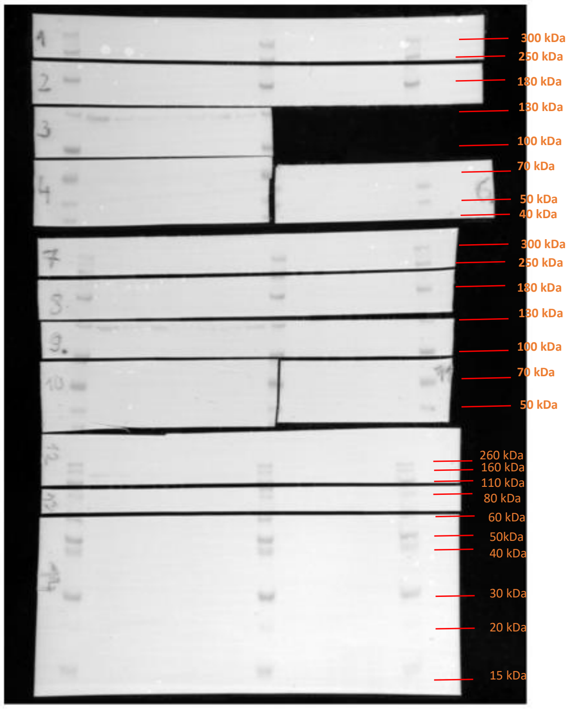

Merged images above

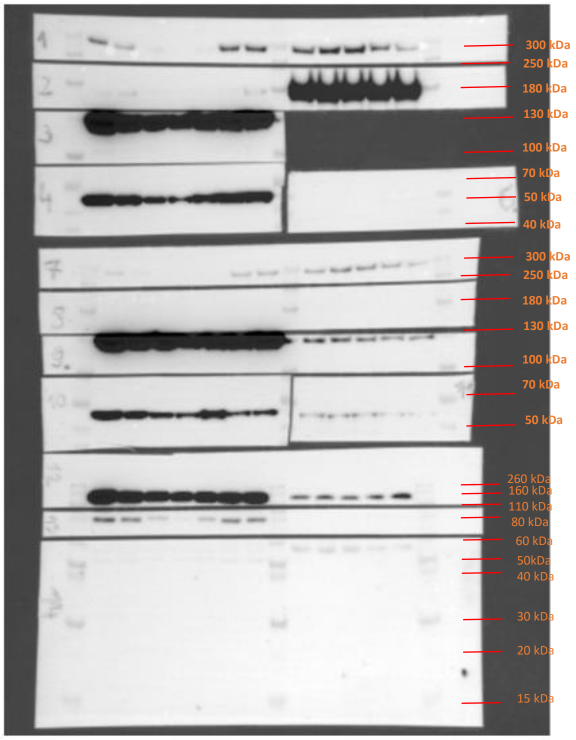

D. PAN02 cells (Fig. 7 in main text) – exposure 813.2sec

We used prestained molecular weight ladder: Spectra Multicolor High Range Protein Ladder (#26625).

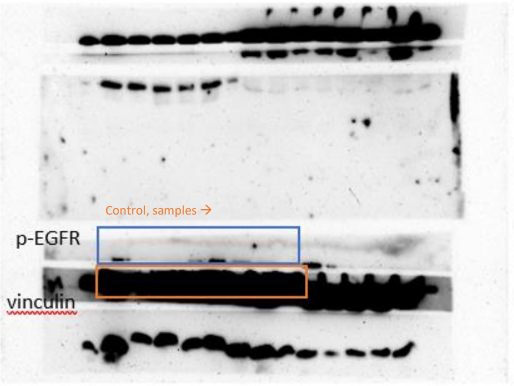

Gel with a molecular weight marker in CCD camera

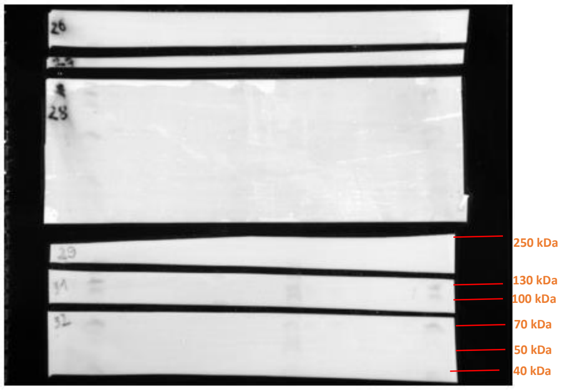

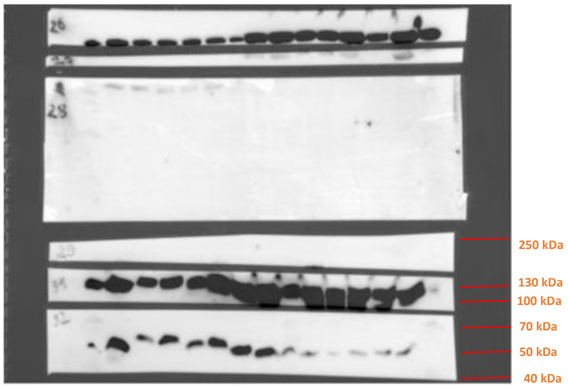

Merged images above

E. PAN02 cells (Fig. 7 in main text) – exposure 724.0sec

We used prestained molecular weight ladder: Novex Sharp Pre-stained Protein Standard (#LC5800).

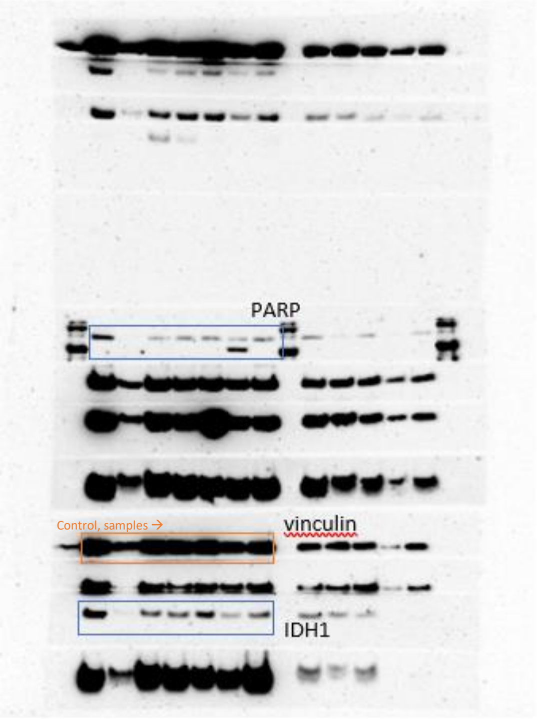

Gel with a molecular weight marker in CCD camera

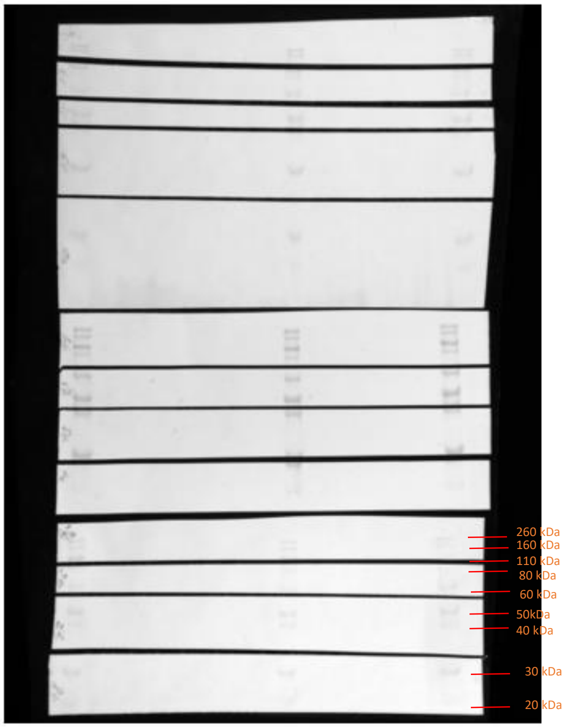

Merged images above

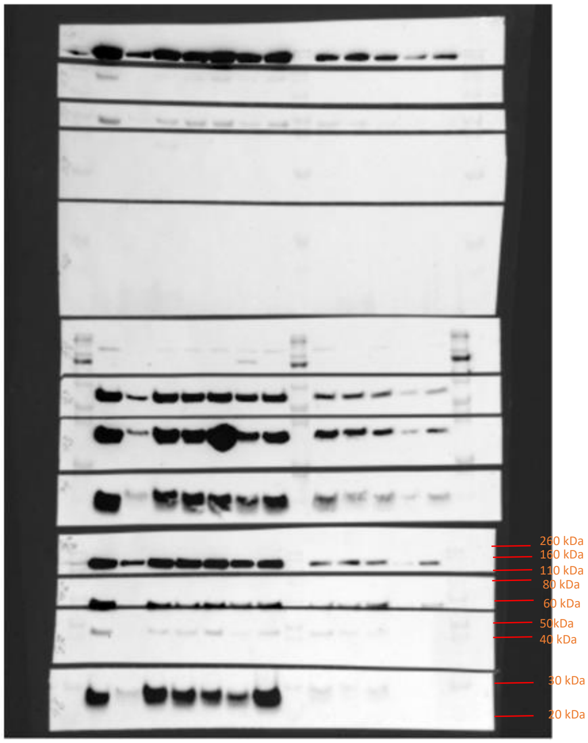

F. *AsPC-1 cells (Fig. 7 in main text) – exposure 337.2sec + 813.2sec*

We used prestained molecular weight ladder: Spectra Multicolor High Range Protein Ladder (#26625).

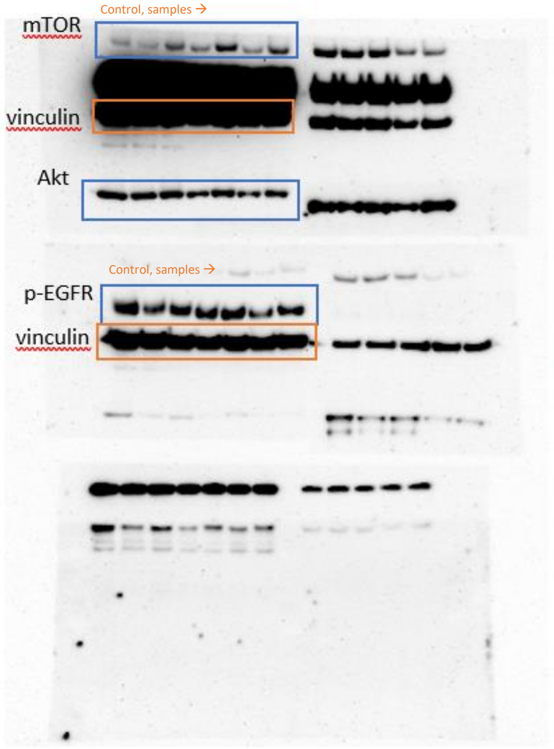

longer exposure

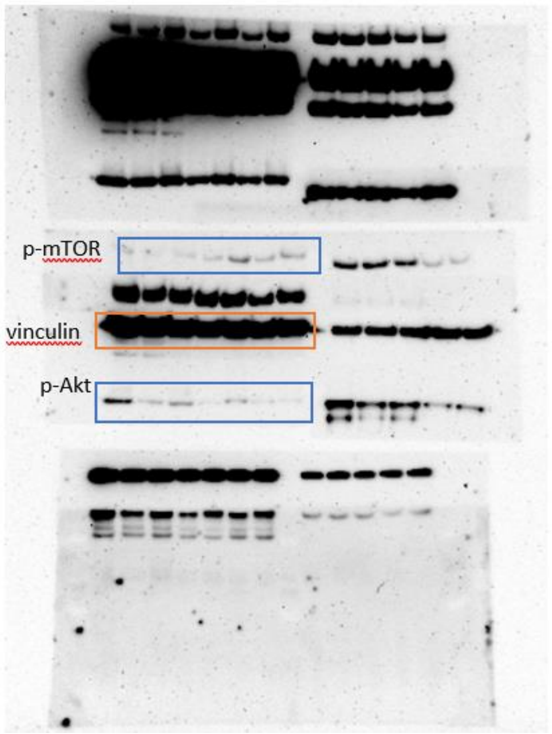

Gel with a molecular weight marker in CCD camera

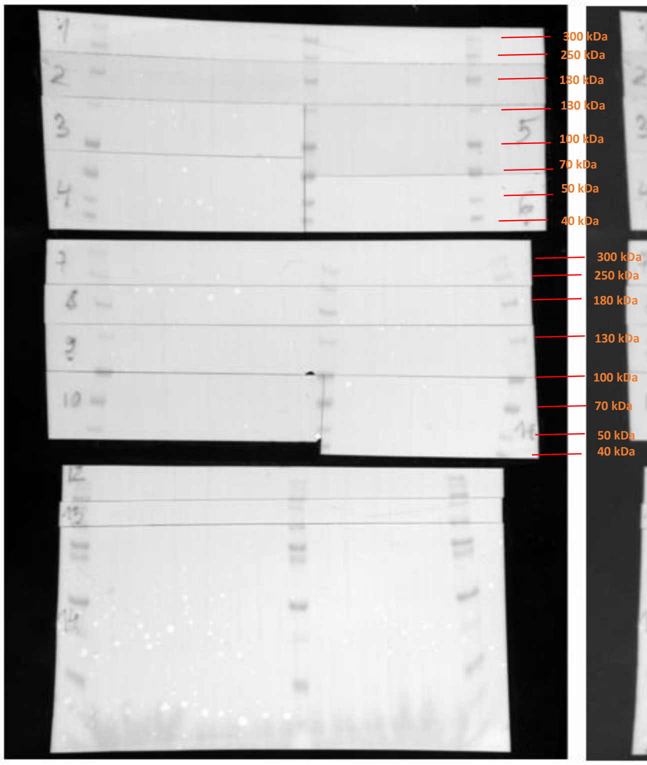

Merged images

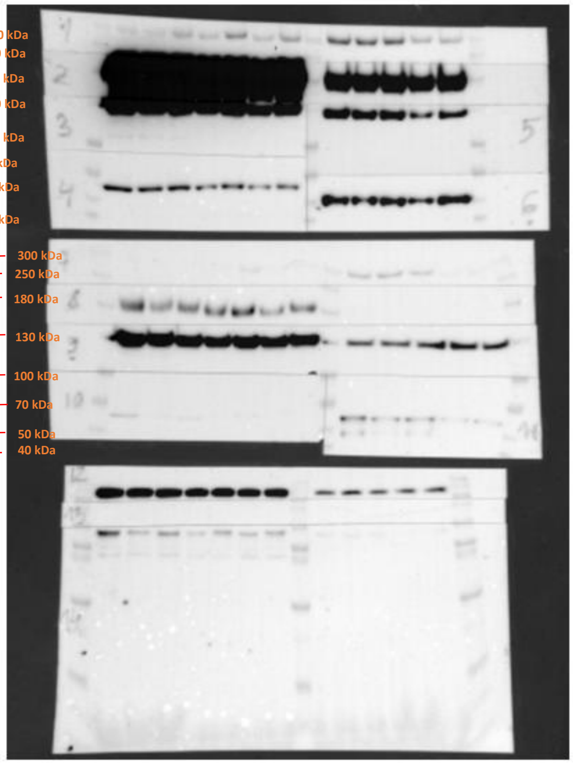

G. *AsPC-1 cells (Fig. 7 in main text) – exposure 456.2sec*

We used prestained molecular weight ladder: Novex Sharp Pre-stained Protein Standard (#LC5800).

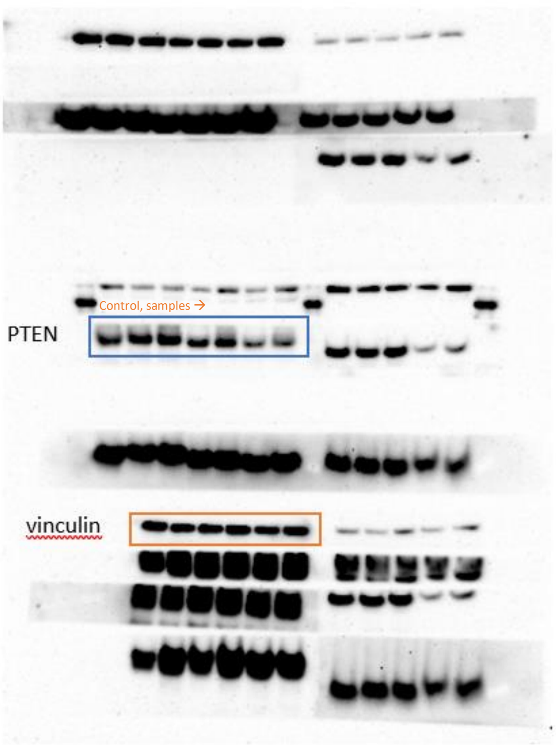

Gel with a molecular weight marker in CCD camera

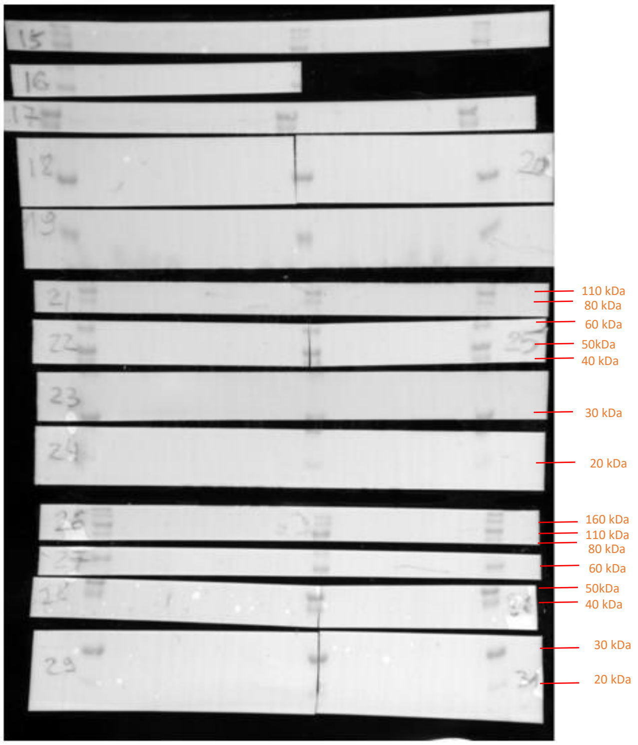

Merged images above

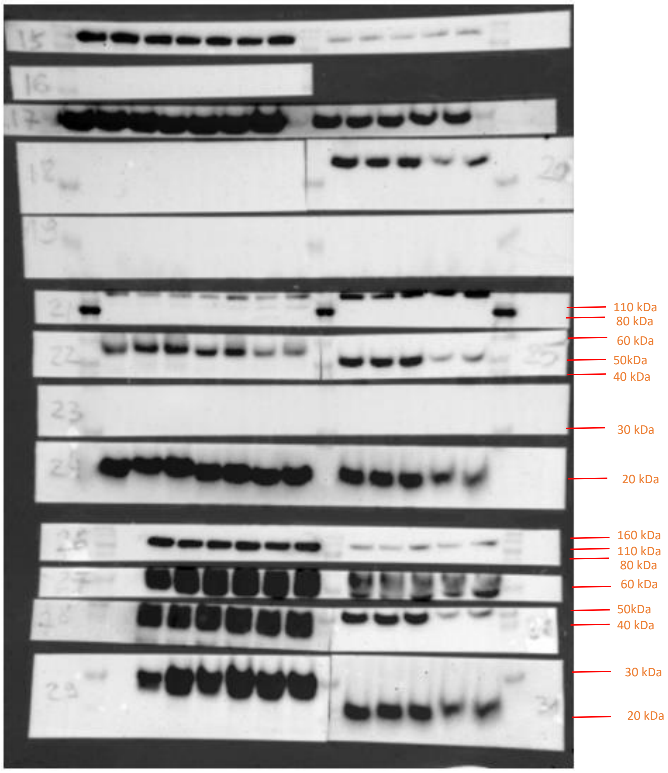

H. *AsPC-1 cells (Fig. 7 in main text) - 99.2sec*

We used prestained molecular weight ladder: Spectra Multicolor High Range Protein Ladder (#26625).

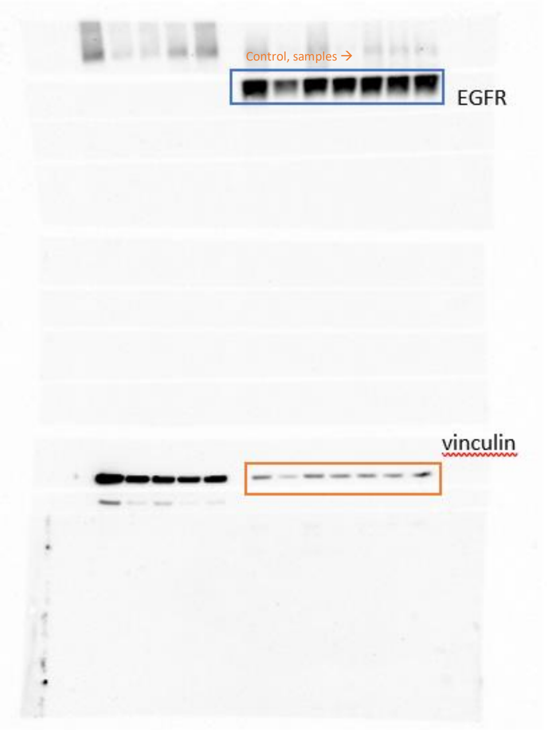

Gel with a molecular weight marker in CCD camera

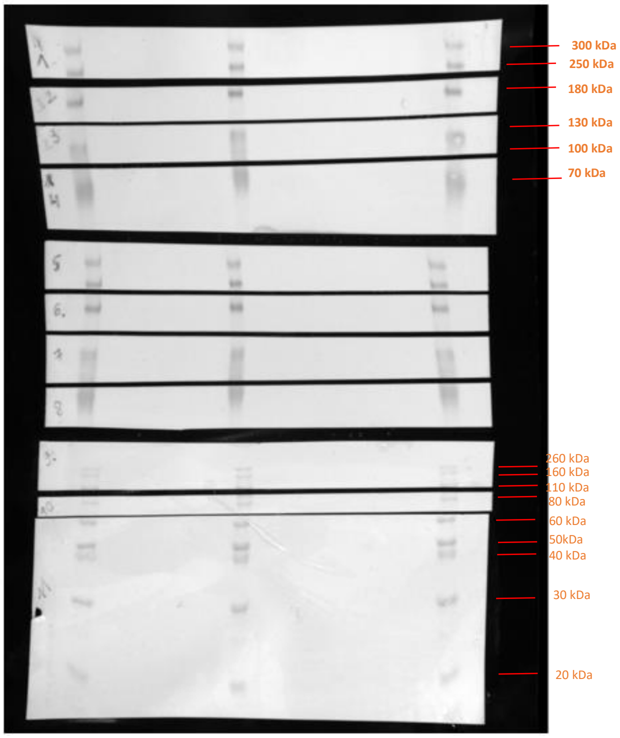

Merged images above

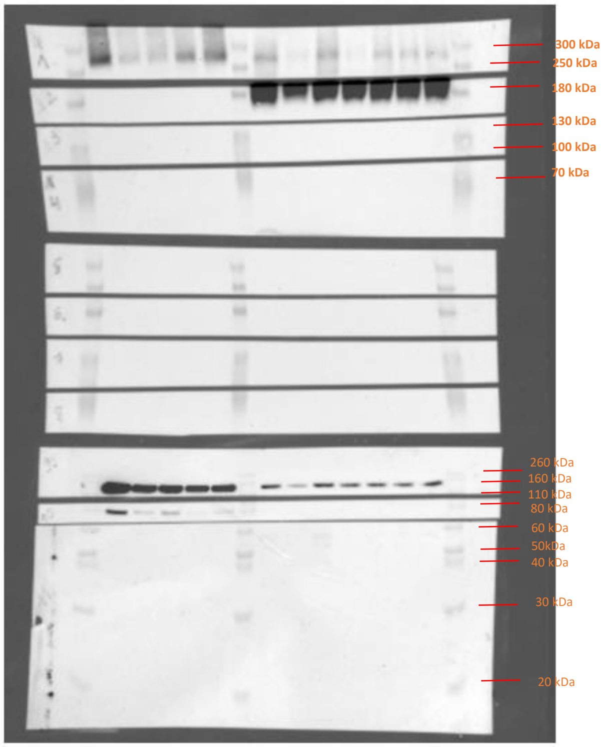

I. AsPC-1 cells (Fig. 7 in main text) – exposure 29.0sec + 337.2sec + 1051.2sec

We used prestained molecular weight ladder: Novex Sharp Pre-stained Protein Standard (#LC5800).

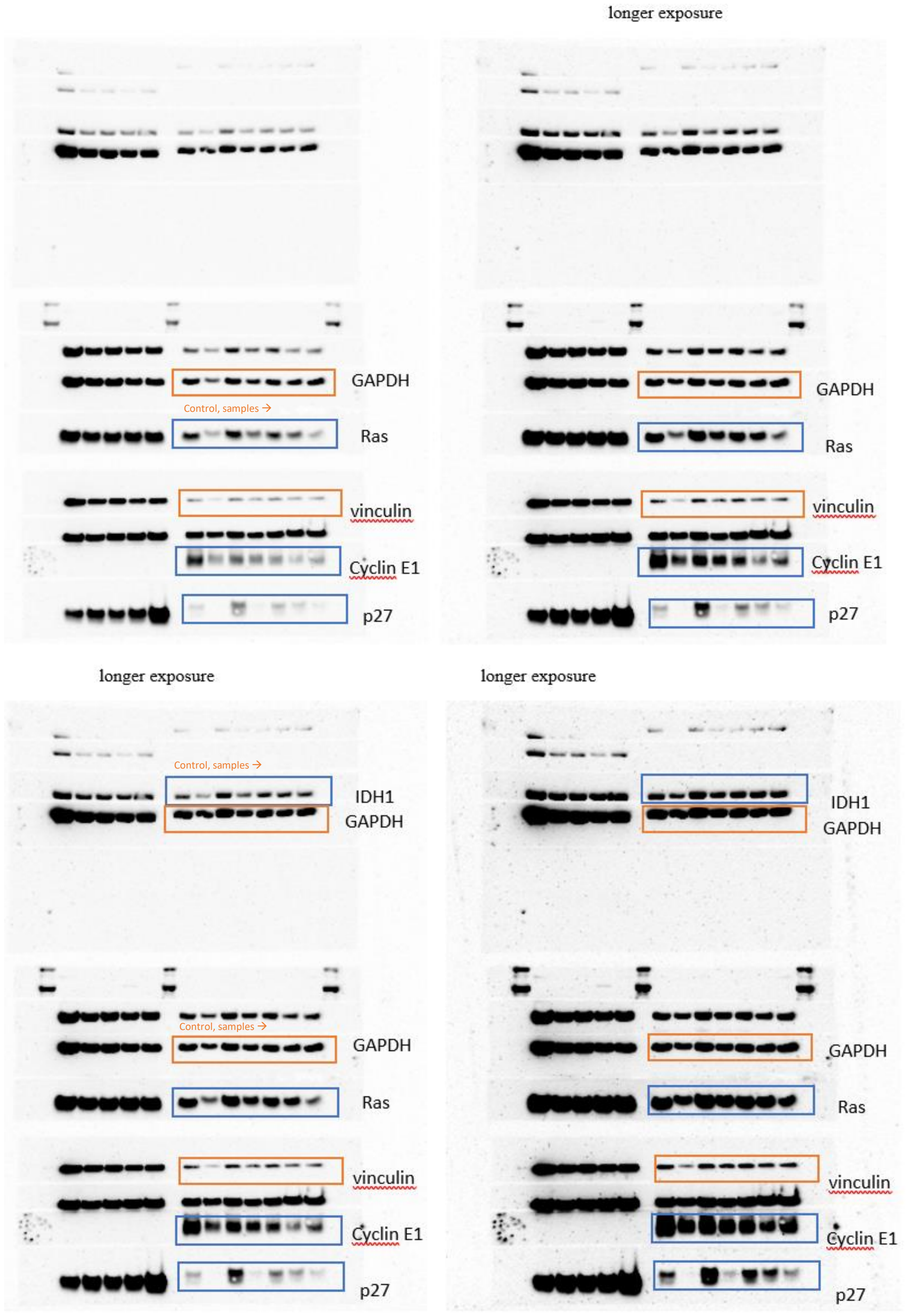

Gel with a molecular weight marker in CCD camera

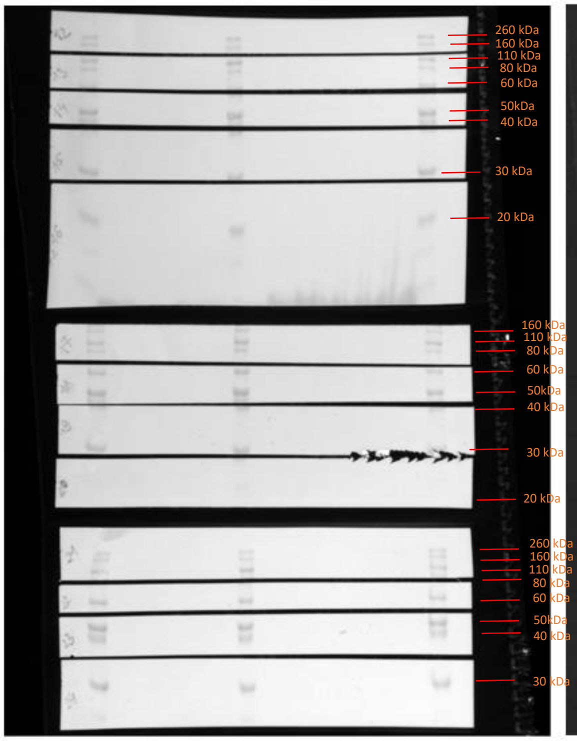

Merged images above

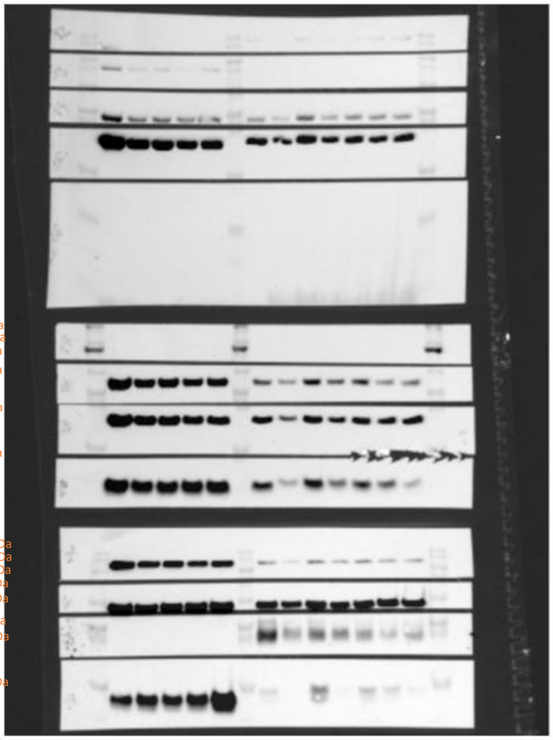

J. *Basal expression (Fig. S15) – exposure 30.0 sec*

We used prestained molecular weight ladder: Spectra Multicolor High Range Protein Ladder (#26625).

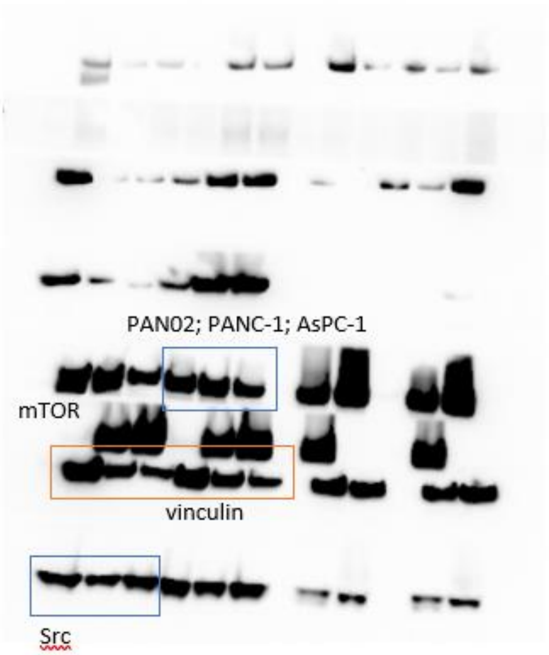

Gel with a molecular weight marker in CCD camera

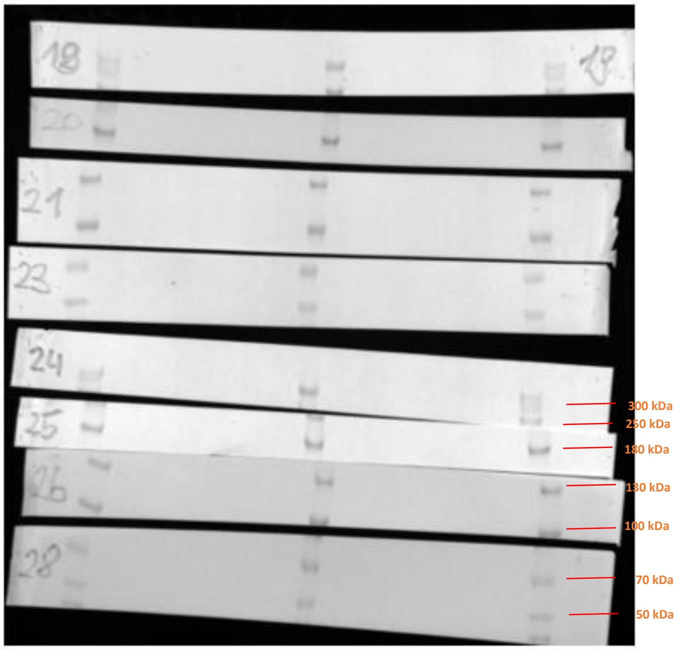

Merged images above

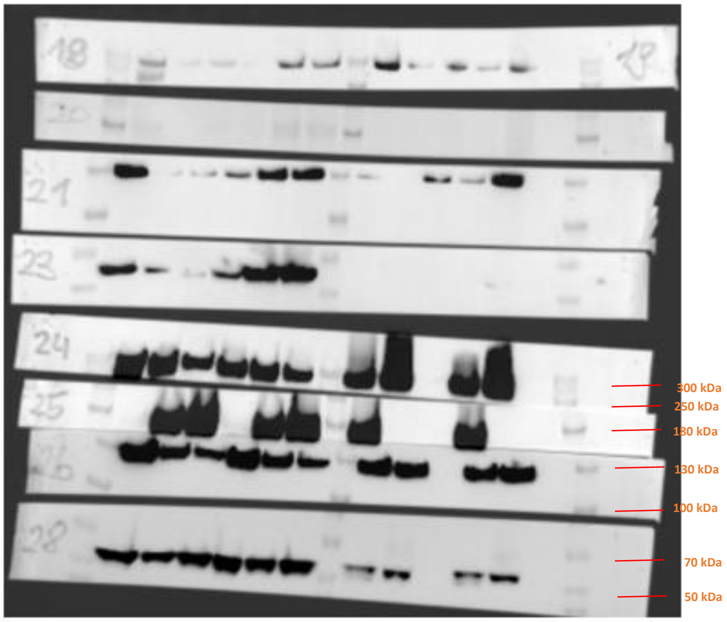

K. Basal expression (Fig. S15) – exposure 30.0 sec + 120.0 sec

We used prestained molecular weight ladder: Novex Sharp Pre-stained Protein Standard (#LC5800).

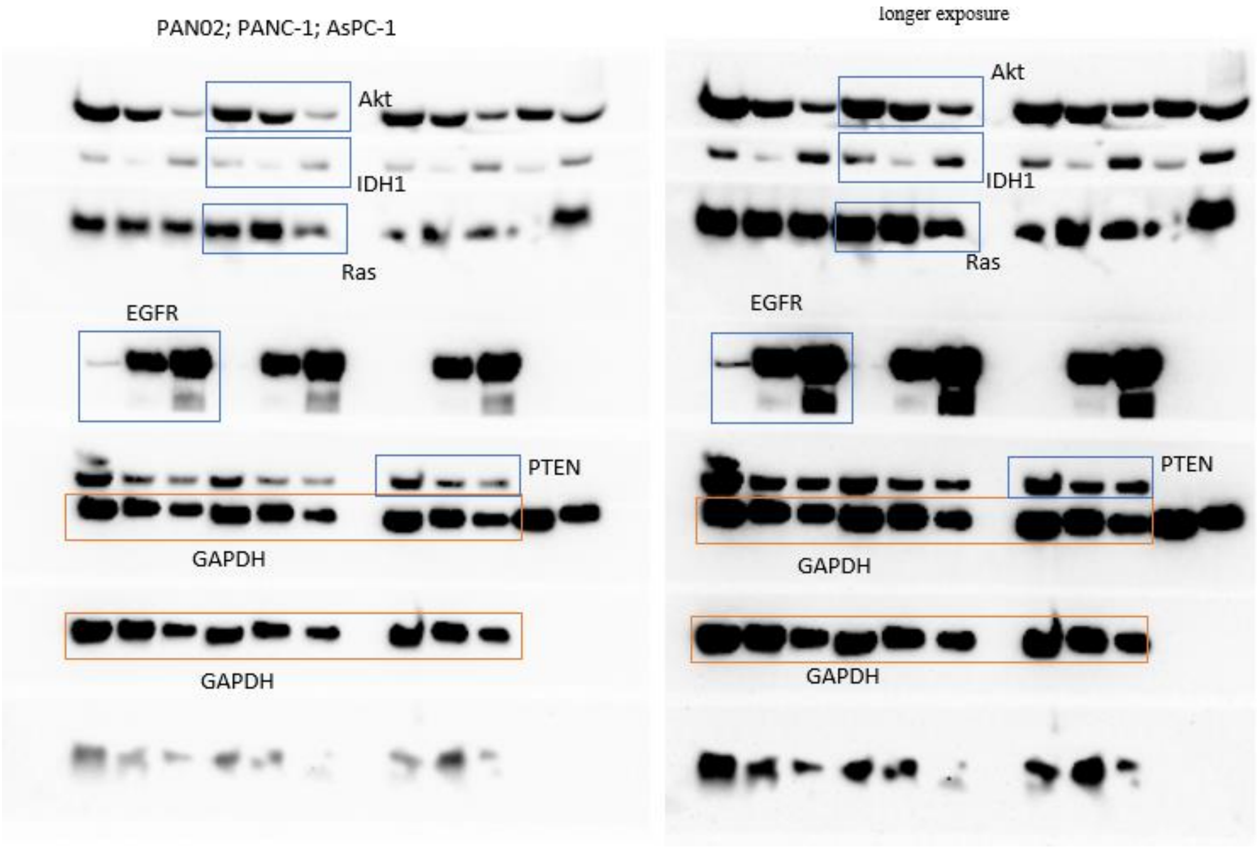

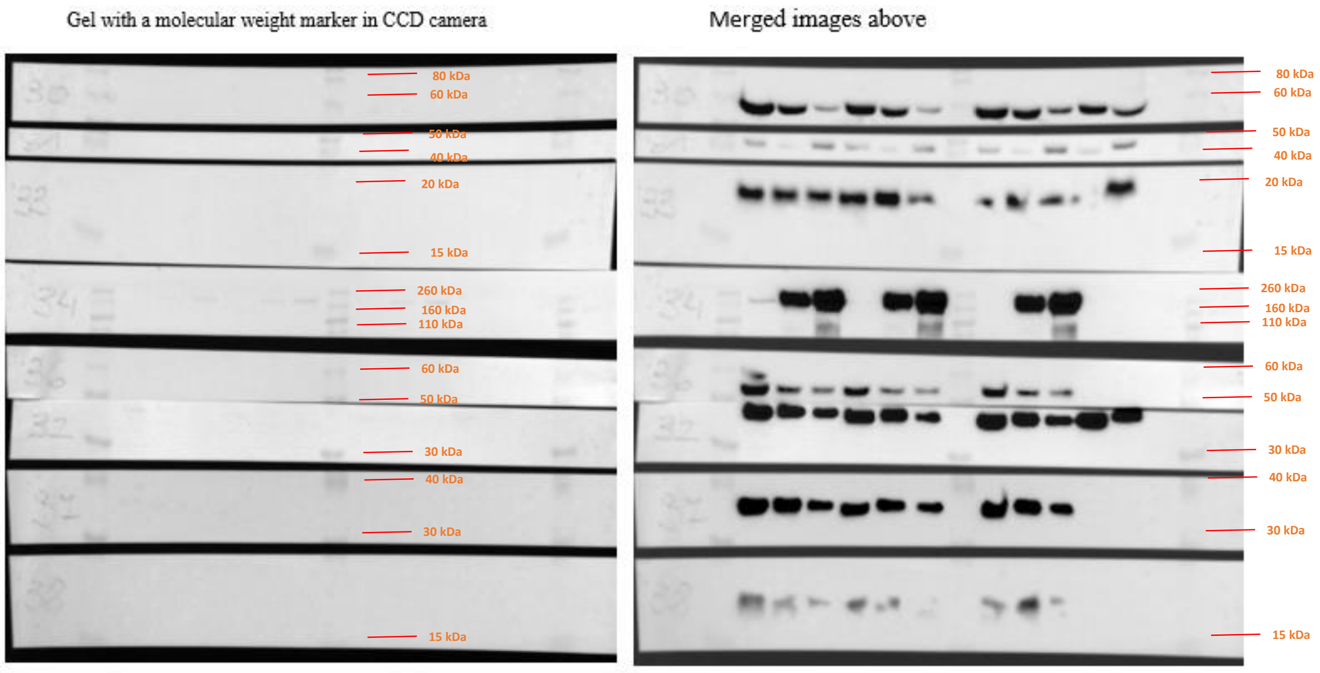

L. Basal expression (Fig. S15) - exposure 30.0 sec + 660.0 sec

We used prestained molecular weight ladder: Spectra Multicolor High Range Protein Ladder (#26625).

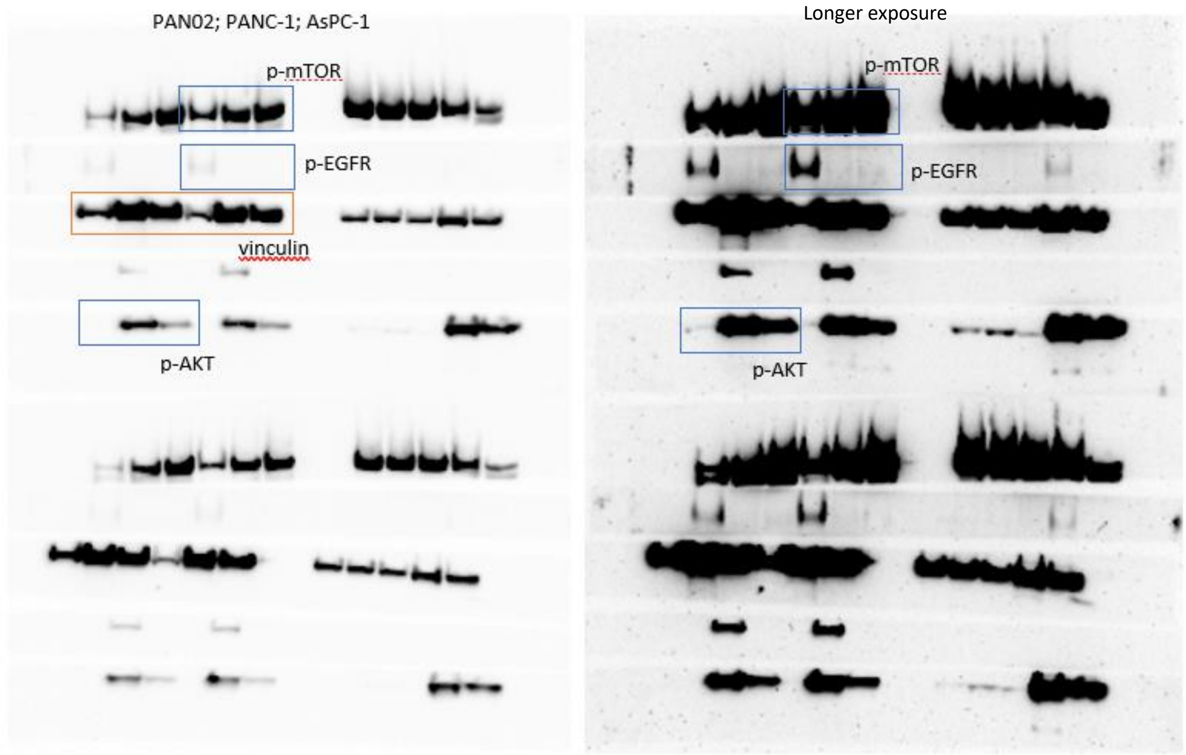

Gel with a molecular weight marker in CCD camera

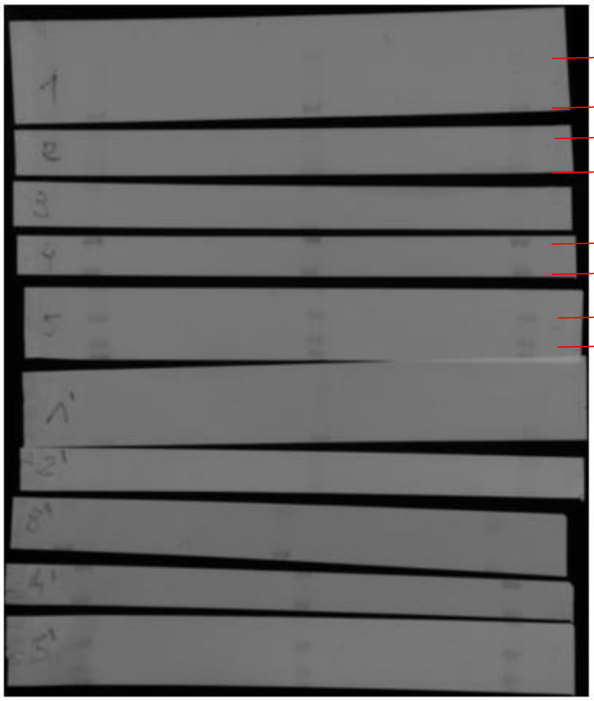

Merged images above

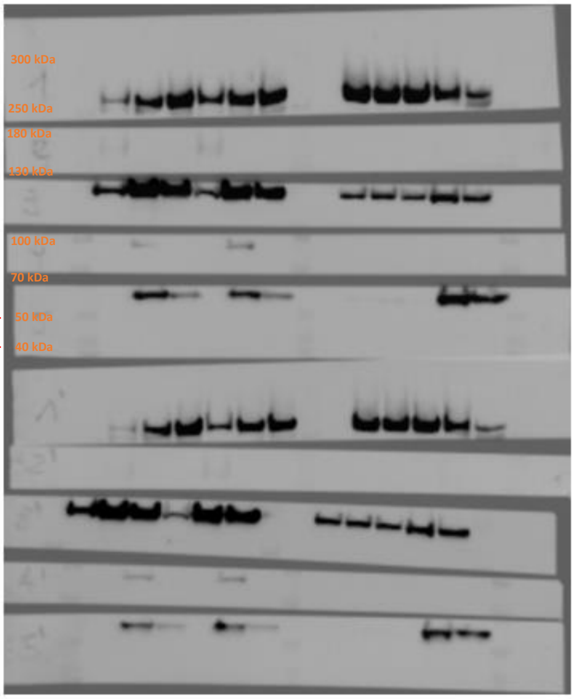

M. Basal expression (Fig. S15) - exposure 120.0 sec

We used prestained molecular weight ladder: Novex Sharp Pre-stained Protein Standard (#LC5800).

PAN02; PANC-1; AsPC-1

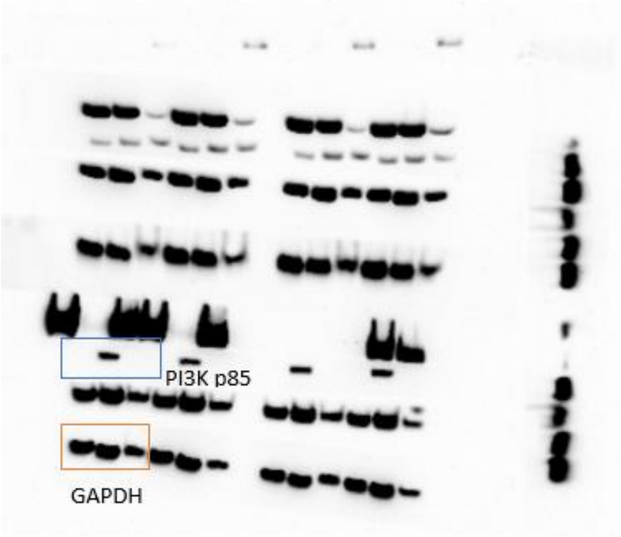

Gel with a molecular weight marker in CCD camera

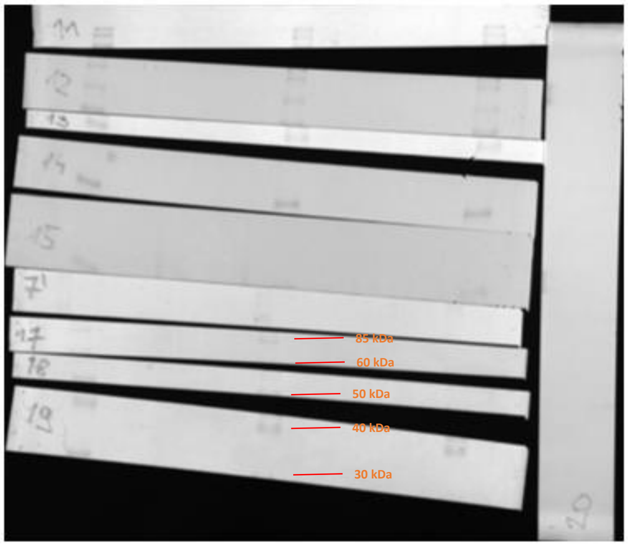

Merged images above

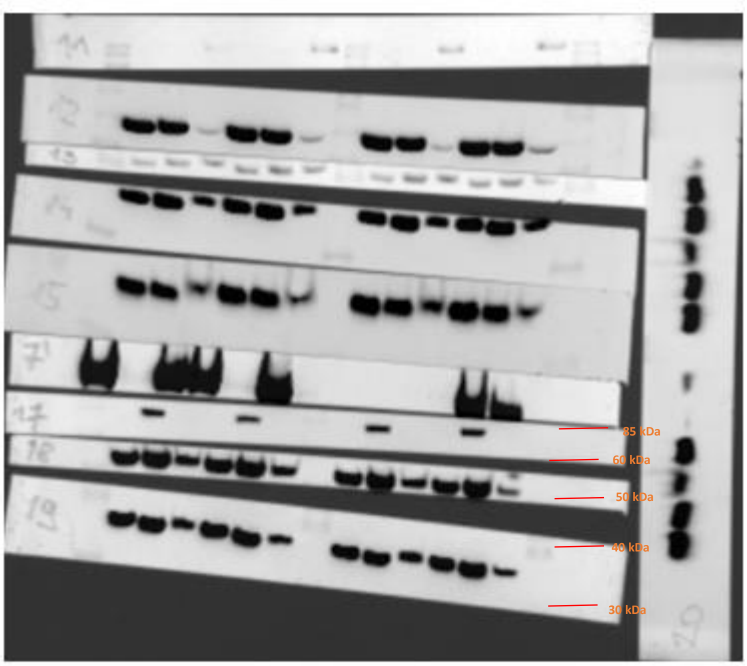

Figure S19

The uncropped and unmodified blots. Proteins relevant to this study are marked in boxes along with reference proteins.

REFERENCES

1. J. Dyke, A. Groves, A. Morris, J. Ogden, A. Dias, A. Oliveira, M. Costa, M. Barros, M. Cabral and A. Moutinho, *Journal of the American Chemical Society*, 1997, **119**, 6883-6887.
